# Multitarget mechanism of MYC inhibition by the bacterial lon protease in disease

**DOI:** 10.1038/s41598-025-88093-2

**Published:** 2025-02-25

**Authors:** Ines Ambite, Murphy Lam Yim Wan, Hien Thi Tran, Atefeh Nazari, Arunima Chaudhuri, Christian Krintel, Inês Gomes, Samudra Sabari, Shahram Ahmadi, António N. B. M. Carneiro, Rita Ishac, Farhan Haq, Catharina Svanborg

**Affiliations:** Department of Laboratory Medicine, Division of Microbiology, Immunology and Glycobiology, Lund University, Klinikgatan 28, Lund, 221 84 Sweden

**Keywords:** Drug development, Experimental models of disease, Infectious diseases, Biotechnology, Computational biology and bioinformatics, Molecular biology

## Abstract

Identifying specific inhibitors of the MYC oncogene has been challenging, due to off target effects associated with MYC inhibition. This study investigated how the recombinant *Escherichia coli* Lon protease (rLon), which targets MYC in human cells, inhibits MYC over-activation in models of infection and cancer. In silico predictions identified specific peptide domains of bacterial Lon that target MYC and the affinity of these peptides for MYC was investigated by surface plasmon resonance. The N-terminal domain of rLon was shown to interact with the C-terminal, leucine zipper domain of MYC and MAX and to prevent MYC/MAX dimerization. Furthermore, rLon targeted and degraded c-MYC in vitro and in cellular models, through the peptidase domain. In a model of kidney infection, rLon treatment prevented, c-MYC, N-MYC and L-MYC over-expression, MYC-dependent gene expression, specifically renal toxicity genes and pathology, suggesting that rLon recognizes and corrects MYC dysregulation in this disease. The findings describe a multitarget mechanism of MYC inhibition by rLon, and the combined effects achieved by the Lon domains, targeting different MYC epitopes and MYC-dependent functions, with no evidence of toxicity or detrimental effects on homeostatic MYC expression.

**Supplementary Information:**

The online version contains supplementary material available at 10.1038/s41598-025-88093-2.

## Introduction

The MYC transcription factors are master regulators of gene expression and tissue homeostasis^1–4^. Transcriptional, translational and post-translational effector functions are regulated by MYC, including all aspects of cell proliferation and cellular growth, which are positively regulated by c-MYC^5–7^. Maintaining control of MYC expression and cellular MYC levels is therefore essential. Indeed, MYC deregulation has been proposed to drive tumor development in > 70% of human cancers, including colorectal and breast carcinomas, prostate and liver cancers^8–10^.

Identifying disease-specific MYC inhibitors has been challenging, and despite extensive efforts, drugs targeting MYC deregulation are not available for clinical use^11–13^. Recent progress includes the Omomyc, a 91-amino acid mini-protein, which acts by blocking the binding of all three forms of MYC to their target promoters and exhibits anticancer activity in preclinical models with minimal side effects^14–18^. Recently, indications of safety and preliminary signs of drug activity have been observed in a first-in-human Phase I clinical trial^19^. Moreover, the recently identified MYCMI-6 and MYCMI-7 compounds, were shown to inhibit MYC/MAX hetero-dimerization and induce c-MYC and N-MYC protein degradation, exhibiting anticancer activity in in vivo tumor models^20,21^. While important, it is unclear to what extent these approaches specifically target MYC deregulation associated with disease and if off target effects of MYC inhibition remain a significant concern.

The ATP-dependent Lon protease is highly conserved in archaea, eubacteria, fungi and mammals, where it functions in protein quality control and cellular stress responses^22–24^. The bacterial Lon protease was recently identified as a potent inhibitor of human MYC, with effects on MYC-dependent gene expression and therapeutic efficacy in models of bladder and colon cancer^25^. This study investigated the mechanism of MYC inhibition by bacterial Lon, the Lon peptide domains responsible for these effects, and if the L-MYC and N-MYC family members are targeted by Lon. We further examined the therapeutic effects of recombinant Lon (rLon) in acute pyelonephritis, where one or both kidneys become infected by bacteria, leading to severe acute infection and a risk for long-term renal impairment^26,27^. The observed efficacy of the MYC inhibitor suggests that rLon may offer an alternative to antibiotics for the treatment of bacterial kidney infections and a tool for achieving MYC inhibition in diseases where MYC is dysregulated.

## Results

### Interactions of the bacterial rLon protein and peptide domains with MYC in silico and in protein binding assays

**Fig. 1:**
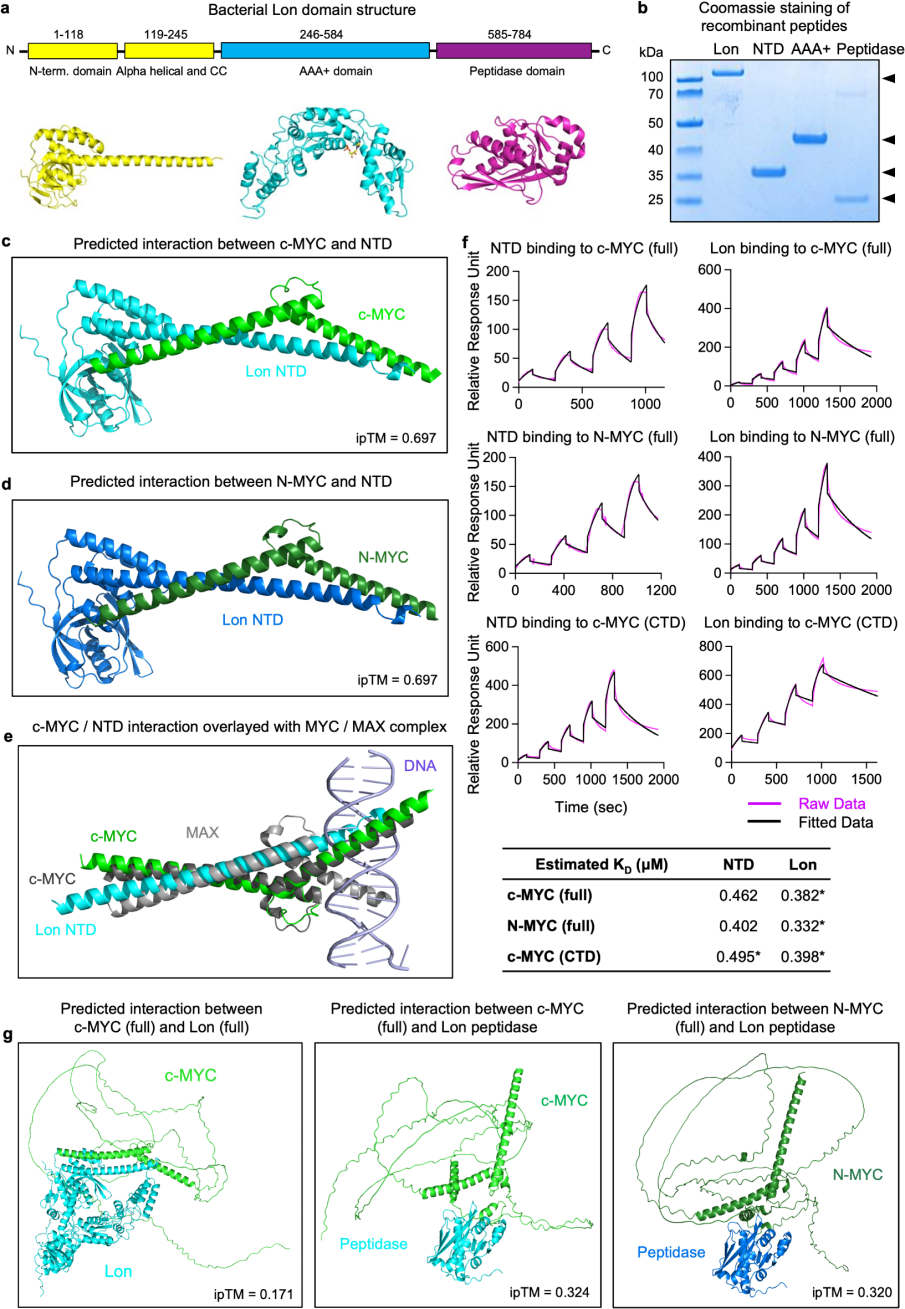
Structure-function analysis of Lon protein interactions with MYC. **a**, Structural overview of bacterial Lon, highlighting the N-terminal domain, the central AAA + domain and the C-terminal peptidase domain. **b**, Purity of the recombinant Lon, N-terminal domain (NTD), AAA + and peptidase domains, verified by Coomassie staining (for Western blot, see Extended Data Fig. 1). **c-e** Interactions of the Lon NTD (blue) with the C-terminal part of (**c**) c-MYC (aa 356–439, green) or (**d**) N-MYC (aa 354–464, forest green). No predicted interaction of the Lon NTD with L-MYC (limon green) by the AlphaFold multimer (Extended Data Fig. 3). **e**, Overlap of the NTD and MAX suggesting that the NTD targets the same binding site in MYC and MAX. **f**, The SPR binding analysis shows the dose-dependent interaction of full-length Lon and NTD peptide to His-tagged c-MYC and N-MYC captured on Sensor Chip NTA (magenta). The data was fitted to a 1:1 Langmuir binding model (black). The table shows the dissociation constant (K_D_) of bindings. * estimated K_D_ values derived from fits where χ² remained over 10% of R_max_. See Extended Data Fig. 5 for fitting parameters. **g**, Interaction of the full-length Lon (blue) with c-MYC (green), Lon peptidase (blue) with c-MYC (green) or N-MYC (forest green).

The bacterial Lon protease comprises an N-terminal domain (NTD), an ATPases Associated with diverse cellular Activities (AAA) domain with an ATP binding site (AAA+) and a peptidase domain (Fig. 1a). To investigate the mechanism of human MYC inhibition by rLon, the *E. coli* Lon protein (aa 1–778), and the NTD peptide (aa 1–245), the AAA + peptide (aa 241–584) and the peptidase domain peptide (aa 581–778) were recombinantly expressed and purified utilizing a C-terminal Strep-tag. The size and purity of the purified recombinant peptides was examined by Coomassie blue staining and Western blot analysis (Fig. 1b and Extended Data Fig. 1).

In silico prediction technology was used to identify MYC protein domains targeted by rLon and the rLon domain peptides (Fig. 1c-e). To probe for interacting epitopes of MYC and rLon, their segmented sequences were submitted in pairs to the AlphaFold multimer protocol. As such, rLon sequence segments comprising the NTD, AAA + domain or peptidase domain were paired with c-MYC segments comprising aa residues 1–110, 89–201, 191–336 or 326–436. The resulting dimeric structures then underwent a sanity check in PyMOL and were ranked in terms of their interface predicted template modelling (ipTM) scores (Extended Data Fig. 2). The highest ipTM score was obtained for c-MYC aa residues 356–439 interacting with the rLon NTD aa residues 191–251 (ipTM = 0.697). N-MYC (aa 354–464) also showed an identical predicted interaction (ipTM = 0.697) with rLon NTD. In contrast, L-MYC (aa 293–364) showed a much weaker interaction with the rLon NTD (ipTM = 0.246) (Extended Data Fig. 3). For the top scoring interactions, the AlphaFold protocol was rerun with two additional seeds, confirming the binding modes reported. Additional seeds overlayed with original seeds are shown in the Extended Data Fig. 4.

**Fig. 2:**
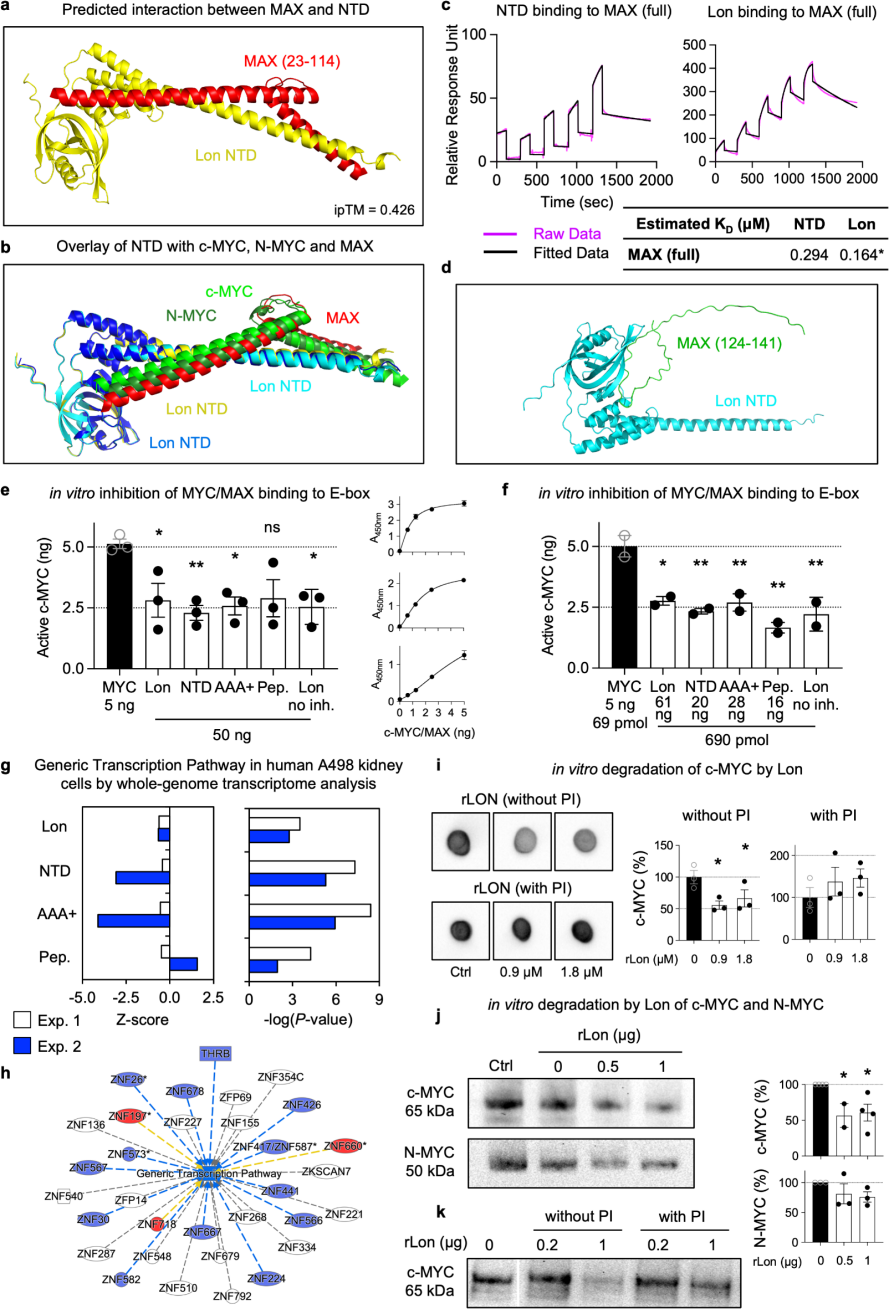
Interaction of rLon and Lon domain peptides with MAX and MYC degradation by Lon and Lon domain peptides. **a**, Interaction of the Lon NTD (yellow) with MAX (aa 23–114, red). **b**, Overlap of the NTD (yellow/blue), c-MYC (green), N-MYC (forest green) and MAX (red). **c**, The SPR binding analysis confirms the interaction of the Lon NTD peptide to His-tagged MAX captured on Sensor Chip NTA (magenta). The data was fitted to a 1:1 Langmuir binding model (black). * estimated K_D_ values derived from fits where χ² remained over 10% of R_max_. See Extended Data Fig. 6 for fitting parameters. **d**, Interaction of the Lon NTD (cyan) with another region of MAX (aa 124–141, green), ipTM values from 0.183 to 0.224. **e**,** f**, Inhibition of c-MYC activation by the NTD and domain peptides as quantified by MYC/MAX complex binding to DNA in an ELISA-based assay. Data are presented as the means ± s.e.m., RM one-way ANOVA followed by uncorrected Fisher’s LSD (**e**), one-way ANOVA followed by Dunnett’s multiple comparisons test (**f**). **g**,** h**, Strong inhibition of the generic transcription pathway by NTD and AAA + in human kidney carcinoma cells treated with the NTD or AAA + domain peptides (cutoff FC ≥ 1.5; *n* = 2 experiments). **i-k**, Analysis of protein degradation by rLon and the peptidase and AAA + domains by dot blot analysis and Western blot. **i**, Dot blot analysis showing direct c-MYC (24 picomoles) degradation by rLon (0.9, 1.8 and 3.6 µM) in the absence of protease inhibitor after 120-minute incubation. **j**, Western blot analysis of c-MYC protein degradation by rLon. c-MYC (50 ng) was degraded by rLon (0.5–1 µg) after 120 min. **k**, This effect was reversed in the presence of protease inhibitors. The 0 µg is cropped from a different part of the gel (white separation line). Uncropped Western blots and replicates are shown in Extended Data Fig. 8. Representative blots are shown (*n* = 2–4 experiments). Data are presented as the means ± s.e.m., RM one-way ANOVA followed by Dunnett’s multiple comparisons test (**i**,** k**), Mixed-effect analysis followed by Holm-Sidak’s multiple comparisons test (**j**).

**Fig. 3:**
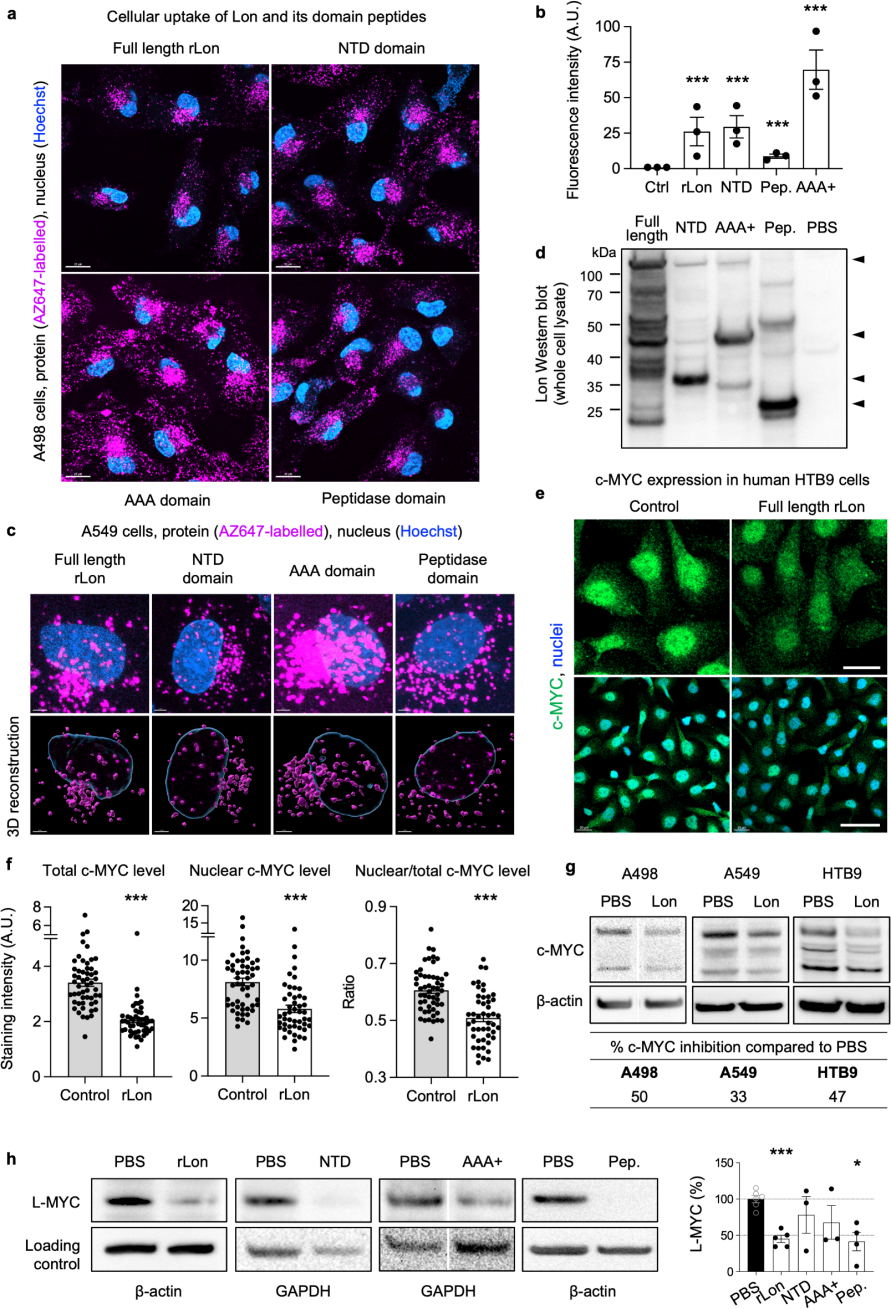
Cellular interactions of rLon and rLon domains and c-MYC protein. **a**, Live-cell confocal images showing the efficient uptake of AZ647-labeled (magenta) rLon and rLon domain peptides (0.902 µM) into the cytoplasm, perinuclear area and nuclei of A498 cells. Nuclei were counterstained with Hoechst (blue). scale bars, 20 μm. **b**, Quantification of the cellular uptake of rLon and rLon domain peptides. Data are presented as the means ± s.e.m., 2-way ANOVA followed by Dunn’s multiple comparisons (*n* = 3 experiments, 50 cells per sample). ***P < 0.001, compared to control. **c**, Live-cell confocal images and 3D reconstructions showing the nuclear and perinuclear uptake of AZ647-labeled (magenta) rLon or Lon domain peptides (0.902 µM) in A549 cells. The nuclear surface is made transparent to visualize the nuclear uptake. Nuclei were counterstained with Hoechst (blue), scale bars, 3 μm. **d**, Western blot confirming the uptake of rLon and rLon domains in whole cell extracts from A498 human kidney carcinoma cells exposed to rLon and its domain peptides. Control lane = PBS-treated cell lysate. **e**, Reduction of c-MYC staining in HTB9 cells treated with rLon (one representative of three experiments). **f**, Quantification of images in (**e**). Data are presented as the means ± s.e.m., total and nuclear c-MYC levels analyzed using Mann-Whitney test, ratio analyzed using unpaired t-test. **g**, Western blot analysis of whole cell extracts. A498, A549 and HTB9 cells were treated with rLon and stained, using the anti-c-MYC primary antibody. Uncropped Western blots and replicates are shown in Extended Data Fig. 14. One representative image is shown, quantification of three experiments. **h**, Western blot analysis of L-MYC in whole cell extracts. Lanes for AAA + are cropped from different parts of the gel (white separation line). Uncropped Western blots and replicates are shown in Extended Data Fig. 15. One representative image is shown, quantification of three experiments. Data are presented as the means ± s.e.m., mixed effect analysis followed by uncorrected Fisher’s LSD.

**Fig. 4:**
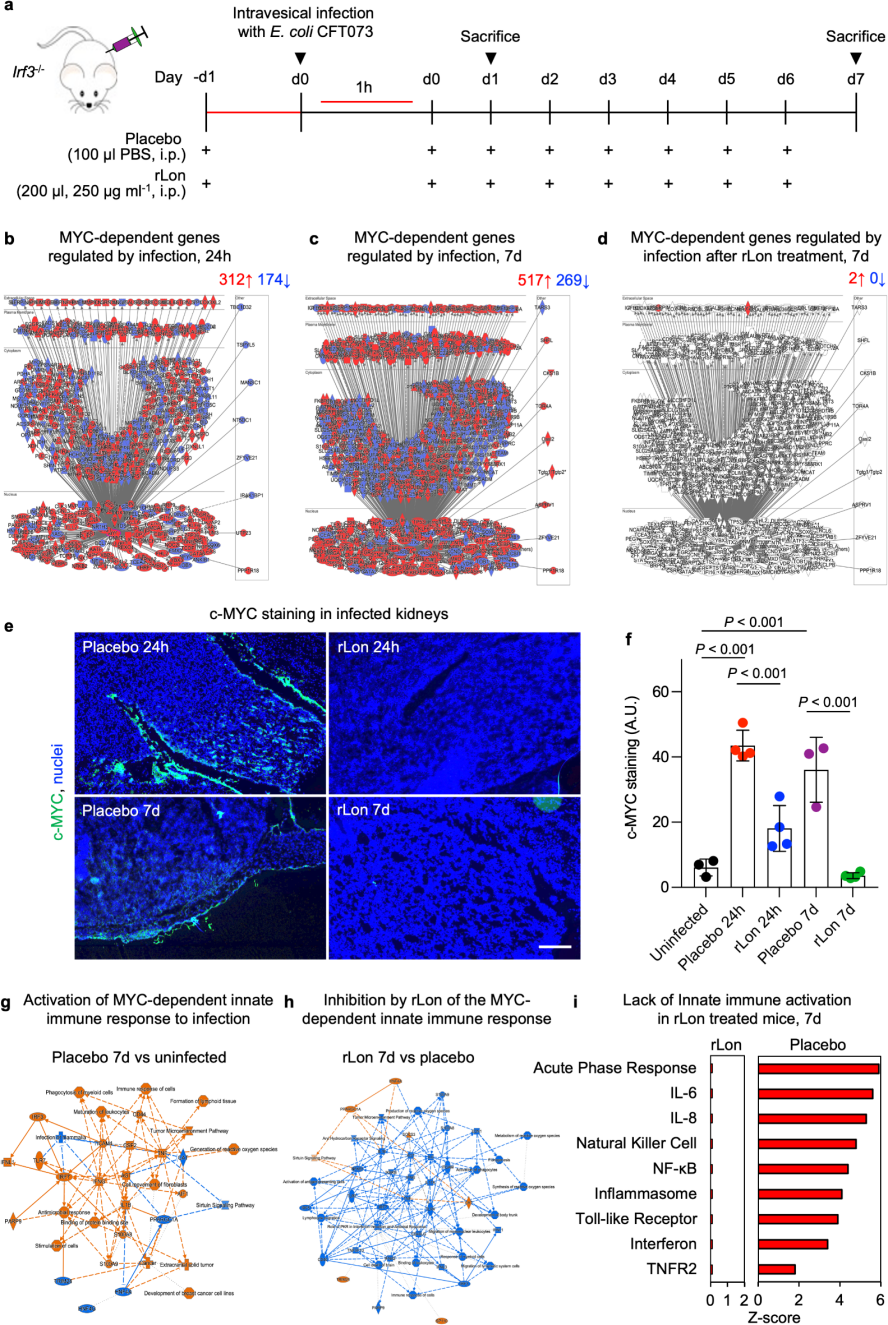
Rapid c-MYC response to kidney infection and inhibition by rLon protease treatment. **a**, Schematic of the protocol used for rLon treatment of *E. coli* CFT073-infected *Irf3*^*−/−*^ mice. Mice were administered rLon intraperitoneally (100 µL, 250 µg/mL) once a day for eight days starting one day before intravesical infection with *E. coli* CFT073. Total renal RNA was isolated from infected mice or uninfected mice after 24 h and seven days and was subjected to genome-wide transcriptomic analysis and disease-associated genes were identified by comparing the infected mice to uninfected controls. **b**,** c**, Response to kidney infection in the placebo group, showing activation of MYC-dependent genes after 24 h and seven days (*n* = 2 per group; red, upregulated; blue, downregulated; cutoff FC ≥ 2, *P* < 0.05 compared to uninfected mice). **d**, Inhibition by rLon treatment of the MYC-response to infection, shown as a marked reduction in the number of MYC-dependent genes in rLon-treated mice compared to the placebo group. **e**, c-MYC protein staining in kidney tissue sections of the rLon-treated group compared to the placebo group. Confocal microscopy; representative images; green, c-MYC; blue, nuclei; scale bar, 20 μm; *n* = 3–7 mice per group. **f**, Quantification of c-MYC protein staining from **e**. Lines represent mean ± s.e.m. One-way ANOVA followed by Tukey’s multiple comparison. **g**,** h**, Functional analysis of MYC-dependent genes, identifying the innate immune response as activated in the placebo group and inhibited in the rLon-treated group. Orange, upregulated; blue, downregulated. **i**, Inhibition of the innate immune response to infection by rLon treatment. The activation Z score for each pathway is shown. Data normalized against healthy, uninfected mice.

As c-MYC is known to interact specifically with the MAX protein^28^, we performed a structural overlay of the c-MYC/MAX complex crystal structure with the predicted interaction site of c-MYC and rLon NTD described above. The overlay identified an almost perfect overlap of NTD with the MAX-binding site in c-MYC (Fig. 1e). This observation suggests that rLon NTD targets the MAX-binding site, thereby displacing MAX and consequently reducing MYC/MAX complex formation. A similar interaction with the MAX-binding site was predicted for N-MYC, with the same ipTM score (Fig. 1d). The results are quantitatively similar to data reported for the binding of Omomyc to MYC with dissociation constant (K_D_) values in the range of 0.2 to 0.5 µM. The corresponding K_D_ numbers for MYCMI-6 are higher (K_D_value of > 1 µM)^20^.

Surface plasmon resonance (SPR) analysis, by Biacore technology, was further used to quantify the predicted interactions. NTA sensor chips were charged with Ni ions, followed by capture of each His-tagged ligand (full length c-MYC, C-terminal c-MYC, and full-length N-MYC). The efficiency of capture was evaluated to optimize the capture concentration used for subsequent experiments. Sample was then injected over the captured Histidine (His)-tagged ligand and binding was monitored at five different concentrations (for a summary of SPR data, please see Extended Data Figs. 5 and 6).

**Fig. 5:**
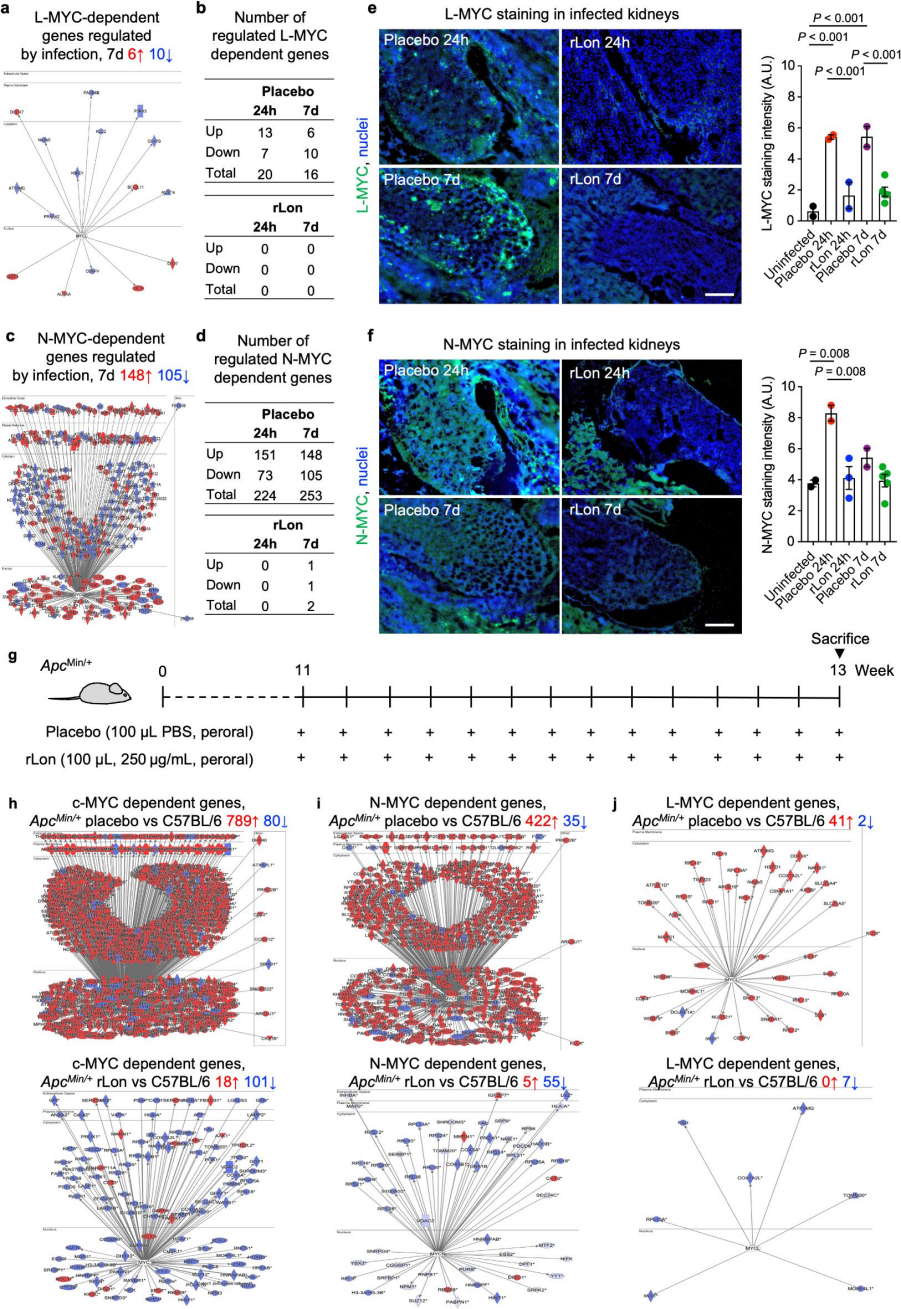
Activation of L-MYC and N-MYC is inhibited by rLon treatment in the infection model. **a**,** b**, Network analysis of L-MYC-dependent genes regulated by *E. coli* CFT073 infection in *Irf3*^*−/−*^ mice. The L-MYC response to kidney infection was inhibited by rLon treatment, with none of L-MYC-dependent genes differentially expressed after 24 h and after seven days (*n* = 2 mice per group; cutoff FC ≥ 2; *P* < 0.05 compared to uninfected mice). **c**,** d**, The N-MYC response to kidney infection was inhibited by rLon treatment, with none of N-MYC-dependent genes differentially expressed after 24 h and two after seven days (*n* = 2 mice per group; cutoff FC ≥ 2; *P* < 0.05 compared to uninfected mice). **e**,** f**, Reduced L-MYC staining and N-MYC staining in kidney sections obtained from rLon-treated mice visualized by immunohistochemistry (Confocal microscopy; representative images; green, N-MYC or L-MYC; blue, nuclei; scale bar, 20 μm; *n* = 2–5 mice per group). Lines represent mean ± s.e.m. Kruskal-Wallis followed by Dunn’s multiple comparison test. **g**, Schematic of the protocol used for rLon treatment of cancer-prone *Apc*^Min/+^ mice by oral gavage (100 µl, 250 µg/mL) twice daily for 14 days, starting at 11 weeks of age. **h-j**, Strong activation of c-MYC-, N-MYC- and L-MYC-dependent genes in *Apc*^Min/+^ mice compared to disease-free C57BL/6 control mice. Inhibition of c-MYC-, N-MYC- and L-MYC-dependent genes in rLon-treated *Apc*^Min/+^ mice (*n* = 2 mice per group; cutoff FC ≥ 2; *P* < 0.05 relative to disease-free C57BL/6 control mice).

**Fig. 6:**
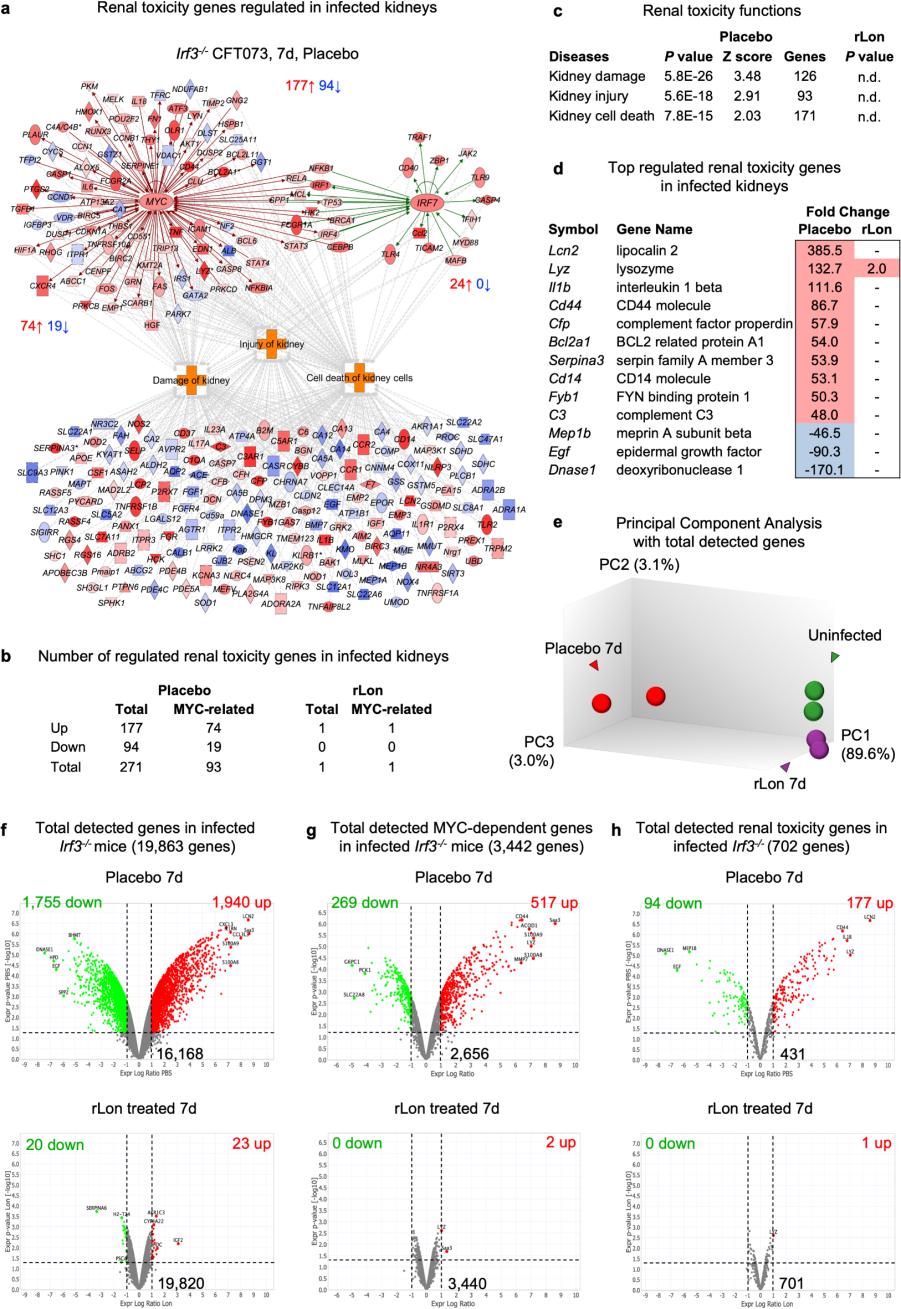
Inhibition of nephrotoxicity by rLon in *Irf3*^***−/−***^mice and proximity of treated mice to uninfected mice. **a**, Gene expression analysis identifying genes associated with renal toxicity, renal injury or kidney-specific cell death in *E. coli* CFT073-infected *Irf3*^*−/−*^ mice. Strong activation of MYC- and IRF7-dependent genes regulating renal injury or kidney-specific cell death in the placebo group compared to the uninfected group. Red, upregulated, blue, downregulated; cutoff FC ≥ 2; *P* < 0.05 compared to the uninfected group. **b**, Complete inhibition of genes regulating renal injury or kidney-specific cell death in the rLon-treated group compared to the placebo group. **c**, Inhibition of kidney damage, injury and cell death pathways by rLon treatment compared to placebo. **d**, Top regulated renal toxicity genes in the placebo group were not regulated in the rLon-treated group, with the exception of *Lyz*. **e**, Principal component analysis of the whole kidney RNA data sets. The right cluster shows uninfected mice (green) and the adjacent cluster shows the rLon-treated mice (purple). The left cluster shows the placebo group (red). **f-h**, Volcano plots of all the detected genes (**f**), all MYC-dependent genes (**g**) and all renal toxicity genes (**h**) (*n* = 2 per group; FC vs. uninfected; white, no change; red, activated; blue, inhibited). Two gene categories were distinguished in the placebo group; category one was the strong disease response (*n* = 3,695) and category two was the remaining genes that were not regulated by disease (*n* = 16,168). Category one (disease related genes) was inhibited in rLon-treated mice (*n* = 43) but category two (genes unrelated to disease) was not significantly regulated by rLon treatment (*n* = 19,820). The MYC-dependent genes and renal toxicity genes showed a similar profile in the rLon-treated group and resembled that in the uninfected control group.

The interaction of the rLon protein or the Lon peptides with the His-tagged full-length c-MYC or N-MYC proteins captured on the Ni-NTA sensor chips, is shown in Fig. 1f and Extended Data Fig. 5. The NTD peptide was shown to bind to c-MYC and N-MYC, in a dose-dependent manner, with a K_D_ < 1 µM. In addition, dose dependent binding of rLon to c-MYC and N-MYC was observed, with a higher contribution of unspecific binding. Furthermore, the NTD peptide and rLon were shown to bind the C-terminal domain of MYC, which is highly conserved in c-MYC and N-MYC (Fig. 1f). The affinity of NTD was similar to that reported for MYCMI-6, a small molecule inhibitor of MYC-driven transcription, which binds to the MYC basic helix-loop-helix (b-HLH) domain^20^. Full-length L-MYC protein, was not available for SPR analysis.

Interactions were further predicted between the rLon peptidase domain and the full-length c-MYC, N-MYC and L-MYC proteins, with lower ipTM scores than for the NTD peptide (ipTM = 0.324, 0.32 and 0.3, Fig. 1g and Extended Data Fig. 3). The AlphaFold multimer protocol further predicted a low score for the interaction between full-length c-MYC and full-length rLon (ipTM = 0.171), and SPR analysis detected a dose-dependent binding of the peptidase- and AAA + domains to full-length c-MYC, with a high contribution of non-specific binding (Extended Data Fig. 3).

### Interaction of rLon with MAX

The AlphaFold multimer protocol further predicted the formation of an NTD-MAX dimer, with a weaker ipTM score (ipTM = 0.426) than for the NTD-c-MYC dimer (aa 23–114, ipTM = 0.697, Fig. 2a, b). Binding of rLon and NTD to MAX was confirmed by SPR analysis (Fig. 2c). Dose dependent binding of NTD to MAX demonstrated a K_D_ < 1 µM. A second MAX region (aa 124–141) was predicted to interact with the N-terminal part of NTD, with a lower ipTM score (ipTM = 0.224 to 0.183) (Fig. 2d). Repeated SPR analysis confirmed the precited K_D_ (Extended Data Fig. 6).

The molecular interfaces, predicted using AlphaFold, suggest that the NTD binds c-MYC, N-MYC, and MAX in an almost identical manner (Figs. 1c-e and 2a-b). The ipTM scores for the complexes are identical for c-MYC and N-MYC (0.697) with MAX in the same range (0.426). SPR measurements confirmed the affinity of the NTD for MAX, c-MYC and N-MYC (Fig. 2c) indicating that the NTD might target the helix-loop-helix structures shared by these proteins, consistent with previously published K_D_of the MAX-c-MYC complex of 0.167 µM^29^. As previously shown, there was no evidence of MAX degradation by rLon^25^.

### Inhibition of MYC-MAX dimerization by rLon and rLon domain peptides

The in silico predictions and SPR analyses further suggested that the binding of Lon or its NTD domain would reduce MYC/MAX complex formation. This hypothesis was addressed, using an ELISA-based TransAM c-MYC assay to quantify c-MYC/MAX binding, by detecting c-MYC binding to its consensus E-box DNA sequence. The rLon protein and the NTD peptides were shown to significantly reduce the binding as well as the AAA + peptide, which has affinity for DNA^30^ (Fig. 2e). The effect of the peptidase domain was increased at lower peptide concentration (Fig. 2f). The competitive inhibition shown by ELISA, supports the results obtained by in silico prediction and SPR analysis and the hypothesis that rLon and its domains inhibit MYC/MAX complex formation.

### Inhibition of gene expression by rLon and rLon domain peptides

The inhibition of MYC/MAX complex formation predicted that gene expression would be affected in rLon treated or Lon peptide-treated cells. To address this question, effects on gene expression were quantified by transcriptomic analysis. Total RNA was extracted from human kidney carcinoma cells exposed to rLon or the NTD, peptidase or AAA + peptides and cells treated with phosphate buffered saline (PBS) were used as a control. Significantly expressed genes in treated cells compared to control were identified (Fig. 2g, h). Functional analysis of the significantly regulate genes identified the generic transcription pathway as strongly downregulated in cells treated with the NTD or AAA + peptides, with a weaker effect of rLon and the peptidase domain (Fig. 2g). Inhibited genes further included zinc finger proteins (ZFPs) (Fig. 2h), which are known regulators of the transcriptional machinery^31^.

### Direct MYC protein degradation by Lon *in vitro*

 MYC degradation by Lon was quantified *in vitro*, by mixing of rLon with recombinant c-MYC or N-MYC in the presence of ATP and Mg^2+^. A significant reduction in c-MYC protein levels was detected by dot blot analysis after 120 min of incubation with rLon at 37 °C, compared to PBS (Fig. 2i and Extended Data Fig. 7). The reaction mixture was further subjected to Western blot analysis, using the same protocol, and the reduction in MYC levels was confirmed compared to PBS (Fig. 2j and Extended Data Fig. 8). The effect was not detected in the presence of a protease inhibitor cocktail (Fig. 2k and Extended Data Figs. 7 and 8). Full-length L-MYC was not available for in vitro analysis.

### Cellular uptake of rLon and rLon peptide domains

 Live cell confocal imaging was used to examine if the bacterial Lon protein and its peptides reach the interior of human cells. N-terminal labeling was performed with the AZ647 NHS ester dye at high pH, and free dye was removed by overnight dialysis followed by centrifugation in an Amicon filter unit. The concentration of the labelled complex was determined using NanoDrop. The endogenous human Lon protein would not be detected in these experiments, as it was not labelled. Control experiments did not detect the AZ647 NHS Ester free dye in the treated cells (Extended Data Figs. 9 and 10). The labelling efficiency of rLon and the peptides is shown in the Extended data Fig. 10. The cellular shape and outline of the cells was visualized by bright-field microscopy.

Human kidney carcinoma cells (A498) were treated with AZ647-labeled rLon, NTD, AAA + or peptidase (0.9 µM) and the cellular uptake of rLon was investigated by live cell confocal imaging (Fig. 3a and b). A time dependent increase in cellular uptake from 10, to 30 and 60 min or 3.5 h is shown in Extended Data Fig. 9. Lon staining was detected in the cytoplasm, perinuclear and nuclear areas, with a predominance of a vesicular staining pattern, possibly indicating endocytosis. Each of the AZ647-labeled peptides showed a similar uptake and cellular distribution pattern as rLon (Fig. 3a).

 The uptake of rLon and the NTD, AAA + and peptidase peptides was confirmed in A549 lung carcinoma cells (Fig. 3c and Extended Data Fig. 10). The perinuclear accumulation and nuclear uptake of rLon was confirmed by 3D reconstruction technology in cells with transparent nuclei (Fig. 3c). Cellular uptake of rLon or the NTD, AAA + and peptidase domain peptides (0.9 µM) was further confirmed by Western blot analysis of A498 and A549 whole cell extracts (Fig. 3d and Extended Data Fig. 11). The results suggest that rLon could affect targets such as MYC in several cellular compartments.

### Effects of rLon on cellular MYC levels

To quantify overall effects on cellular c-MYC, N-MYC and L-MYC protein levels, human carcinoma cell lines were treated with rLon, fixed and imaged by confocal microscopy, after staining with antibodies specific for the c-MYC N-terminal domain. A significant reduction in total and nuclear c-MYC levels was detected in rLon treated cells, and the ratio of nuclear versus cytoplasmic staining was reduced (Fig. 3e, f and Extended Data Figs. 12 and 13). A reduction in c-MYC levels by rLon and the peptidase domain was confirmed in rLon treated cells, by Western blot analysis of whole cell extracts (Fig. 3g and Extended Data Fig. 14). A strong reduction in cellular L-MYC levels was observed in cells treated with rLon as well as the NTD, peptidase or AAA + domains (Fig. 3h and Extended Data Fig. 15).

The results confirm that rLon treatment reduces cellular MYC levels and identifies rLon peptides as MYC inhibitors in treated cells.

### Comparison of bacterial and human Lon

The amino acid sequence and domain organization of bacterial Lon has been compared to the human Lon proteases, LONP1 and LONP2^32,33^, which are localized in mitochondria or peroxisomes. The three proteins maintain a similar overall architecture and belong to the type A Lon proteases (Extended Data Fig. 16). Sequence homology of the human and bacterial proteins is particularly observed in the AAA + and peptidase domains, with lower conservation in the N-terminal regions^32^.

To investigate if cross-reactivity with human Lon would influence the outcome of these experiments, the specificity of the antibodies to bacterial Lon was examined by Western blot analysis. Antibodies to bacterial Lon were shown to detect rLon and the NTD, AAA + and peptidase domain peptides. In contrast, the antibodies did not react with whole-cell lysates, where human Lon is present. Furthermore, the human LONP2 antibody did not react with the recombinant bacterial proteins and the human LONP1 antibody detected a weak band of about 100 kDa, but no bands corresponding to the peptides, suggesting that cross-reactivity of the anti-bacterial Lon antibodies would not affect the analyses in this study (Extended Data Fig. 16).

### The MYC response to infection is inhibited by rLon treatment in disease-prone mice

rLon treatment was previously shown to inhibit *MYC *expression and reduce MYC protein levels in vivo, resulting in protection in two murine cancer models^25^. In view of the importance of MYC for renal development and the bacterial origin of the Lon protein, this study investigated if rLon affects *MYC* expression and disease severity, in a model of acute bacterial infection affecting the kidneys^26,27^. The susceptibility to kidney infection is increased in mice carrying deletions in the interferon regulatory factor 3 gene (*Irf3*) and *Irf3*^*−/−*^mice provide a relevant model of human disease^33,34^.

The MYC response to renal infection was investigated in *Irf3*^*−/−*^ mice, infected by injection of the uropathogenic *E. coli* strain CFT073 into the urinary tract (Fig. 4a). After 24 h or seven days, mice were sacrificed using an isoflurane euthanasia overdose and tissue samples were collected for further analysis. Total renal RNA harvested from infected mice or uninfected mice was subjected to genome-wide transcriptomic analysis and disease-associated genes were identified after 24 h and seven days, by comparing the infected mice to uninfected controls.

The change in gene expression in response to infection is shown in Fig. 4b, 24 h or seven days post infection. Infection increased the expression of *Myc* and MYC-dependent genes, predicted to affect all cellular compartments (Fig. 4b, c). The majority of those genes were strongly activated compared to uninfected controls (FC > 2, *P* < 0.05), with similar kinetics for total gene expression and MYC-dependent genes (Fig. 4b, c, Extended Data Fig. 17). A parallel increase in renal c-MYC protein levels was detected in tissue sections from infected *Irf3*^*−/−*^ mice, compared to uninfected controls, specifically along the mucosal lining of the renal pelvis and in the renal papillae, which are primary infection sites (Fig. 4e, f and Extended Data Fig. 18). A similar increase in MAX protein levels was detected by immunohistochemistry in the renal mucosa of infected mice (Extended Data Fig. 19).

To investigate whether rLon acts as a MYC inhibitor in this model, *Irf3*^*−/*−^ mice were intra-peritoneally injected with rLon (250 µg/mL) once a day for eight days, starting one day before infection (Fig. 4a). The placebo group received PBS and RNA was extracted from renal tissues at sacrifice, as described above. The expression of MYC-dependent genes was virtually abolished in the rLon-treated group on day 7. Only two genes were significantly regulated in treated mice, compared to 786 genes in the placebo group (Fig. 4c, d and Extended Data Figs. 20, 21 and 22). In rLon-treated infected *Irf3*^*−/−*^ mice, renal tissues analysis by immunohistochemistry revealed a decrease in c-MYC staining compared to the placebo group, confirming the treatment effect at the protein level (Fig. 4e, f and Extended Data Fig. 18). A similar decrease in MAX protein levels was detected by immunohistochemistry in the renal mucosa of rLon treated, infected mice (Extended Data Fig. 19).

Functional analysis of the MYC-dependent genes identified innate immunity as the most strongly regulated gene category in the placebo group (Fig. 4g and Extended Data Figs. 20, 21 and 22), with activation of c-MYC-dependent regulators of innate immunity; toll-like receptors (TLR), interleukin (IL)−1β, tumor necrosis factor (TNF), type I interferons (IFN), as well as calcium binding proteins (S100A8, S100A9), which activate neutrophil recruitment^35^. rLon treatment inhibited this MYC-dependent innate immune response, compared to placebo (Fig. 4h and Extended Data Figs. 20, 21 and 22). Major innate immune signaling pathways, were no longer regulated in rLon-treated mice compared to healthy mice, including the IFN, TLR, NF-κB and inflammasome signaling pathways (Fig. 4i and Extended Data Fig. 23). This suppression of the innate immune responses in the rLon-treated mice was further analyzed by Gene Set Enrichment Analysis (GSEA), which confirmed the downregulation of critical immune pathways and MYC target genes (Extended Data Fig. 24).

### rLon treatment inhibits the activation of L-MYC and N-MYC in models of infection and cancer

To investigate if the effect of rLon extends to additional MYC family members, L-MYC- and N-MYC-dependent gene expression were compared between the infected-rLon treated and placebo groups (Fig. 5a-d and Extended Data Fig. 25). In the placebo group, L-MYC and N-MYC dependent genes were strongly activated after 24 h and seven days compared to uninfected controls. By immunohistochemistry, increased L-MYC and N-MYC staining was along the renal pelvis and in the papillae, confirming the N-MYC and L-MYC response at the protein level in infected *Irf3*^*−/−*^ mice (7 days; Fig. 5e, f and Extended Data Figs. 26 and 27).

rLon treatment virtually abolished the L-MYC and N-MYC response to infection (Fig. 5a-f and Extended Data Figs. 25, 26 and 27). There was no differentially expressed L-MYC-dependent gene and only two N-MYC-related genes in rLon-treated mice, seven days after infection (Fig. 5b, d) and L-MYC and renal N-Myc staining was inhibited (Fig. 5e, f). The results suggest that N-MYC and L-MYC are activated by infection in *Irf3*^*−/−*^ mice and that rLon inhibits N-MYC and L-MYC deregulation in infected kidneys. A similar effect of rLon treatment was detected in *Apc*^*Min/+*^ mice^26^, which develop c-MYC-dependent multiple adenomas, mostly in the small intestine, due to a mutation in the *Apc*tumor suppressor gene^36,37^ (Fig. 5g-j).

A direct anti-bacterial effect of rLon was excluded by culturing *E. coli* CFT073 in vitro in the presence of rLon (250 and 500 µg/mL). Whereas the antibiotic cefotaxime (100 mg/mL) reduced bacterial numbers in vitro after 3.5 h, there was no antibacterial effect of full-length rLon, NTD, AAA + or the peptidase domain (Extended Data Fig. 28).

### Effects of rLon treatment in healthy mice

To examine if rLon treatment affects homeostatic MYC gene expression, healthy C57BL/6 mice were treated with rLon i.p. according to the protocol shown in Fig. 4a (Extended Data Figs. 29, 30 and 31). A modest regulation of c-MYC-dependent genes was observed after 24 h and seven days with a predominance of inhibition (Extended Data Fig. 30). Few N-MYC-dependent genes and only two L-MYC-dependent genes were regulated, suggesting a low effect of rLon treatment on MYC homeostasis in the absence of disease.

### rLon-treated mice are protected from renal toxicity

The low response to rLon treatment in healthy mice suggested that rLon may target changes in MYC during disease. To further analyze the disease response in infected kidneys, established renal disease parameters were compared between the rLon-treated mice and the placebo group.

rLon treatment was shown to prevent infected *Irf3*^*−/−*^ mice from developing severe acute kidney disease, defined by gross pathology scores (abscess formation, edema, hyperemia) (Extended Data Fig. 32). Moreover, neutrophil infiltration and abscess formation were inhibited, and neutrophil counts in urine were reduced in the treatment group (Extended Data Fig. 32). In parallel, quantitative cultures of urine and infected renal tissues showed that bacterial clearance from the kidneys was accelerated in the rLon treatment group compared to the placebo group (Extended Data Fig. 32).

Acute pyelonephritis is an important cause of acute and lasting renal damage^38^. We therefore investigated if genes that regulate renal toxicity are affected by rLon treatment. A significant number of renal damage-associated genes were regulated in the placebo group compared to the uninfected control group and 93/271 of those genes were defined as MYC-related (34%, Fig. 6a, b and Extended Data Fig. 33). In contrast, only one gene associated with renal toxicity was significantly regulated in the rLon-treated group, and there was no evidence of a broader MYC-dependent renal damage response (Fig. 6a-d and Extended Data Fig. 33). The results suggest that rLon targets and inhibits genes involved in renal toxicity, renal injury or kidney-specific cell death, which drive renal damage.

### Lack of off target effects on gene expression in the kidneys of rlon-treated mice

The information provided by genome wide transcriptomic analysis of rLon treated mice and controls was further used to investigate overall effects on homeostatic gene expression in the disease prone mice. To visualize if rLon treatment affects gene expression unrelated to infection, Principal component analysis (PCA) was performed. PCA of the RNA data set showed that the rLon-treated group clustered adjacent to that of uninfected mice, while data for the placebo group clustered at a significant distance from the healthy mice (90% and 86% variance, 1st principal component, Fig. 6e and Extended Data Fig. 33). The gene expression profile of treated infected mice was similar to uninfected mice, suggesting that gene expression unrelated to disease is not significantly affected by rLon treatment.

Furthermore, Volcano plots were constructed to provide an overview of the response in infected mice, and the lack of change in rLon treated mice, compared to healthy mice (Fig. 6f-h). The disease response, comprised a total of 3,695 genes, including 786 MYC-related genes. Very few of these genes were differentially expressed in the rLon-treated mice (*n* = 43) compared to uninfected *Irf3*^*−/−*^ mice (Fig. 6f-h), illustrating the highly efficient targeting by rLon of the disease-associated increase in gene expression that occurred in the placebo group. These results indicate that rLon inhibits MYC deregulation caused by infection, without causing major disruptions of healthy MYC gene expression machinery.

## Discussion

The basic helix-loop-helix (bHLH) leucine zipper (LZ) domain of c-MYC is crucial for MYC/MAX hetero-dimerization and subsequent DNA binding that initiates MYC-dependent transcription^39^. In silico analysis predicted that rLon or the NTD peptide bind to the c-MYC-MAX binding site, thereby competitively inhibiting MYC/MAX hetero-dimerization. SPR analysis confirmed the high affinity interactions of NTD peptide with c-MYC, providing a basis for the inhibition of MYC/MAX dimerization. Inhibition of MYC/MAX dimerization and DNA binding was further documented using an ELISA assay, where rLon, NTD as well as peptidase and AAA + domains showed inhibitory activity. The K_D_ values obtained for NTD binding to full length c-MYC were below 1 µM, potentially lower than the small molecule inhibitor MYCMI-6 with a K_D_value of 1–2 µM for the bHLH-LZ domain of c-MYC, which inhibited MYC/MAX dimerization (aa 354–437)^20^. The rLon NTD peptide was further predicted by in silico analysis to bind MAX, and binding was validated by SPR analysis and dot blots. Based on the in silico prediction models, SPR data and competitive inhibition shown by ELISA, we suggest that rLon and its domains inhibit MYC/MAX complex formation, which would explain the potent inhibition of MYC-dependent gene expression in rLon-treated mice with cancer or infection.

c-MYC is a short-lived protein and its degradation by the ubiquitin-proteasome pathway depends on the phosphorylation state of Threonine-58 (Thr-58) and Serine-62 (Ser-62)^40,41^. c-MYC is targeted for degradation by the ubiquitin ligase Fbw7, which recognizes the phosphorylated Thr-58 residue^40^. Degradation of c-MYC is inhibited by de-ubiquitination, and the ubiquitin-specific proteases USP28 and USP36 increase c-MYC stability by deubiquitination, protecting c-MYC from degradation^42,43^. MYC phosphorylation, ubiquitination and proteasome degradation has been extensively studied to gain control of MYC levels in cancer cells, with several compounds in preclinical development or early clinical trials^11,13,43^, including all-trans retinoic acid (ATRA) and Vismodegib, which are approved by the FDA for the treatment of acute myeloid leukemia and basal-cell carcinoma^44,45^. In this study, the rLon protease was shown to directly degrade MYC in vitro, in the presence of ATP but in the absence of any cellular constituents^46,47^. A possible explanation for the rLon effect would be that the bacterial rLon peptidase, with its inherent function as a scavenger of unfolded proteins, is able to degrade disordered MYC domains, thus circumventing the specifically mammalian phosphorylation checkpoints that would otherwise protect dysregulated MYC^13,48–50^.

The human and bacterial LON proteases are essential for protein quality control and are involved in complex cycles of protein unfolding and degradation^51^but their roles differ significantly due to their distinct functions^32^. In humans, two LON isoforms share domains for substrate recognition, ATPase activity, and proteolysis, specifically targeting proteins within mitochondria or peroxisomes^51,52^. The bacterial Lon proteases, which have a similar domain structure as the human LON proteins, target unfolded proteins in bacteria and are crucial for bacterial survival under stress conditions^53^. In this study, there was no evidence that cross-reactivity between the bacterial rLon and rLon peptides with the human LONP1 or LONP2 proteins would influence the observed cellular effects of the bacterial rLon protein or its peptides. Antibodies to bacterial Lon showed a high level of specificity, and antibodies to the human LONP1 and LONP2 proteins showed no or limited reactivity with the bacterial proteins. Furthermore, there was no evidence that mammalian LON expression was regulated in mice treated with the bacterial rLon protein.

SPR assays are pivotal for analyzing biomolecular interactions. This study used the NTA Sensor Chip based binding assay to validate the alpha-fold predictions. Ni-NTA and the antibody based His-tag captures are used for immobilizing histidine-tagged proteins and offers distinct advantages particularly when conducting kinetic analyses that require regeneration of the surface between each concentration, performed in this manuscript. NTA Sensor Chips offer superior control over protein orientation and density by enabling uniform immobilization through the His-tag and allowing tunable density adjustments to minimize steric hindrance and non-specific interactions. In contrast, antibody-based capture can affect protein orientation and reduce assay reproducibility due to multiple conformations of antibody-protein complexes. However, NTA-based surfaces are susceptible to non-specific interactions and baseline increases are an inherent feature of the assay, as can be seen from the fitted curves. These interactions reduce specificity, and lower the signal-to-noise ratio, potentially affecting the accuracy of kinetic and affinity analyses.

Despite the remarkable inhibition of MYC-dependent gene expression in rLon-treated, infected mice, there was no evidence that rLon treatment had a corresponding effect on *Myc* expression in uninfected mice. rLon treatment did not adversely affect healthy mice, indicating that rLon treatment may target the disease response, potentially reducing the risk for off target effects^25^. The molecular basis of this apparent specificity remains unclear, but several scenarios can be proposed. First, the bacterial rLon protease may target disease-specific modifications of MYC that optimize rLon binding and the degradation of its target^13,48–50^. Second, MYC overexpression may make it more available for the protease to execute its proteolytic effect^48^. Third, access of rLon, specifically the NTD and AAA + domains, to MYC promoters may be enhanced in the open chromatin of rapidly dividing cells in cancer or infection, possibly accounting for the pronounced transcriptional inhibition detected in diseased tissues but not in healthy tissues^49,50^. The potent effect of bacterial Lon on mammalian cells and tissues, especially targeting MYC/MAX dimerization, suggests coevolution of the microbiome with mammalian host. Further analysis of Lon proteases from different species would be of interest to understand the structural and functional diversification of Lon from bacteria to higher organisms.

## Methods

### Expression and purification of recombinant Lon protease

The nucleotide sequences of different domains of Lon protease, including the N-terminal domain (NTD), the ATPase domain (AAA+), the peptidase domain (Pep.), as well as the Lon full-length protein, were chemically synthesized (GenScript). These genes were cloned and tagged with Strep-tag II using pASK-63 A + vectors (IBA), carrying the β-lactamase gene resulting in ampicillin resistance as a selectable marker, along with a tetracycline promoter/operator system that tightly controls their expression. Then, four different pASK-63 A + expression constructs containing different Lon protease domains were individually transformed into competent *E. coli* BL21 (DE3) competent cells using the heat-shock method. Selected single colonies were then inoculated in Luria-Bertani (LB) media containing 100 mg/mL ampicillin and incubated at 37 °C with shaking at 200 rpm until the optical density at 600 nm (OD600) reached about 0.5. The cell pellet was then collected by centrifugation at 14,000 x g for 10 min, and the resulting pellets were disrupted by lysis buffer (50 mM Tris pH 7.4, 100 mM NaCl, with or without the addition of protease inhibitors, cOmplete Miniprotease Inhibitor Cocktail, Roche, Cat#04693159001) by sonication on ice (20 s bursts with a 40 s pause between each burst, approximately 10 min), followed by centrifugation (46,000 x g, 30 min, 4 °C). Purification was performed using the poly-prep chromatography column (Bio-Rad) with a diameter of 0.8 cm packed with Strep-Tactin Sepharose (IBA Lifesciences), according to manufacturer’s instructions. The Lon protease was then collected from the column by using elution buffer (50 mM Tris pH 7.4, 100 mM NaCl, 5 mM desthiobiotin, pH 7.4) and dialyzed with the Snakeskin Pleated Dialysis Tubing (10,000 MWCO) against the buffer containing 50 mM Tris pH 7.4, 100 mM NaCl, 10% glycerol by slow shaking at room temperature for at least one hour followed by overnight at 4 °C. The eluted fractions were used either immediately or stored at − 80 °C.

### *In silico* prediction of MYC or MAX-Lon interactions

The Lon sequence (Uniprot ID: P0A9M0) used in the AlphaFold multimer protocol^54^ was identical to that used for recombinantly produced globular domains. The MYC and MAX sequences used were as follows: UniProt ID: P01106-1 (c-MYC), P04198 (N-MYC), P12524-1 (L-MYC) and P61244 (MAX). These sequences used were either full-length or divided into overlapping fragments to ensure that the flexible nature of MYC proteins would not shield potential binding sites of global Lon domains. Sequences were then submitted to CoLabFold using the AlphaFold2 multimer protocol. For searches using sequence fragments, all predictions were subjected to an initial visual sanity check (PyMOL open source), and complexes were filtered out by either the Lon domain purification Strep-tag or those MYC or MAX termini arising from dividing the sequence. Hits were then sorted according to their respective ipTM scores. Configuration of the AlphaFold protocol is supplied in the Supplementary Data file.

### Surface plasmon resonance analysis

Real time biomolecular interaction analysis was performed by surface plasmon resonance (SPR) technology using a Biacore X100 instrument (Cytiva Life Sciences, Uppsala, Sweden) loaded with a Sensor Chip NTA (Cytiva Life Sciences, Cat# BR100034). All experiments were carried out at 25 °C with a constant flow rate of 30 µL/ minute with 0.1 M HEPES, 1.5 M NaCl, 0.03 M EDTA and 0.5% v/v Surfactant P20 (Cytiva Life Sciences, Cat# BR100826). The cell without nickel loading served as a reference for nonspecific binding of proteins and chemicals. This method follows recommendations from senior scientists specializing in Biacore applications, which advise against using a Ni-charged surface without the His-tagged protein for control experiments. By employing a non-Ni-charged surface as the control, this approach ensures the accurate evaluation of specific interactions while minimizing the risk of non-specific binding, thereby preventing misleading results. Adhering to this protocol aligns with best practices and enhances the reliability of experimental outcomes. Biacore X100 control software was programmed to first prime the instrument with running buffer (0.1 M HEPES, 1.5 M NaCl, 0.03 M EDTA and 0.5% v/v Surfactant P20). NiCl_2_ were flowed into saturate the NTA with nickel ions followed by a 3 mM EDTA wash. The His-tagged ligand was prepared in running buffer at concentration ~ 0.2 µM and injected over the nickel activated sensor surface to capture the His-tagged ligand, full-length c-MYC (CSB-EP015270HU, Cusabio), C-terminal c-MYC (Origene, Cat# TP762404), full-length N-MYC (Signalway, Cat# AP72694) or MAX (Novus Biologicals, Cat# NBP1-44497). Sample was injected over the captured His-tagged ligand with five different dilutions for monitoring the interaction of sample with His-tagged ligand. Finally, the chip was regenerated with injection of 350 mM EDTA. Binding affinities were calculated from rate constants obtained from fitting the data to a 1:1 Langmuir binding model. When fitting SPR data with the Langmuir model, the U-value and chi-square (χ²) value serve as key indicators of fit quality. U-values below 15 denote low correlation, affirming that kinetic constants are well resolved and trustworthy. U-values exceeding 25 signify strong parameter correlation, undermining the reliability of the data. A χ² value less than 2% of the maximum response (R_max_) is indicative of a good fit, whereas a χ² exceeding 10% of R_max_ suggests a poor fit. Consequently, K_D_ values derived from fits where χ² remained over 10% of R_max_ were marked with an asterisk.

### DNA-binding ELISA

The TransAM c-MYC Kit (Active Motif, Cat# 43396) was used to assess whether c-MYC activation was inhibited by rLon. Recombinant c-MYC/MAX complex (Active Motif, Cat# 81087) was used as a standard. Recombinant Lon (rLon) and individual domains (690 pmol or 50 ng) were added to 5 ng c-MYC/MAX complex, and DNA-bound MYC was quantified according to the manufacturer’s instructions.

### Cell culture

A498 human kidney carcinoma cells (ATCC, HTB-44, RRID: CVCL_1056), HTB9 human bladder carcinoma cells (ATCC, HTB-9, RRID: CVCL_0126) and A549 human lung carcinoma cells (CCL-185, RRID: CVCL_0023) were cultured in RPMI-1640 supplemented with 1 mM sodium pyruvate, 1 mM non-essential amino acids and 5–10% fetal bovine serum (FBS) overnight at 37 °C with 90% humidity and 5% CO_2_. Cells were treated in fresh, serum-free supplemented RPMI-1640 medium.

### *In vitro* degradation assay by dot blot

Recombinant c-MYC (Cusabio, Cat# CSB-EP015270HU) protein (0.8 μM) was incubated with 0.9 µM, 1.8 µM or 3.6 µM of rLon (produced with or without protease inhibitors) in a reaction buffer consisting of 20 mM MgCl_2_ in the presence of 4 mM ATP in 50 mM Tris pH 8.0. Proteins were incubated at 37 °C for 120 min and then 3 µl of mixture were spotted using narrow-mouth pipette tips on the nitrocellulose membranes (0.45 μm pore size, Amersham Hybond-ECL, Cat# RPN203D). Membranes were air-dried and then blocked with 5% BSA in PBS-T for 60 min at room temperature, followed by incubation overnight at 4 °C with rabbit anti-c-MYC primary antibody (1:1,000 in BSA/PBS-T, Abcam, Cat# ab32072). After washing with PBS-T (3 × 10 min), the membranes were incubated with goat anti-rabbit IgG horseradish peroxide-conjugated secondary antibodies (1:4,000 in 5% BSA/PBS-T, 1 h at room temperature, Bio-Rad, Cat#1706515). Finally, the membranes were washed with PBS-T (2 × 10 min) and PBS (1 × 10 min) and imaged using enhanced chemiluminescence plus detection reagent (GE Healthcare). Protein signals were quantified by ImageJ.

### *In vitro* degradation assay by Western blot

Recombinant c-MYC (Cusabio, Cat# CSB-EP015270HU) or N-MYC (SAB Signalway Antibody, Cat# AP72694) protein (0.2 µg) was incubated with 0.2–1 µg of rLon protease (produced with or without protease inhibitors), or PBS, in a reaction buffer consisting of 20 mM MgCl_2_ in the presence of 2 mM ATP in 50 mM Tris pH 8.0. Proteins were incubated at 37 °C for 120 min and denatured using 10 mM dithiothreitol (DTT) in NuPAGE LDS Sample Buffer (Thermo Fisher Scientific) at 100 °C for 5 min before immunoblot analysis using a c-MYC (Abcam, Cat# ab32072) or N-MYC (Cell Signaling Technology, Cat# 51705) antibodies.

### Labeling of rLon and the domain peptides

The N-terminal labeling of Lon protease was performed with AZ647 NHS Ester (VectorLabs Fluoroprobes, Cat# 1121-2,) according to manufacturer’s instructions. The free dye was removed using a dialysis cassette (D-Tube™ Dialyzer Midi, Merck-Millipore, Cat# 71506) followed by centrifugation with Amicon centrifugal filter unit (Amicon Ultra-15, Merck-Millipore, Cat# UFC900324). The concentration of the labeled complex was determined using NanoDrop 2000c (Thermo Fisher Scientific).

### Cellular uptake using live-cell imaging

A498 or A549 cells were grown in ibidi chamber slides (1 × 10^5^ cells) pretreated with poly-L-lysine overnight. Cells were exposed to labelled rLon or the domain peptides (902 nM) for different time points, as specified in the figure legend, followed by washing of the labeled protein. The cells were then monitored in real time by laser-scanning confocal microscopy (LSM900 confocal microscope, Carl Zeiss). Nuclei were counterstained with Hoechst 33342. The total cellular uptake for quantified for rLon and domain peptides in cells using Image J.

### Surface reconstructions

3D rendering of the fluorescent images of cellular uptake in A549 cells were done using Imaris software 627 (BitPlane, v.9.9) from the whole volume z-stacks. The nuclear surface was made transparent to visualization the nuclear and peri-nuclear distribution of rLon and the domain peptides.

### Whole-genome transcriptome analysis of kidney cells

Total RNA was extracted from kidney epithelial cells with the RNeasy Mini Kit (Qiagen, Cat# 74104) after disruption in RLT buffer supplemented with β-mercaptoethanol (1%) using QIAshredder (Qiagen, Cat# 79656). Exactly 100 ng of total RNA was amplified and fragmented using the Affymetrix WT PLUS Reagent, followed by hybridization onto Clariom S Human arrays (Affymetrix) for 16 h at 45 °C. Then, the sample was washed, stained, and scanned using the GeneChip System as per manufacturer’s instruction (Affymetrix). Data were normalized using Robust Multi Average implemented in Transcriptome Analysis Console software (v.4.0.1.36, Applied Biosystems, Thermo Fisher Scientific). Relative expression was analyzed by ANOVA using the empirical Bayes method, and genes with an absolute fold change > 1.5 were considered differentially expressed. Heatmap was constructed in GraphPad Prism 10.

### Western blots

Cells were lysed with NP-40 lysis buffer supplemented with protease and phosphatase inhibitors (Roche Diagnostics). For nuclear extract preparation, cell compartments were fractionated using a Qproteome Cell Compartment kit (Qiagen, Cat# 37502). Proteins were separated by SDS-polyacrylamide gel electrophoresis (SDS-PAGE; 4–12% Bis-Tris gels, ThermoFisher Scientific) and blotted onto polyvinylidene difluoride membranes (Bio-Rad), which were then blocked and stained with primary rabbit anti-Lon (1:2,000 in 5% BSA/PBS, Biorbyt, Cat# orb231326), rabbit anti-c-MYC (1:1,000 in 5% BSA/PBS-T, Abcam, Cat# ab32072), rabbit anti-L-MYC (Aviva Systems Biology, Cat# ARP35770,) or rabbit anti-N-MYC (Cell Signaling Technology, Cat# 51705) antibodies overnight at 4 °C. Membranes were then washed, stained with goat anti-rabbit IgG horseradish peroxide-conjugated secondary antibodies (1:4,000 in 5% BSA/PBS-T, one hour at room temperature, Bio-Rad, Cat#1706515), and washed again, imaged using enhanced chemiluminescence plus detection reagent (GE Healthcare), and protein signals were quantified by ImageJ. β-Actin (1:4,000 in PBS-T, one hour at room temperature, Sigma, Cat# A5316), GAPDH (1:4,000 in PBS-T, one hour at room temperature, Santa Cruz Biotechnology, Cat# sc-25778) or histone H3 (1:4,000 in PBS-T, one hour at room temperature, Abcam, Cat# ab1791) protein was used as a loading control.

### Immunofluorescence

Cells were washed with PBS, fixed with 2% paraformaldehyde (PFA) for 10 min at room temperature and permeabilized with 0.25% Triton X100 and 5% FBS/PBS for 20 min at room temperature. Cells were then stained with primary rabbit anti-c-MYC (1:200 in 5% FBS/PBS, Abcam, Cat# ab32072), rabbit anti-N-MYC (1:200 in 5% FBS/PBS, Cell Signaling Technology, Cat# 51705) or rabbit anti-L-MYC (1:100 in 5% FBS/PBS, Aviva Systems Biology, Cat# ARP35770,) antibodies overnight at 4 °C. Subsequently, the cells were washed and stained with secondary goat anti-rabbit IgG Alexa Fluor 488-conjugated antibody (1:200 in 5% FBS/PBS, Thermo Fisher Scientific, Cat# A-11034) for one hour at room temperature. Cells were counterstained with Hoechst 33,342 (1:2,000 in PBS, Molecular Probes, Cat# H1399) nucleic acid stain for 10 min at room temperature and imaged by confocal microscopy. Quantification of fluorescence signal intensity was measured in 50 cells per condition using ImageJ.

### Cell death assays

Cells (4 × 10^4^ cells per well) were seeded in serum-free RPMI-1640 on 96-well plates (Tecan Group Ltd). Cells were treated with rLon protease or different domain peptides (902 and 2700 nM) and incubated for three hours. Cell viability or death was quantified by two different assays. The released ATP was determined using the luminescence-based ATPlite kit (Perkin Elmer, Cat#6016947) and mitochondrial enzyme activity was determined by the PrestoBlue Cell viability assay (Thermo Fisher Scientific, Cat# A13262). Assays were done in triplicate, and mean values were calculated.

### Bacterial strains

The prototype APN strain *E. coli*CFT073 (O6:K2:H1)^55^was cultured on tryptic soy agar (TSA) plates at 37 °C for 16 h, harvested in phosphate-buffered saline (PBS, pH 7.2) and diluted as appropriate^26^.

To quantify effects of Lon on bacterial viability, *E. coli* CFT073 bacteria (CFU = 10^4^) were first cultured in 1,000 µl Luria Broth (LB) media for 3.5 h at 37 °C after which rLon (250–500 µg/mL) or cefotaxime (100 mg/mL) was added to the bacterial culture. The OD600 (optical density at 600 nm) was measured at different timepoints (0, 1, 2, 3, 4 and 5 h).

### Experimental kidney infection model

*Irf3*^−/−^mice on a C57BL/6 background were kindly provided by the Riken Bioresource Center, Japan, with permission from T. Taniguchi^56^. Mice were bred at Lund University, BMC animal facility, Lund, Sweden, and housed in specific pathogen-free individually ventilated cages (IVCs) at a constant temperature of 23 °C on a 12-hour light-dark cycle with lights on at 7:00 a.m. and *ad libitum* access to food and water. Female *Irf3*^−/−^ mice were used for experiments at 9–15 weeks of age.

Mice were anesthetized by intraperitoneal (*i.p.*) injection of a cocktail of ketamine (1.48 mg in 100 µL of 0.9% NaCl solution, Intervet) and xylazine (0.22 mg in 100 µL of 0.9% NaCl solution, Vetmedic) and infected by intravesical inoculation with *E. coli* CFT073 (10^8^CFU in 0.05 mL)^57^ through a soft polyethylene catheter (2 × 10^9^ CFU/mL, 50 µL). Urine samples were obtained from each mouse by holding the animal over a sterile 1.5 mL Eppendorf tube before infection and at regular times after infection (24 h, three days, five days and seven days). The urine drops were immediately collected, viable bacteria counts were determined by growth on TSA plates (37 °C, overnight) after serial dilutions as appropriate. Neutrophils in uncentrifuged urine were counted using a hemocytometer chamber. The mice were sacrificed by using an overdose of isoflurane anesthesia (Dechra, Cat# 200 − 129) after 24 h and seven days. Organs were aseptically removed, and macroscopic pathology was documented by photography. Tissue samples were fixed for 24 h in 4% paraformaldehyde (PFA) at 4 °C and embedded in paraffin for hematoxylin and eosin (H&E) and immunohistochemistry analysis. The degrees of hyperemia, edema and abscesses were scored on a scale from 0 to 10, where 0 was similar to that of uninfected controls, and 10 indicated the most severe hyperemia, edema and abscesses. One kidney per mouse was divided for RNA extraction (on dry ice) and immunostaining (in O.C.T. compound or 10% (v/v) formalin).

### Recombinant lon protease treatment in mice

Recombinant bacterial Lon protease was administered intraperitoneally (rLon, 100 µL, 250 µg/mL) to *Irf3*^*−/−*^ mice once a day for eight days starting one day before intravesical infection with *E. coli* CFT073 (day 0). Placebo-treated mice received intraperitoneally 100 µl of PBS with the same schedule as rLon-treated mice. The mice were sacrificed by using an overdose of isoflurane anesthesia (Dechra, Cat# 200 − 129) after 24 h and seven days.

*Apc*^*Min/+*^ (C57BL/6J-*Apc*^*Min*^/J, RRID: IMSR JAX002020) mice and C57BL/6 mice were obtained from Jackson Laboratories at approximately eight weeks of age. *Apc*^*Min/+*^ mice carry a heterozygous mutation in the murine *Apc* (*adenomatous polyposis coli*) locus, which consequently encodes a nonsense mutation at codon 850 resulting in multiple intestinal neoplasia (*Min*)^36,37^. C57BL/6J-*Apc*^*Min*^/J mice, which develop spontaneous intestinal adenomas^58^, were bred at Lund University and used at 11 weeks old. Mice were treated twice daily for 14 days by oral gavage of rLon protease (200 µL, 250 µg/mL). At sacrifice, intestines were aseptically harvested, and segments of the intestines were prepared for mRNA extraction.

### Transcriptome analysis of mouse tissues

Total RNA was extracted from murine kidneys or intestines with the RNeasy Mini Kit (Qiagen, Cat#74104) after disruption in RLT buffer supplemented with β-mercaptoethanol (1%) using Precellys Lysing kits (Bertin Technologies) and Tissuelyser (Qiagen). For treated cells, RNA was extracted with the RNeasy Mini Kit and QIAshredder (Qiagen, Cat# 79656). Exactly 100 ng of total RNA was amplified and fragmented using the Affymetrix WT PLUS Reagent or GeneChip 3´IVT Express kits, followed by hybridization onto Clariom S Mouse HT or Mouse Genome 430 PM (Affymetrix) for 16 h at 45 °C. Then, the sample was washed, stained using the Affymetrix fluidic station 450 as per manufacturer’s instruction (Hybridization, Washing and Staining kit, Affymetrix). Microarrays were immediately scanned using the GeneTitan or GeneAtlas system (Affymetrix). Data were normalized using Robust Multi Average implemented in Transcriptome Analysis Console software (v.4.0.1.36, Applied Biosystems, Thermo Fisher Scientific). Relative expression was analyzed by ANOVA using the empirical Bayes method, and genes with an absolute fold change ≥ 2.0 were considered differentially expressed. Differentially expressed genes were functionally analyzed using Ingenuity Pathway Analysis software (IPA, Qiagen), and data are shown in heatmaps constructed in GraphPad Prism v.10. Significantly altered genes were sorted by relative expression (*P* < 0.05 and absolute FC ≥ 2.0) and analyzed using GSEA (http://www.broadinstitute.org/gsea/index.jsp).

### Histology and immunohistochemistry

Tissues embedded and frozen in O.C.T. were cryosectioned (8 µm thick sections, Leica microtome), collected on positively charged microscope slides (Superfrost/Plus, Thermo Fisher Scientific), fixed in acetone-methanol (1:1, 10 minutes), permeabilized (0.2% Triton X-100, 5% normal goat serum/PBS) and incubated overnight at 4°C with primary rabbit anti-c-MYC (1:100, Abcam, Cat# ab32072), rabbit anti-L-MYC (1:100, Aviva Systems Biology, Cat# ARP35770) or rabbit anti-N-MYC (1:100, Cell Signaling Technology, Cat# 51705) antibodies, followed by incubation for one hour at room temperature with Alexa 488- or Alexa 568-labeled rabbit anti-rat and goat anti-rabbit IgG secondary antibodies (1:100, Molecular Probes, Cat# A-21210 and Cat# A-11011). Nuclei were counterstained with DAPI (4’,6-diamidino-2-phenylindole, 0.05 mM, Sigma-Aldrich, Cat# D8417) for 15 min at room temperature. Slides were examined by fluorescence microscopy (AX60, Olympus Optical) and fluorescence intensity was quantified by using ImageJ. Richard-Allan Scientific Signature Series Hematoxylin 7211 and Eosin-Y 7111 (H&E) (ThermoFisher Scientific) were used to stain the tissue sections to identify histopathological changes.

### Quantification and statistical analysis

For data that had a Gaussian distribution (D’Agostino & Pearson normality test), significant differences were determined by unpaired t-test, one-way ANOVA or two-way ANOVA with appropriate multiple comparison test. For nonparametric data, either the two-tailed Mann-Whitney U test or Kruskal-Wallis test with Dunn’s correction was used. Statistical significance was determined by GraphPad (Prism v. 10), and significance was assigned at * *P <* 0.05, ** P < 0.01 and *** P < 0.001. All data are presented as the median or mean, and all error bars represent the standard deviation (s.d.) or standard error mean (s.e.m.). Sample size (*n*) indicates either the number of independent experiments or the number of mice used in the study.

### Study approval

The study was approved by the Malmö/Lund Animal Experimental Ethics Committee at the Lund District Court in Sweden (#M119-16 and 6551 − 2021). All methods were performed in accordance with the European Parliament and Council Directive (2016/63, EU), the Swedish Animal Welfare Act (Djurskyddslagen 1988:534), the Swedish Welfare Ordinance (Djurskydssförordningen 1988:539) and Institutional Animal Care and Use Committee (IACUC) Guidelines. The study was conducted in accordance with ARRIVE guidelines (https://arriveguidelines.org*).*

## Electronic supplementary material

Below is the link to the electronic supplementary material.

Supplementary Material 1

## Data Availability

Data that support the findings of this study are available within the manuscript or supplementary information files and from the corresponding author upon reasonable request.
