# Supplementary material for: Multitarget mechanism of MYC inhibition by the bacterial lon protease in disease
Source: Sci Rep. 2025 Feb 25;15:6778. doi: 10.1038/s41598-025-88093-2 (PMC11861601; doi:10.1038/s41598-025-88093-2)
Supplement: Supplementary file 1 — Supplementary Material 1 [file 41598_2025_88093_MOESM1_ESM.pdf]

Extended Data Fig. 1

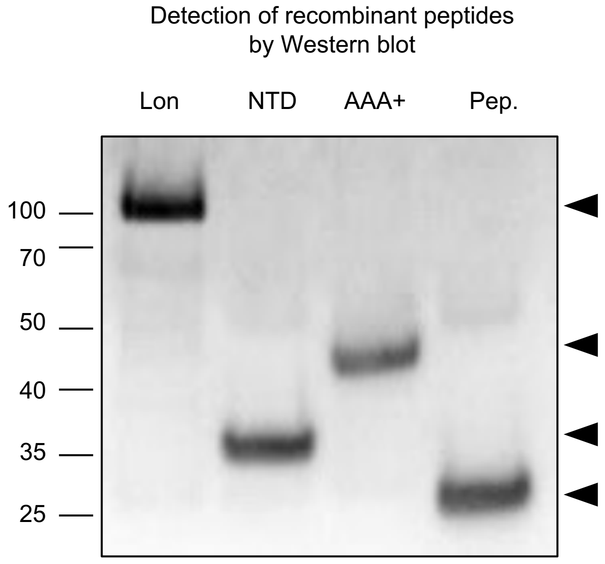

Extended Data Fig. 1. Supplementary Data for purity of recombinant peptides in Main Fig. 1
Detection of the recombinant Lon, N-terminal domain (NTD), AAA+ and peptidase domains, by Western blot.

Extended Data Fig. 2

		Peptidase
**c-MYC**	Full-length	0.324
	NTD	0.231
	DR	0.363
	CTD	0.142
**N-MYC**	Full-length	0.320
	NTD	-
	DR	0.272
	CTD	-
**L-MYC**	Full-length	0.300
	NTD	-
	DR	0.296
	CTD	-

Extended Data Fig. 2. Supplementary Data for AlphaFold analysis in Main Fig. 1
Top ipTM score for prediction of interactions of Lon peptidase domain with full-length c-, N- and L-MYC and their domains. NTD = N-terminal domain; DR = Disordered regions; CTD = C-terminal domain.

Extended Data Fig. 3

a

Predicted interaction between L-MYC (full) and Lon NTD

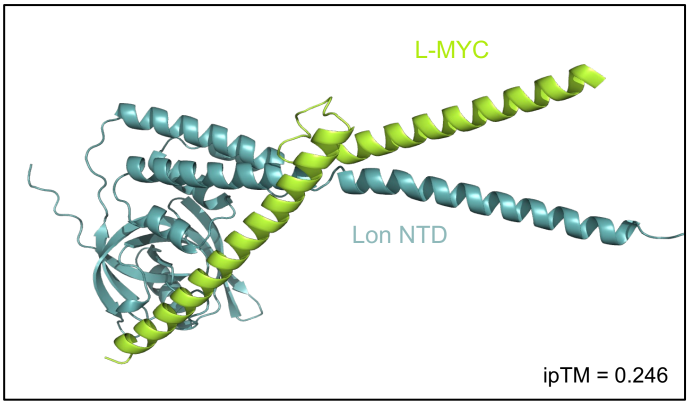

b

Predicted interaction between L-MYC (full) and Lon peptidase

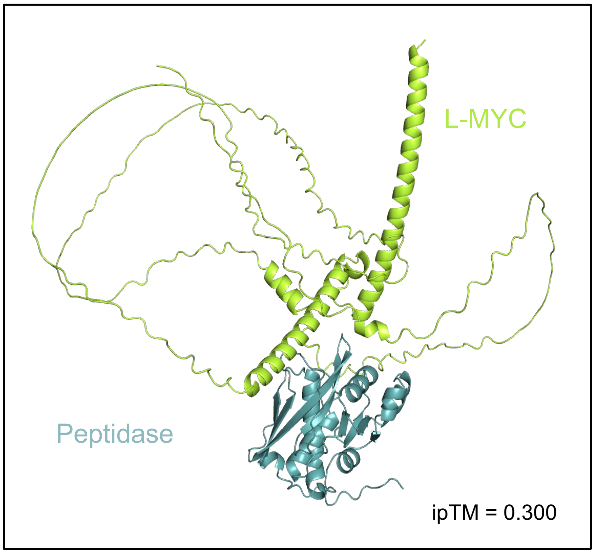

c

Peptidase binding to c-MYC (full)

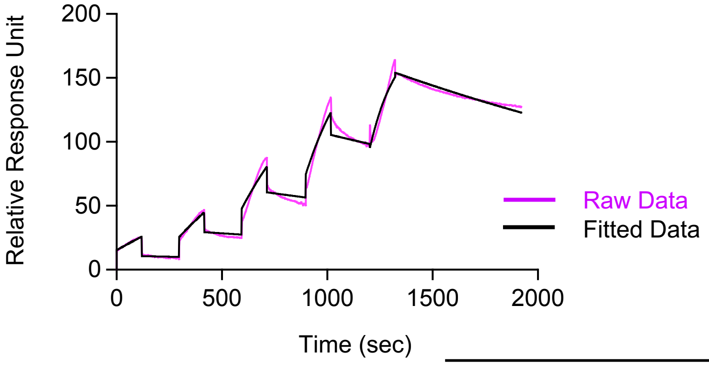

d

AAA+ binding to c-MYC (full)

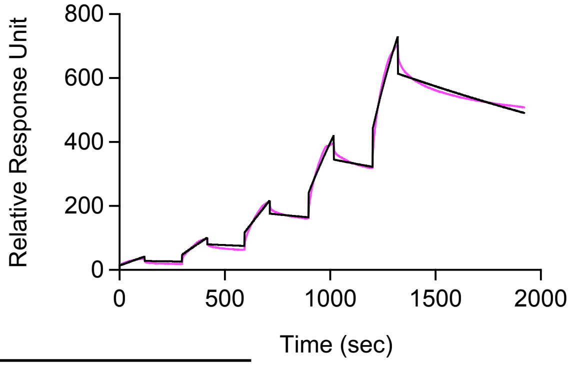

K_D (μM)	Peptidase	AAA+
**c-MYC (full)**	0.849	0.534

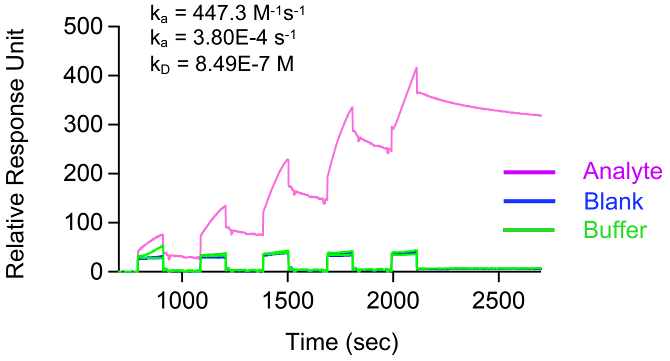

R_{max} (RU)	Conc. (M)	tc	Flow (μl·min-1)	k_t (RU·M-1s-1)	RI (RU)	Chi² (RU²)	U-value
197.5		1.836E+6				15.9	3
	1.08E-6		30	5.7E+6	15.25		
	2.16E-6		30	5.7E+6	15.59		
	4.33E-6		30	5.7E+6	20.23		
	8.65E-6		30	5.7E+6	17.98		
	1.73E-5		30	5.7E+6	-3.61		

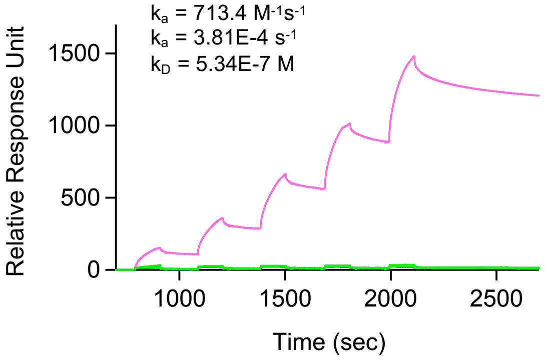

R_{max} (RU)	Conc. (M)	tc	Flow (μl·min-1)	k_t (RU·M-1s-1)	RI (RU)	Chi² (RU²)	U-value
1414		8.67E+06				138	2
	2.5E-7		30	2.7E+7	13.74		
	5.0E-7		30	2.7E+7	21.08		
	1.0E-6		30	2.7E+7	41.38		
	2.0E-6		30	2.7E+7	76.93		
	4.0E-6		30	2.7E+7	118.2		

Extended Data Fig. 3. Supplementary Data for AlphaFold and SPR analysis in Main Fig. 1
a, **b**, AlphaFold predicted interactions between full-length L-MYC and NTD and peptidase with a low ipTM score of 0.246 and 0.300. **c, d,** The SPR binding analysis shows the dose-dependent interaction of peptidase and AAA+ peptide to His-tagged c-MYC captured on Sensor Chip NTA (magenta). The data was fitted to a 1:1 Langmuir binding model (black). The table shows the dissociation constant (K_D) of bindings.

Extended Data Fig. 4

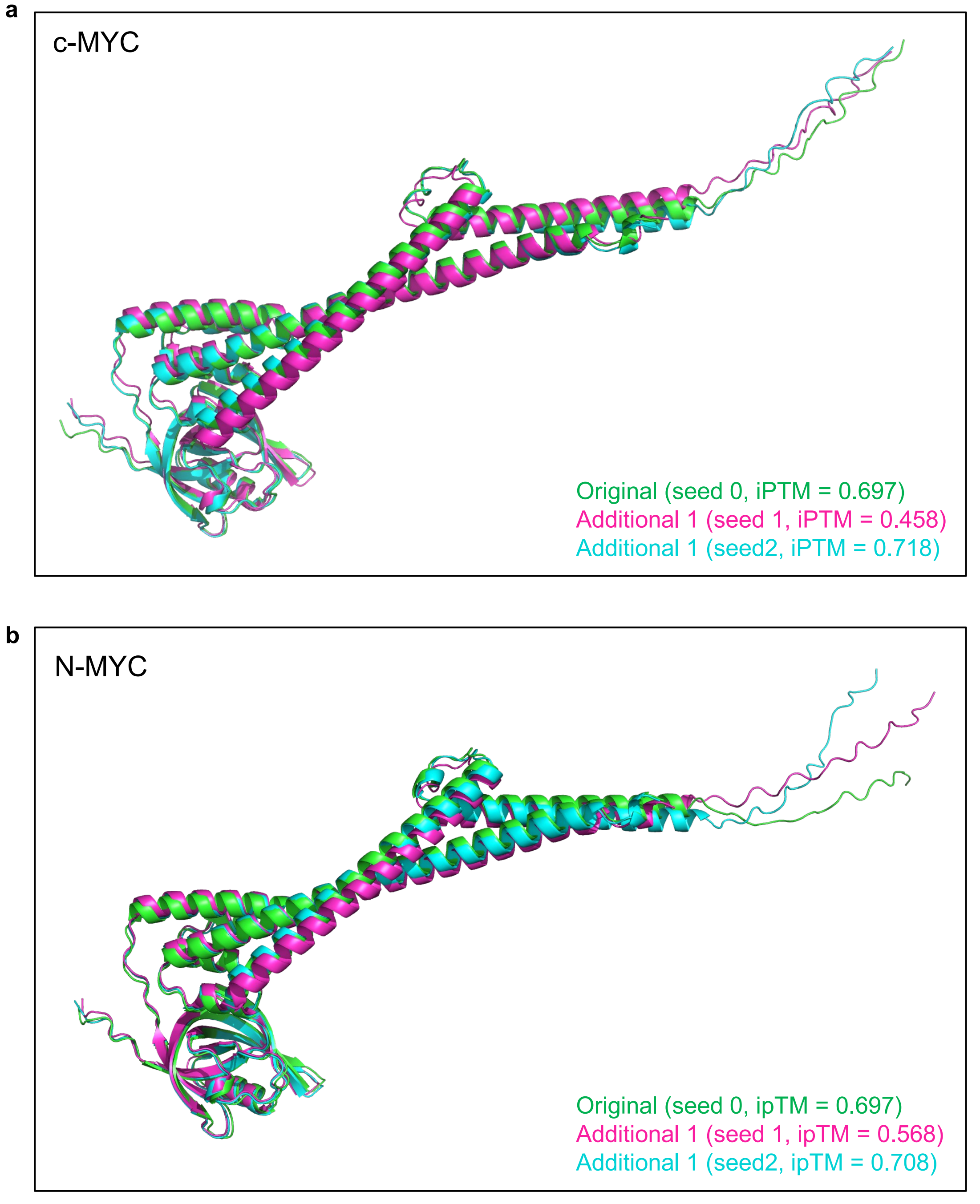

Extended Data Fig. 4. Additional seeds for AlphaFold analysis in Main Fig. 1
a, Overlay of the best scoring model of the c-MYC complex with NTD, and its two additional seeds. **b,** Overlay of the best scoring model of the N-MYC complex with NTD, and its two additional seeds.

Extended Data Fig. 5

a NTD binding to c-MYC (full)

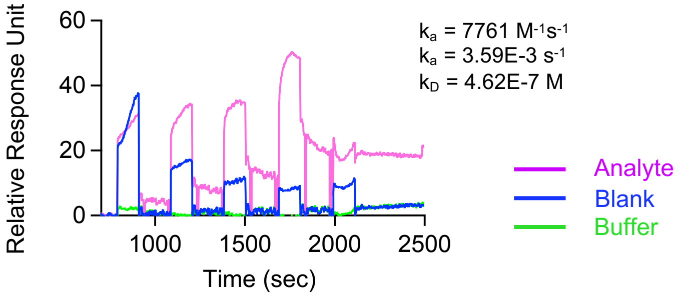

R_{max} (RU)	Conc. (M)	tc	Flow (μl·min⁻¹)	k_t (RU·M⁻¹s⁻¹)	RI (RU)	Chi² (RU²)	U-value
209.4		1.59E+14				19.8	2
	1.5E-7		30	4.93E+14	9.03		
	3.0E-7		30	4.93E+14	14.81		
	6.0E-7		30	4.93E+14	28.22		
	1.2E-6		30	4.93E+14	48.26		
	2.4E-6		30	4.93E+14	33.59		

b Lon binding to c-MYC (full)

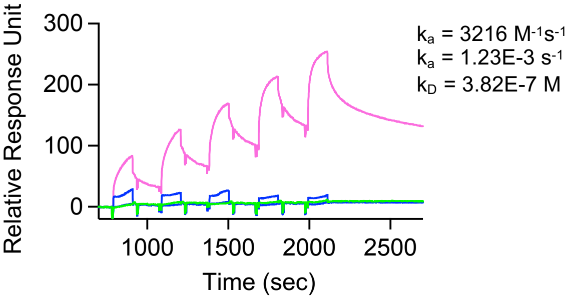

R_{max} (RU)	Conc. (M)	tc	Flow (μl·min⁻¹)	k_t (RU·M⁻¹s⁻¹)	RI (RU)	Chi² (RU²)	U-value
872.8		3.14E+7				203	5
	5.25E-8		30	9.76E+7	4.43		
	1.05E-7		30	9.76E+7	21.49		
	2.10E-7		30	9.76E+7	35.72		
	4.20E-7		30	9.76E+7	61.36		
	8.40E-7		30	9.76E+7	94.86		

c NTD binding to N-MYC (full)

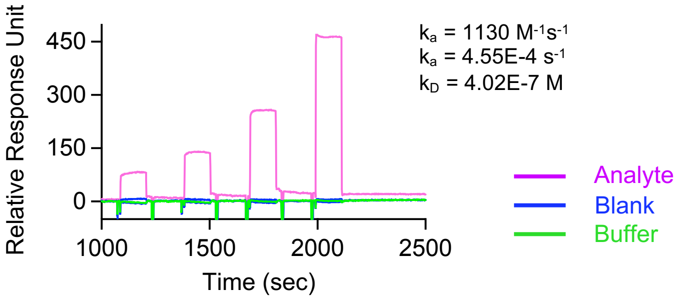

R_{max} (RU)	Conc. (M)	tc	Flow (μl·min⁻¹)	k_t (RU·M⁻¹s⁻¹)	RI (RU)	Chi² (RU²)	U-value
187.3		4.27E+5				10.9	2
	2.45E-7		30	1.33E+6	11.76		
	4.90E-7		30	1.33E+6	14.81		
	9.79E-7		30	1.33E+6	27.55		
	1.96E-6		30	1.33E+6	28.82		
	3.92E-6		30	1.33E+6	31.90		

d Lon binding to N-MYC (full)

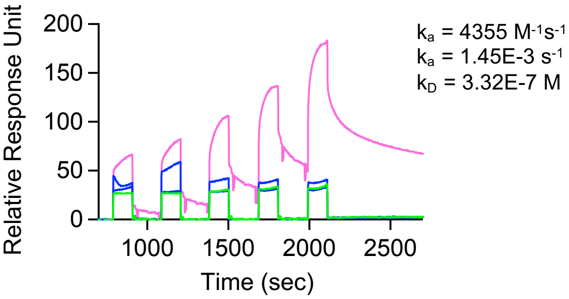

R_{max} (RU)	Conc. (M)	tc	Flow (μl·min⁻¹)	k_t (RU·M⁻¹s⁻¹)	RI (RU)	Chi² (RU²)	U-value
616.9		2.198E+7				130	3
	5.63E-8		30	6.83E+7	13.23		
	1.13E-7		30	6.83E+7	20.20		
	2.25E-7		30	6.83E+7	35.89		
	4.50E-7		30	6.83E+7	63.95		
	9.00E-7		30	6.83E+7	104.1		

e NTD binding to c-MYC (CTD)

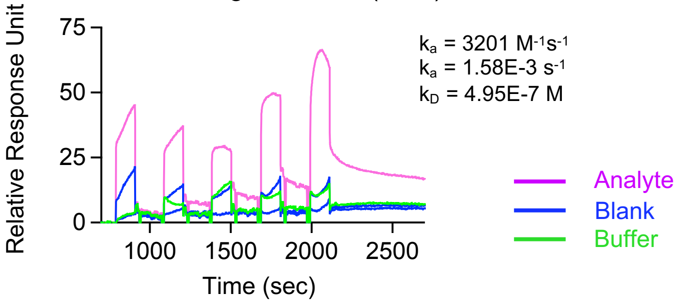

R_{max} (RU)	Conc. (M)	tc	Flow (μl·min⁻¹)	k_t (RU·M⁻¹s⁻¹)	RI (RU)	Chi² (RU²)	U-value
417.3		1.67E+6				268	5
	2.45E-7		30	5.20E+6	14.05		
	4.90E-7		30	5.20E+6	39.17		
	9.79E-7		30	5.20E+6	57.11		
	1.96E-6		30	5.20E+6	86.55		
	3.92E-6		30	5.20E+6	138.1		

f Lon binding to c-MYC (CTD)

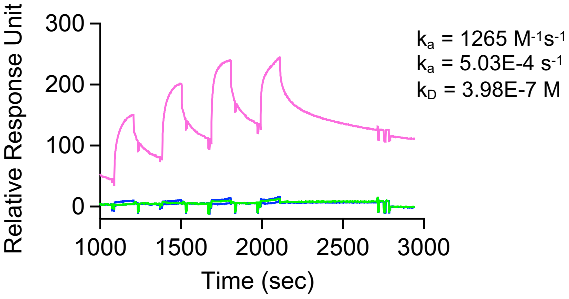

R_{max} (RU)	Conc. (M)	tc	Flow (μl·min⁻¹)	k_t (RU·M⁻¹s⁻¹)	RI (RU)	Chi² (RU²)	U-value
716.4		2.80E+16				379	2
	5.45E-7		30	8.71E+16	145.3		
	1.09E-6		30	8.71E+16	44.28		
	2.18E-6		30	8.71E+16	62.47		
	4.36E-6		30	8.71E+16	73.81		
	8.72E-6		30	8.71E+16	57.53		

Extended Data Fig. 5. Supplementary Data for SPR analysis in Main Fig. 1.
a-f, Raw SPR data obtained from the reference surface (Fc 1) during the binding assay to c-MYC and N-MYC using the NTA sensor chip. This display captures the running buffer (green) and sample buffer responses (referred as blank, blue), commonly referred to as "bulk responses“, to ensure reliable baseline corrections. Analyte-specific curves (magenta) highlight the functional differences among the proteins studied. Binding affinities were calculated from rate constants obtained from data fitting to a 1:1 Langmuir binding model. Parameters derived from the fitting process are presented in each table. The U- and chi-square (χ^2) values serve as a key indicators of fit quality to the Langmuir model. All analyzed U-values were below 15, confirming the robustness of the fitting process.

Extended Data Fig. 6

a

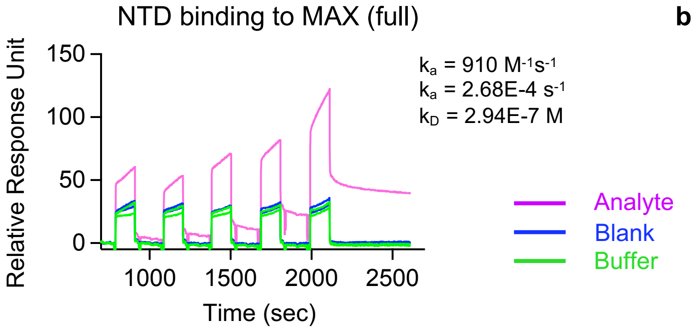

R_{max} (RU)	Conc. (M)	tc	Flow (µl·min-1)	k_t (RU·M-1s-1)	RI (RU)	Chi² (RU²)	U-value
61.41		1.01E+17				1.99	4
	3.13E-7		30	3.14E+17	22.53		
	6.25E-7		30	3.14E+17	15.75		
	1.25E-6		30	3.14E+17	28.08		
	2.50E-6		30	3.14E+17	25.14		
	5.00E-6		30	3.14E+17	38.07		

b

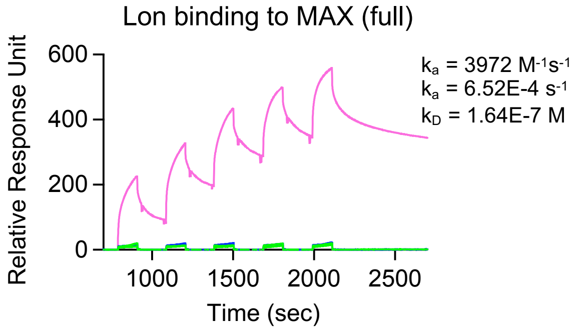

R_{max} (RU)	Conc. (M)	tc	Flow (µl·min-1)	k_t (RU·M-1s-1)	RI (RU)	Chi² (RU²)	U-value
363.8		5.88E+18				129	2
	3.13E-7		30	1.83E+19	43.38		
	6.25E-7		30	1.83E+19	49.69		
	1.25E-6		30	1.83E+19	69.23		
	2.50E-6		30	1.83E+19	65.57		
	5.00E-6		30	1.83E+19	73.25		

c

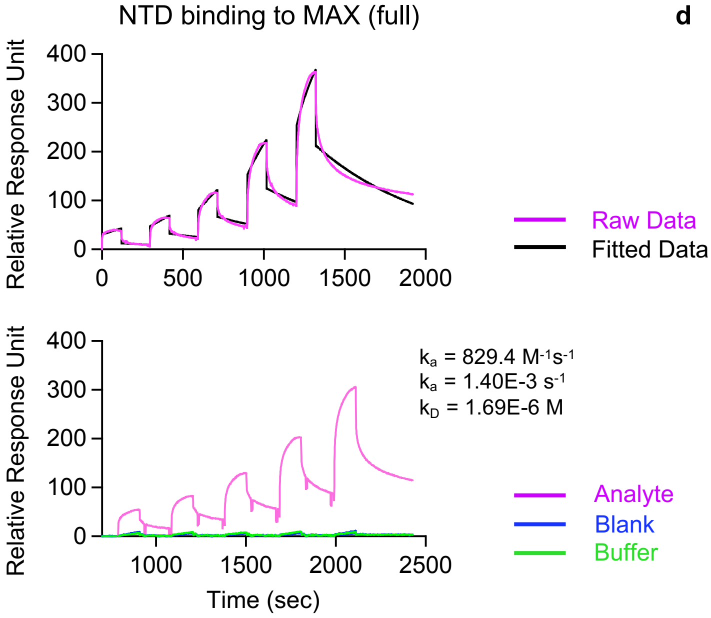

R_{max} (RU)	Conc. (M)	tc	Flow (µl·min-1)	k_t (RU·M-1s-1)	RI (RU)	Chi² (RU²)	U-value
453.3		3.53E+6				137	4
	3.13E-7		30	1.10E+7	30.51		
	6.25E-7		30	1.10E+7	36.93		
	1.25E-6		30	1.10E+7	54.81		
	2.50E-6		30	1.10E+7	100.3		
	5.00E-6		30	1.10E+7	156.2		

d

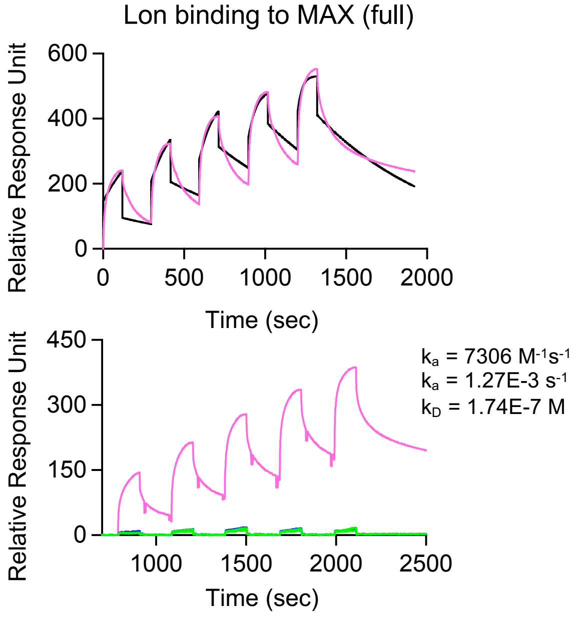

R_{max} (RU)	Conc. (M)	tc	Flow (µl·min-1)	k_t (RU·M-1s-1)	RI (RU)	Chi² (RU²)	U-value
426.6		8.73E+20				976	3
	3.13E-7		30	2.71E+21	145.5		
	6.25E-7		30	2.71E+21	131.9		
	1.25E-6		30	2.71E+21	109.9		
	2.50E-6		30	2.71E+21	94.4		
	5.00E-6		30	2.71E+21	119.4		

Extended Data Fig. 6. Supplementary Data for SPR analysis in Main Fig. 2.
a-d, Raw SPR data obtained from the reference surface (Fc 1) during the binding assay to MAX using the NTA sensor chip. This display captures the running buffer (green) and sample buffer responses (referred as blank, blue), commonly referred to as "bulk responses“, to ensure reliable baseline corrections. Analyte-specific curves (magenta) highlight the functional differences among the proteins studied. Binding affinities were calculated from rate constants obtained from data fitting to a 1:1 Langmuir binding model. Parameters derived from the fitting process are presented in each table. The U- and chi-square (χ^2) values serve as a key indicators of fit quality to the Langmuir model. All analyzed U-values were below 15, confirming the robustness of the fitting process.

Extended Data Fig. 7

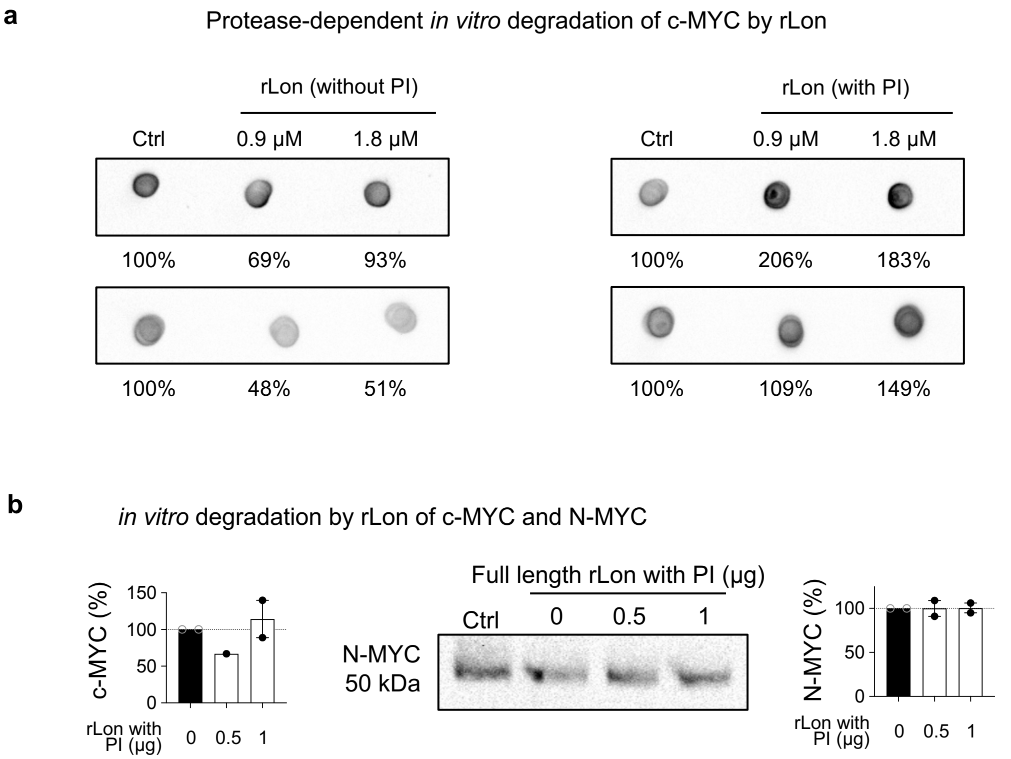

Extended Data Fig. 7. Supplementary Data for *in vitro* degradation experiment for Main Fig. 2.
a, Dot blot analysis showing the degradation of c-MYC (24 picomoles) by rLon (0.9 or 1.8 μM) after 120 minutes. **b**, c-MYC and N-MYC (50 ng) were degraded by rLon (0.5 or 1 μg) after 120 minutes (See Main Fig. 2). This effect was reversed in the presence of protease inhibitors. Data are presented as the means ±s.e.m, mixed-effect analysis with Holm-Sidak's multiple comparisons test (*n* = 2 experiments).

Extended Data Fig. 8

a

in vitro degradation of c-MYC by Western blots

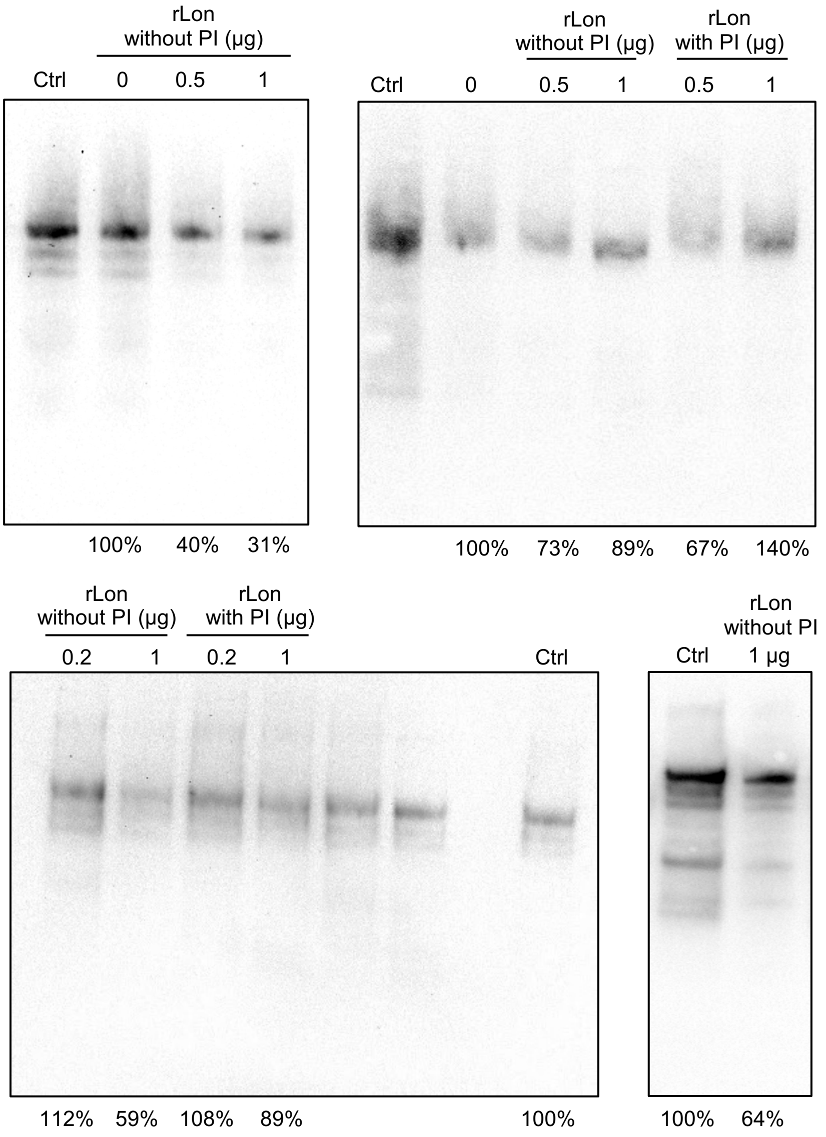

Extended Data Fig. 8

b *in vitro* degradation of N-MYC by Western blots

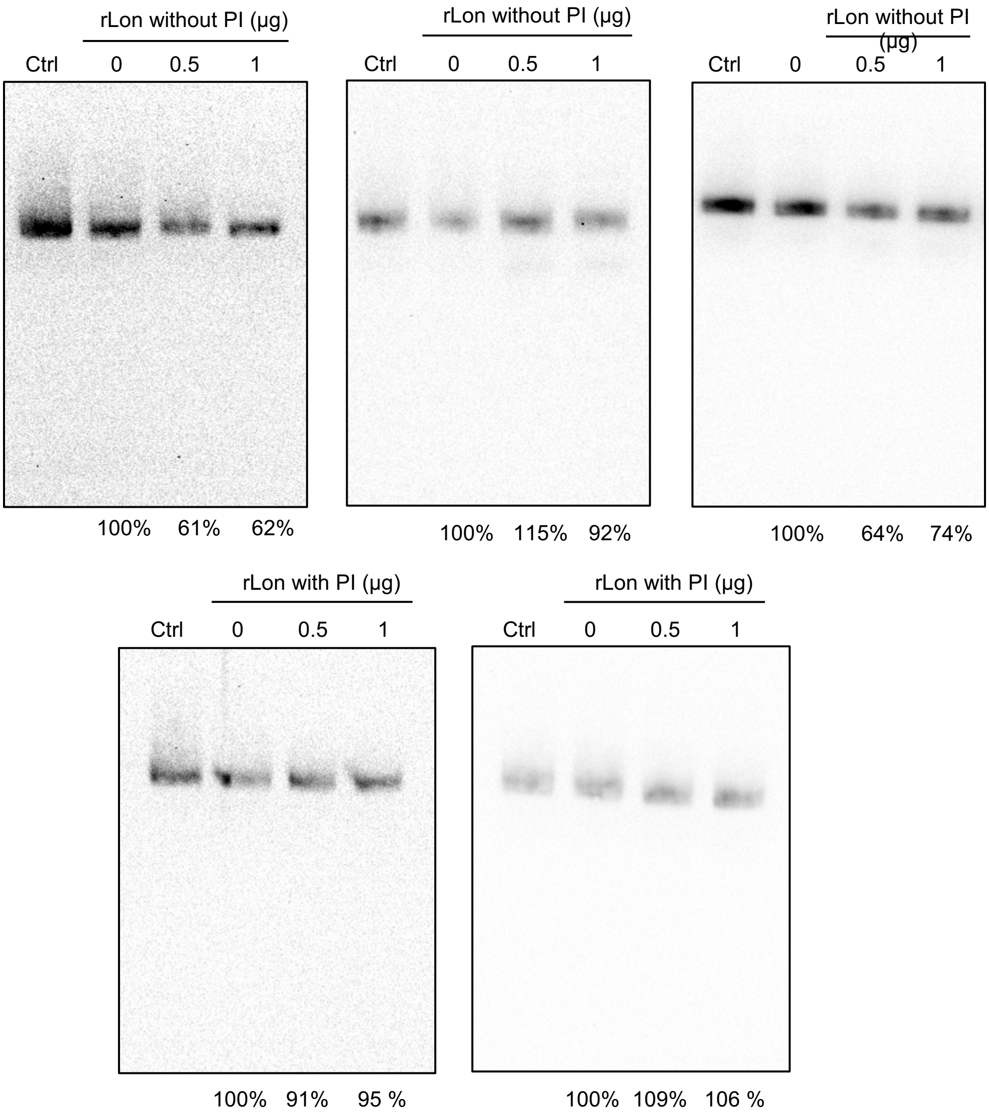

Extended Data Fig. 8. Supplementary Data for *in vitro* degradation experiment for Main Fig. 2. Full-length Western blot showing the repeat experiments of the *in vitro* degradation of c-MYC and N-MYC by rLon.

Extended Data Fig. 9

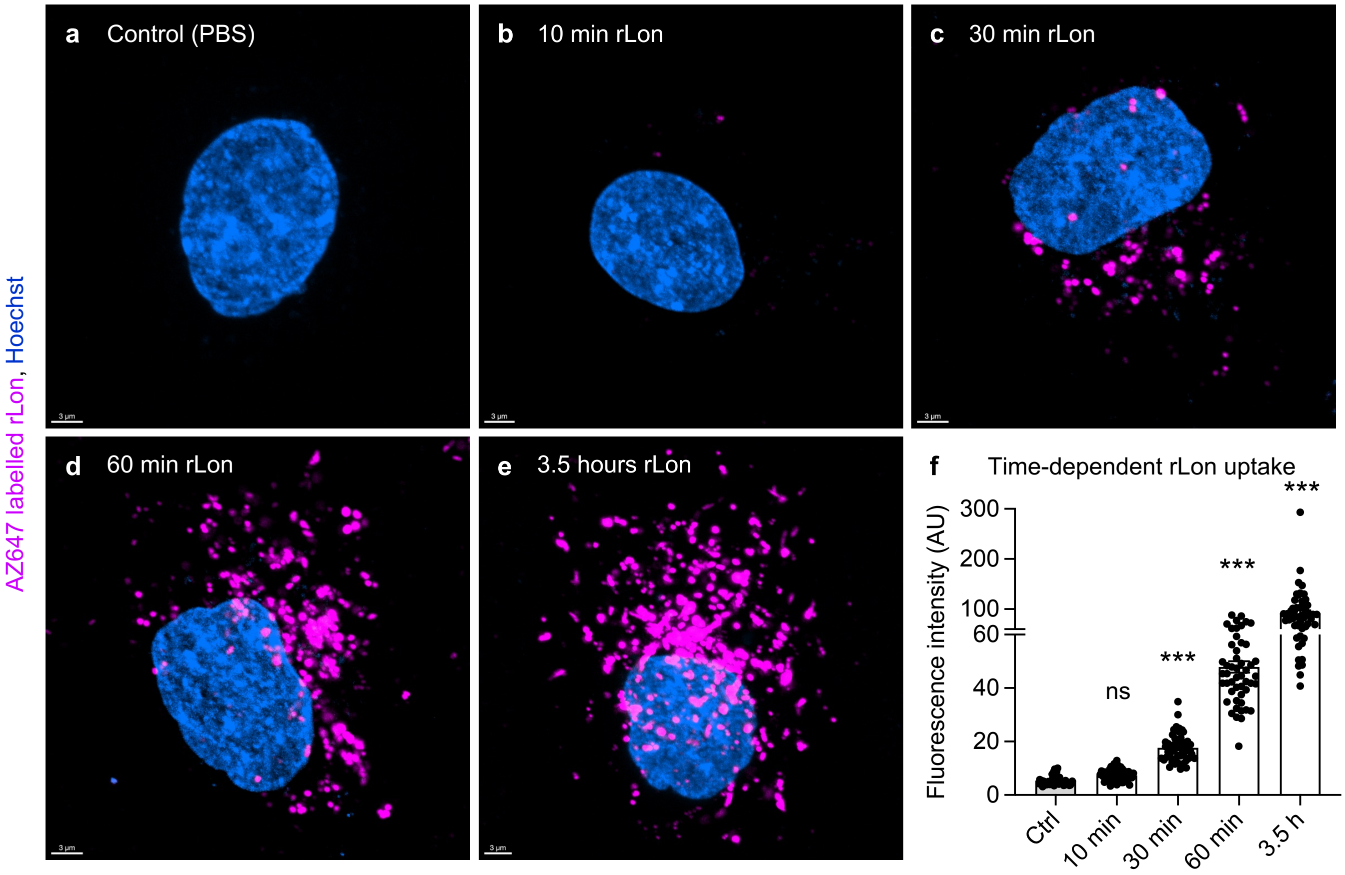

Extended Data Fig. 9. Supplementary Data for rLon uptake for Main Fig. 3.
a-e, Live-cell confocal images showing time-dependent uptake of AZ647 labelled rLon in A498 cells. Magenta, rLon; blue, nuclei; scale bars, 3 μm. **f**, Quantification of the uptake data from (**a-e**). Data are presented as the means ±s.e.m.., Kruskal-Wallis followed by Dunn's multiple comparisons (n = 50 cells). ***$P < 0.001$, compared to control. n.s. = not significant.

Extended Data Fig. 10

a

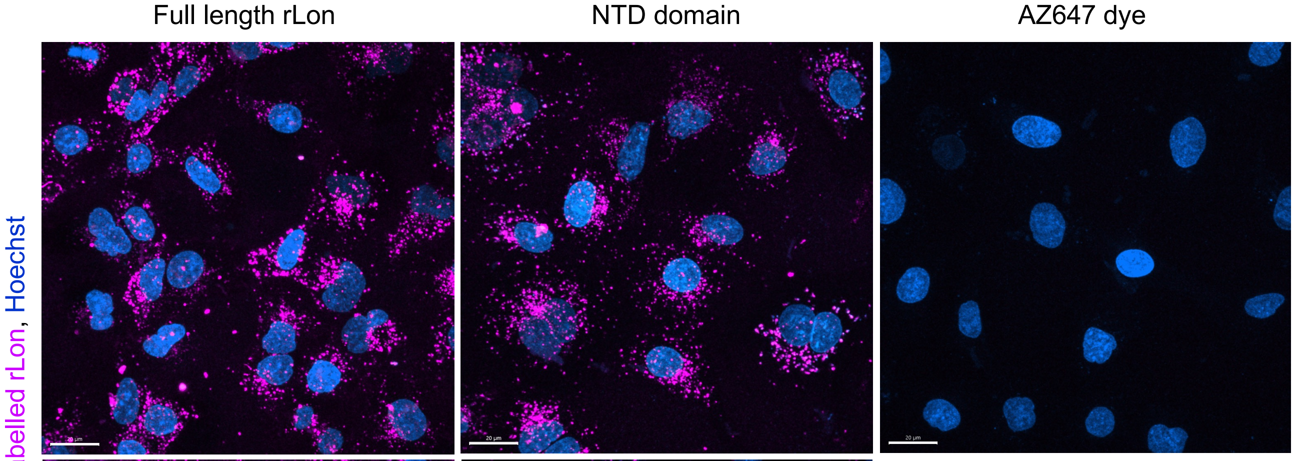

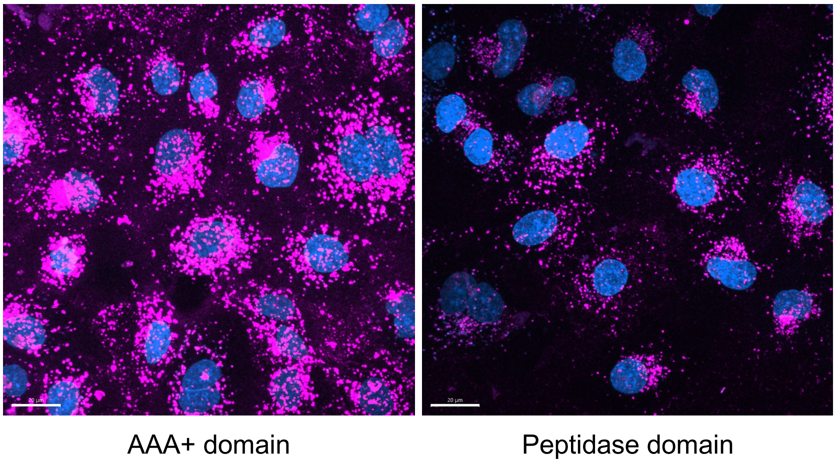

Degree of labelling (%)	
**rLon**	58.2
**NTD**	54.8
**AAA+**	68.6
**Peptidase**	62.7

b

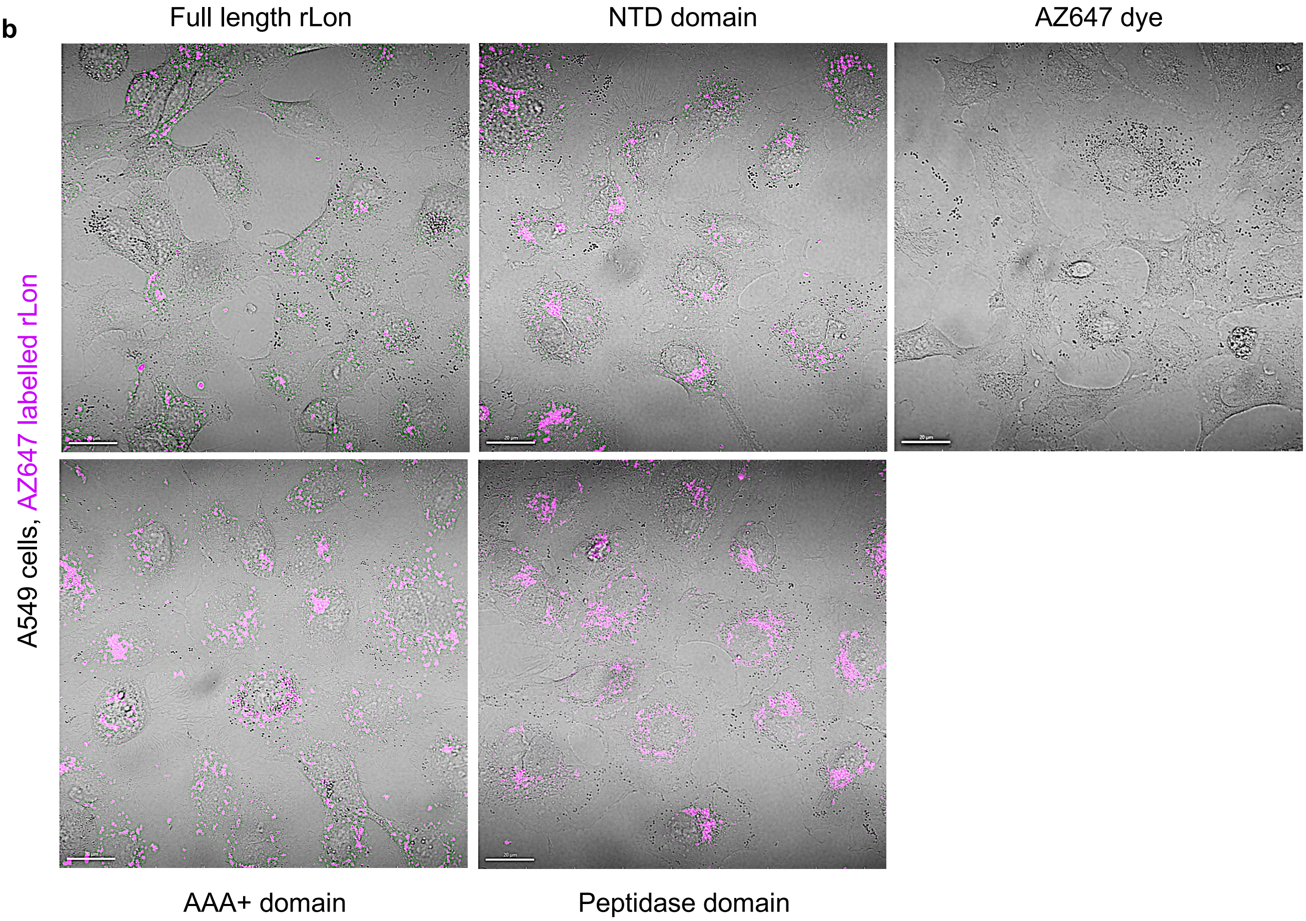

Extended Data Fig. 10. Supplementary Data for Main Fig. 3.
a, Confocal images showing the uptake of AZ647 labelled rLon (902 nM) and Lon peptide (NTD, AAA+ and peptidase domains, 902 nM) in A549 cells. Degree of AZ647 labelling of peptide is shown in the table. **b**, Brightfield images. Magenta, rLon; blue, nuclei; scale bars, 20 μm.

Extended Data Fig. 11

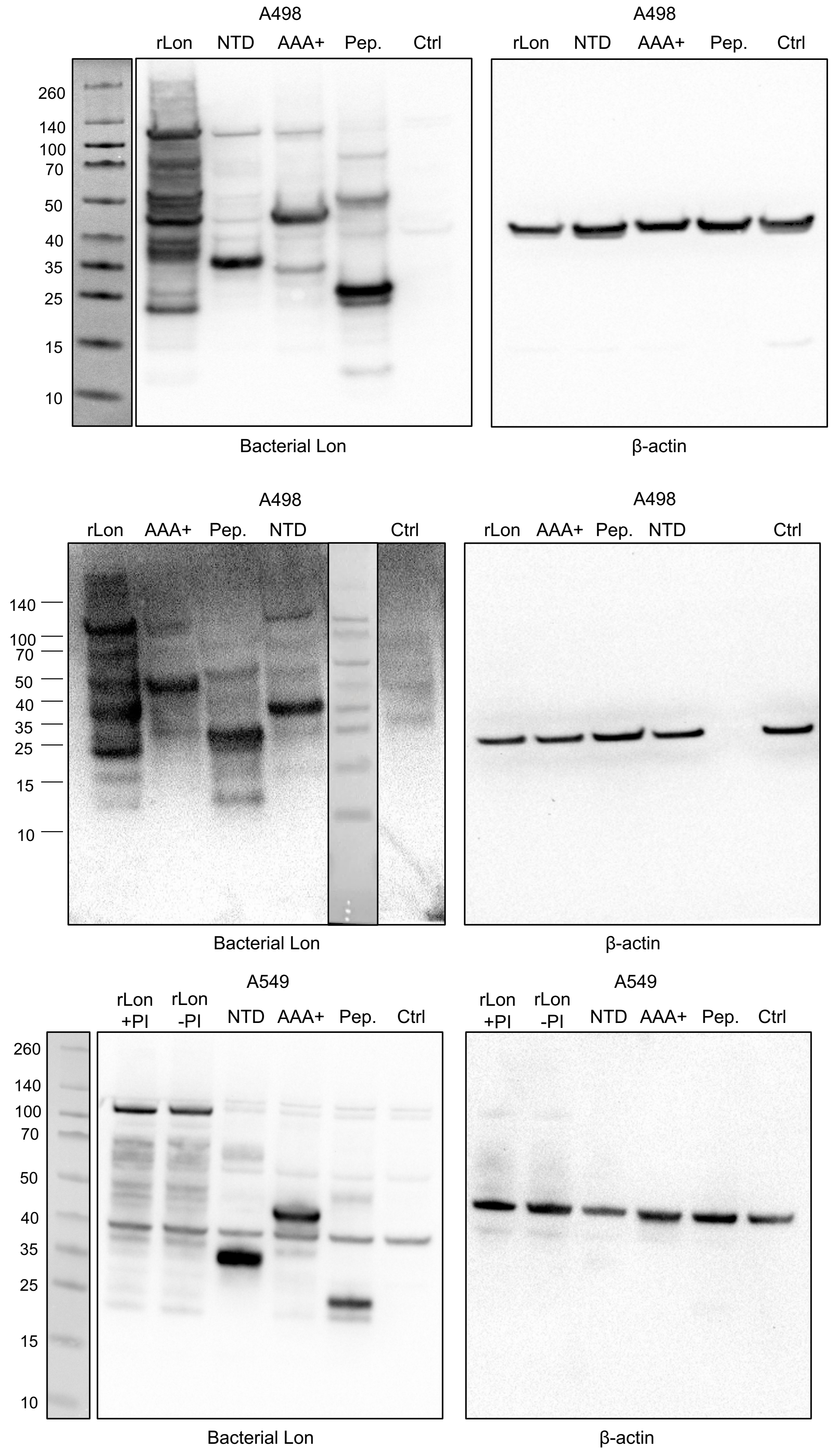

Extended Data Fig. 11. Supplementary Data for cellular uptake experiment for Main Fig. 3.
Full-length Western blot showing the repeat experiments of the cellular uptake of rLon and the NTD, peptidase and AAA+ domains in A498 cells.

Extended Data Fig. 12

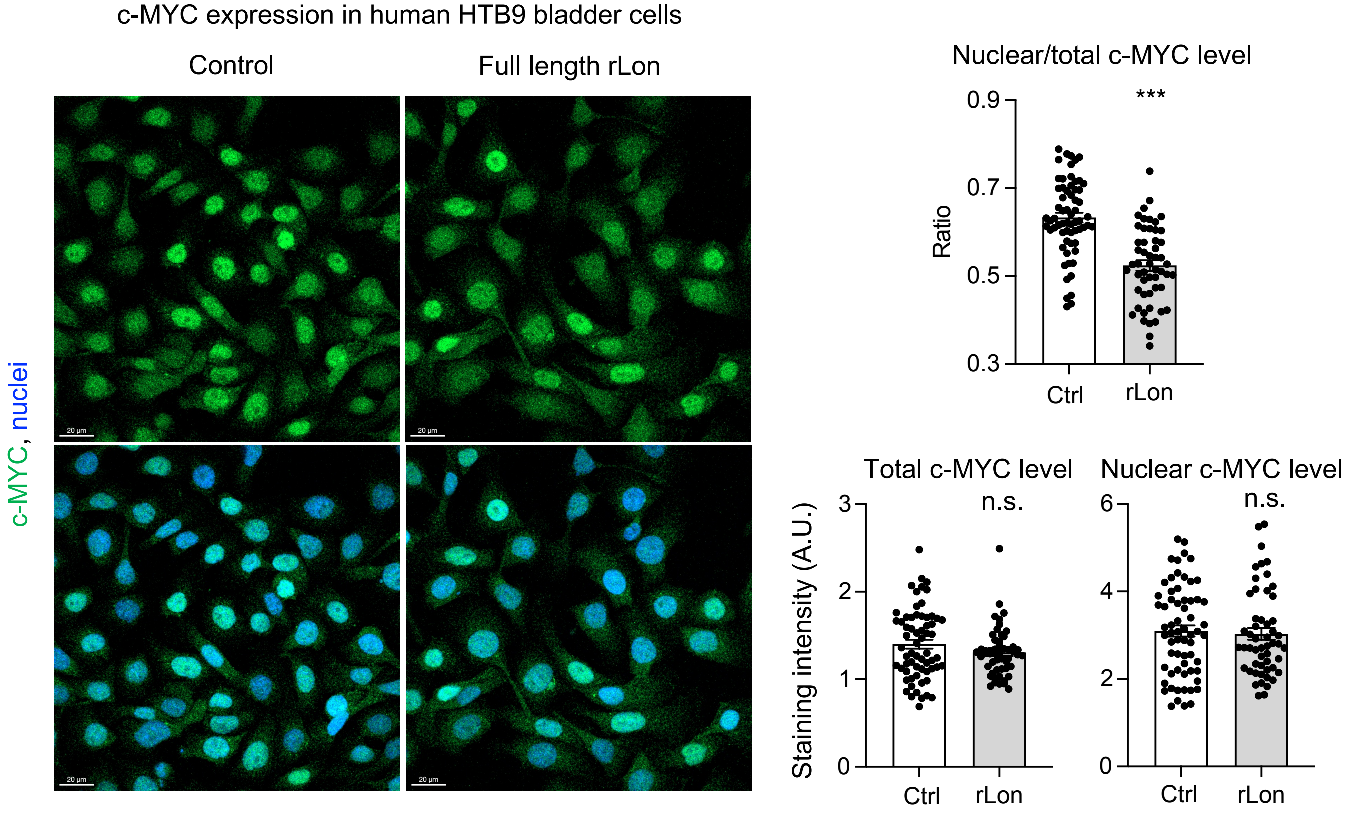

Extended Data Fig. 12. Supplementary Data for Main Fig. 3.
Confocal images showing c-MYC protein levels in rLon-treated HTB9 bladder cells compared to control (green, c-MYC; blue, nuclei; scale bars, 20 µm). Data are presented as the means ± s.e.m., total and nuclear c-MYC levels analyzed using Mann-Whitney test, ratio analyzed using unpaired t-test (n = 50 cells). ***$P < 0.001$, compared to control. n.s. = not significant.

Extended Data Fig. 13

c-MYC, N-MYC and L-MYC expression in human A498 kidney cells by confocal microscopy

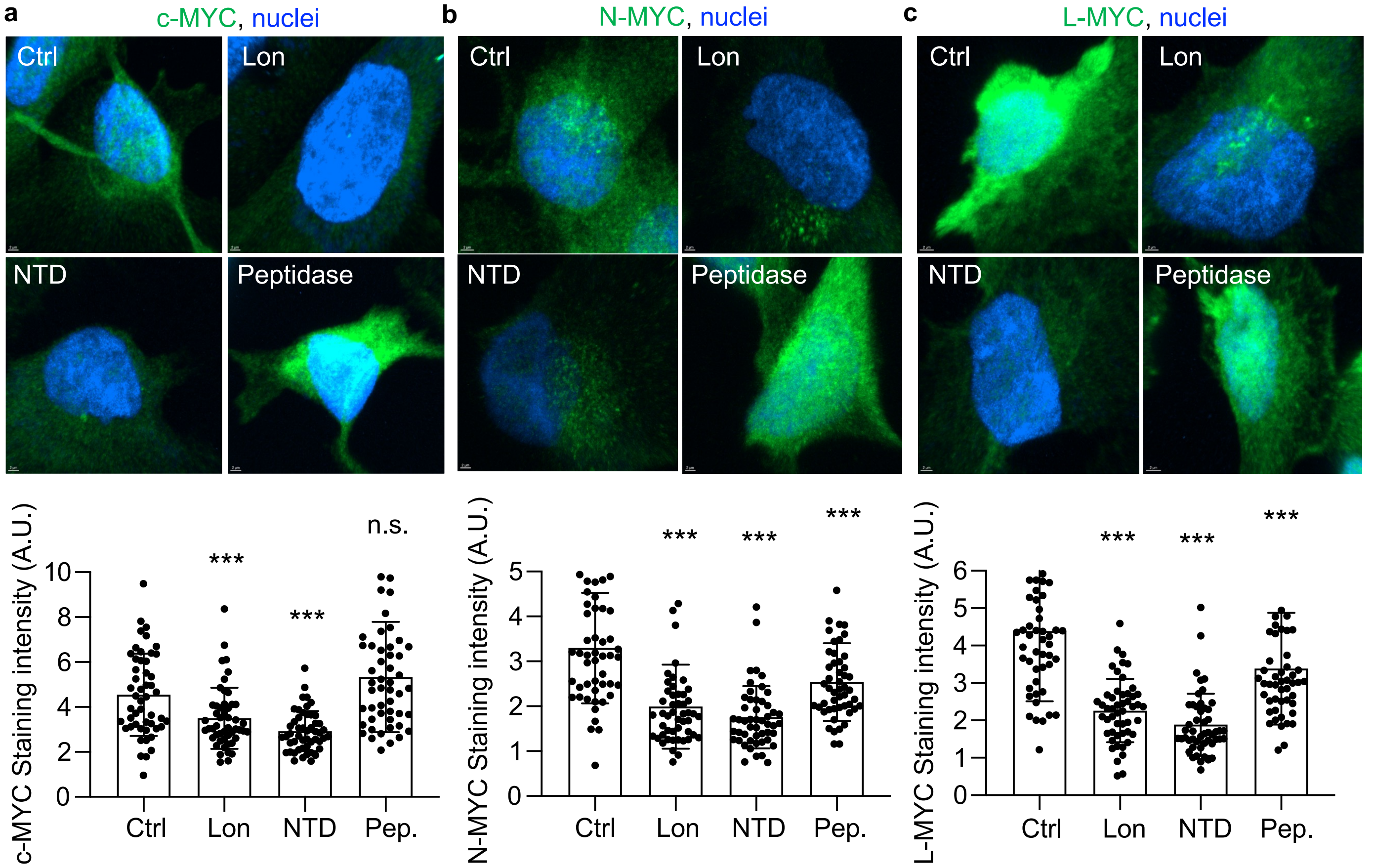

c-MYC, N-MYC and L-MYC expression in AAA+ treated human A498 kidney cells by confocal microscopy

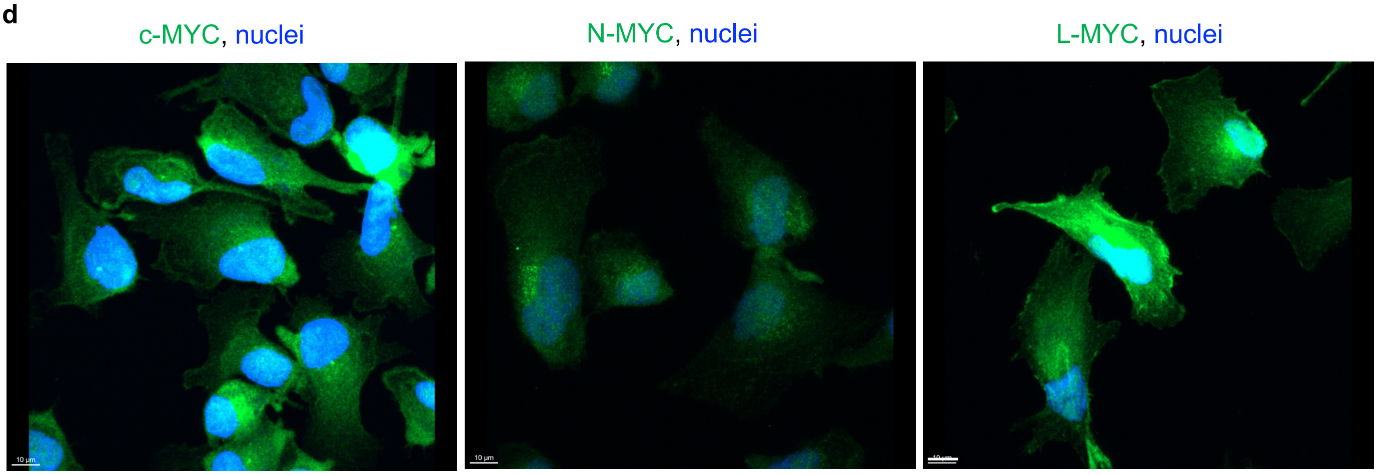

Extended Data Fig. 13. Supplementary Data for Main Fig. 3.
a-c, Confocal images showing inhibition of c-MYC (**a**), N-MYC (**b**) and L-MYC (**c**) protein levels in rLon-, NTD- or peptidase-treated cells compared to control. (green, c-MYC, N-MYC or L-MYC; blue, nuclei; scale bars, 10 μm). Lower panel: quantification of fluorescence intensity (50 cells per sample, $n = 2$ experiments). Data are presented as the mean ± s.d., analyzed by One-way ANOVA with Dunn's correction. ***$P < 0.001$, compared to control. n.s. = not significant. **d**, Confocal images showing c-MYC, N-MYC and L-MYC protein levels in AAA+ -treated cells compared to control (green, c-MYC, N-MYC or L-MYC; blue, nuclei; scale bars, 10 μm).

Extended Data Fig. 14

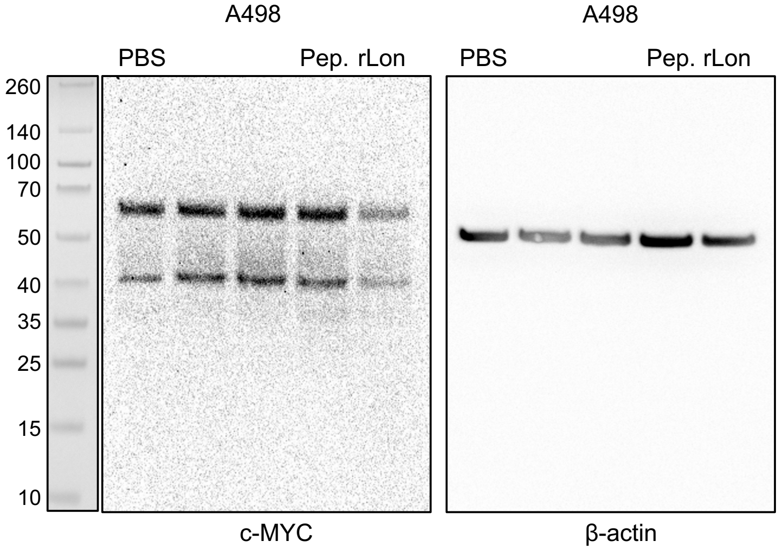

% c-MYC inhibition compared to PBS			
	**A498**	**A549**	**HTB9**
Peptidase	28	24	40

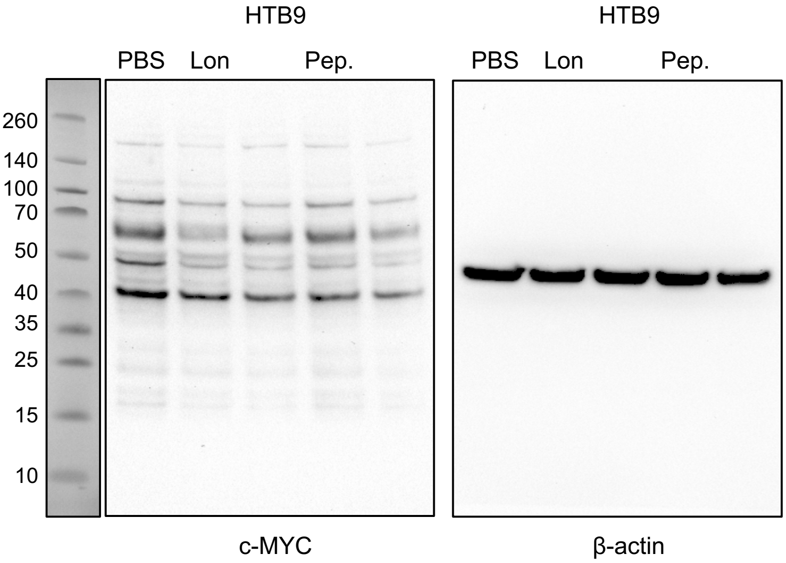

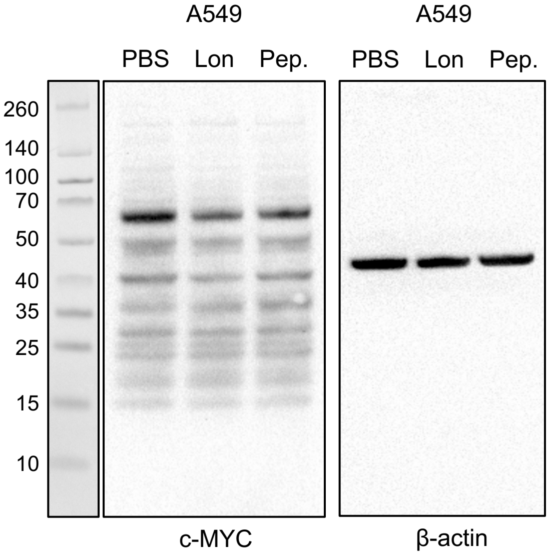

Extended Data Fig. 14. Unedited Western blot images.
Western blot analysis of c-MYC inhibition by rLon and peptidase in A498, HTB9 and A549 cells.

Extended Data Fig. 15

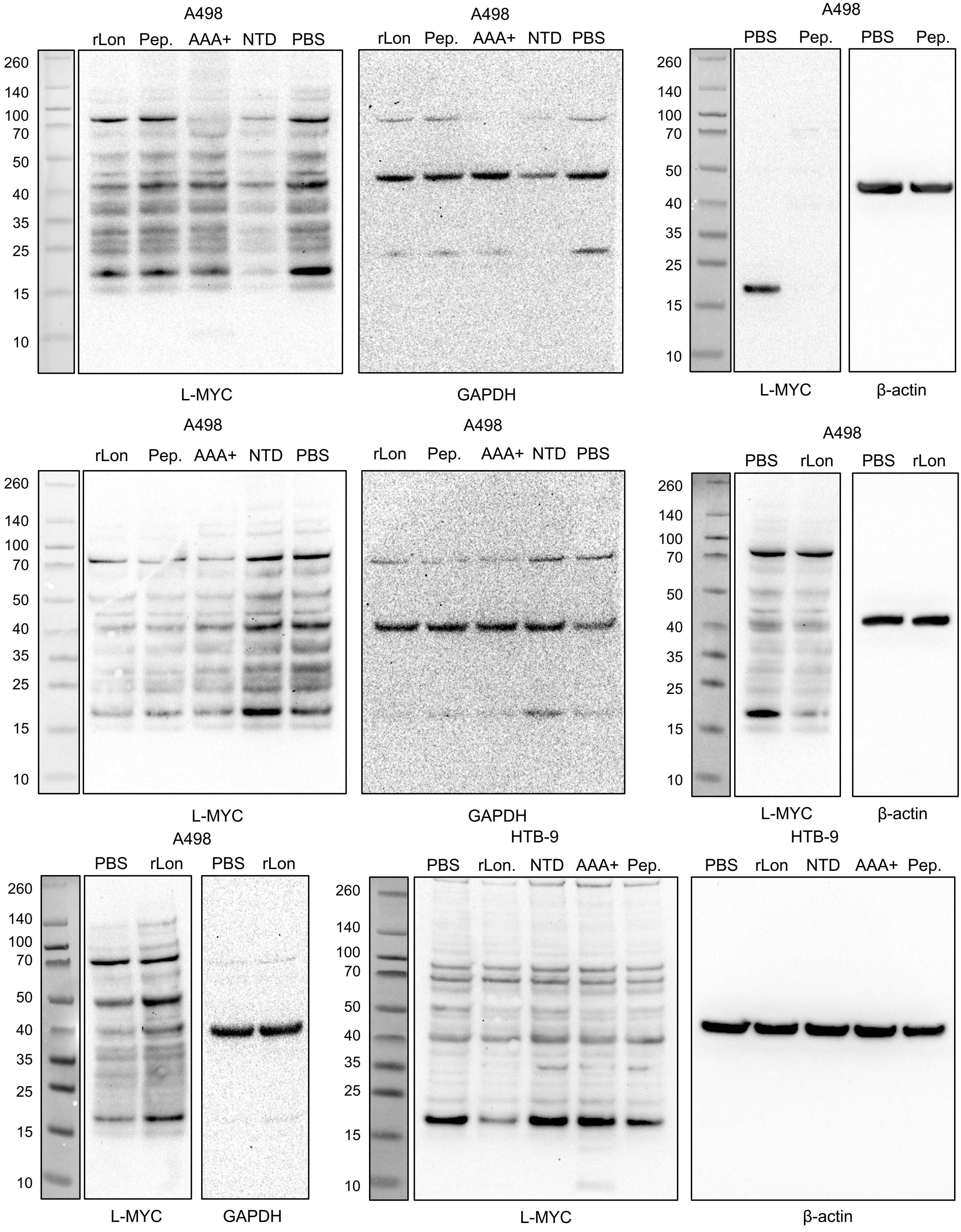

Extended Data Fig. 15. Unedited Western blot images.
Western blot analysis of L-MYC inhibition by rLon, NTD, AAA+ and peptidase in A498 and HTB9 cells.

Extended Data Fig. 16

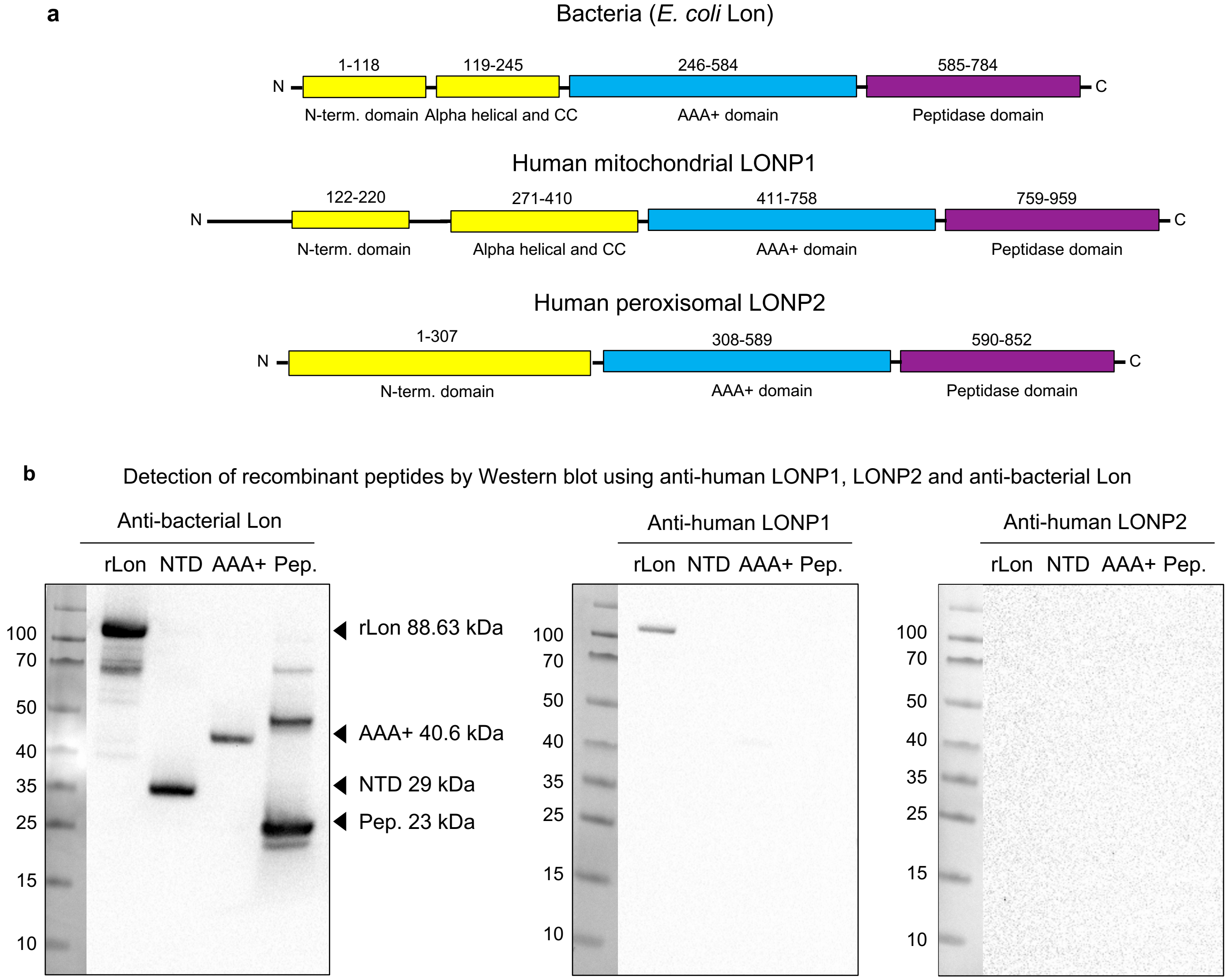

Extended Data Fig. 16. Domain organization of Lon protease across species and cross-reactivity of bacterial and human Lon antibodies
a, Domain organization of *E. coli* K12 Lon and the two human LON (mitochondrial LONP1 and peroxisomal LONP2). Domain boundaries shown are based on sequence alignment as well as experimental and *in silico* structural data. **b**, Detection of recombinant peptides by Western blot using anti-human LONP1, LONP2 and anti-bacterial Lon.

Extended Data Fig. 17

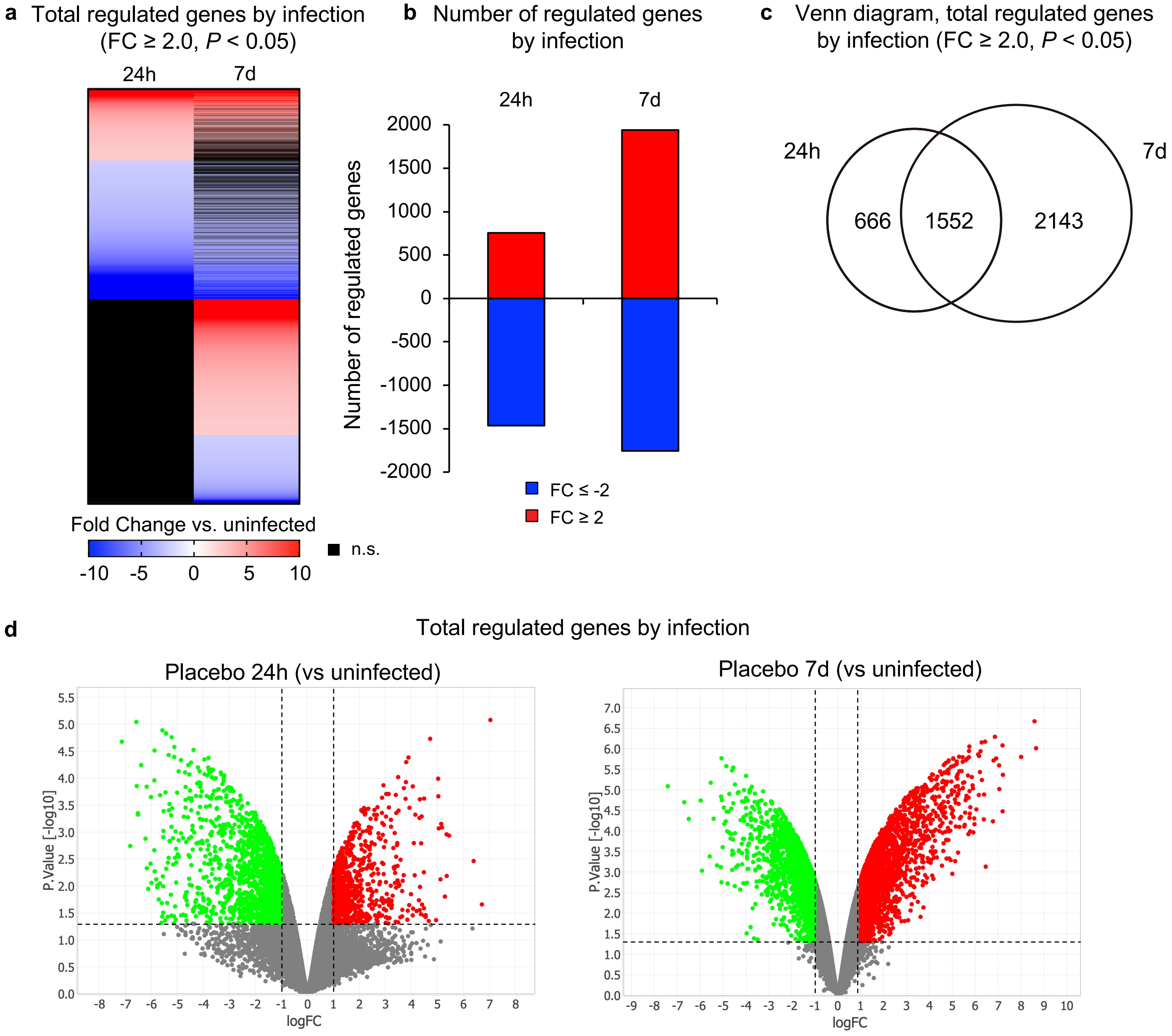

Extended Data Fig. 17. Supplementary Data for Main Fig. 4.
Irf3$^{-/-}$ mice were infected with the uropathogenic *E. coli* strain CFT073, and tissues were obtained for gene expression analysis after 24 hours and seven days (1st experiment). **a-c,** Heatmap (**a**), histogram (**b**) and Venn diagram (**c**) comparing the gene expression profiles of infected mice after 24 hours and seven days to those of uninfected mice ($n = 2$ per group; red, upregulated; blue, downregulated; cutoff FC ≥ 2, P < 0.05 compared to uninfected mice). **d,** Activated gene networks in infected *Irf3*$^{-/-}$ mice after 24 hours and a further increase in gene expression after seven days.

Extended Data Fig. 18

Immunohistochemistry of tissue sections from Placebo-treated mice 24h (c-MYC)

Immunohistochemistry of tissue sections from uninfected control mice (c-MYC)

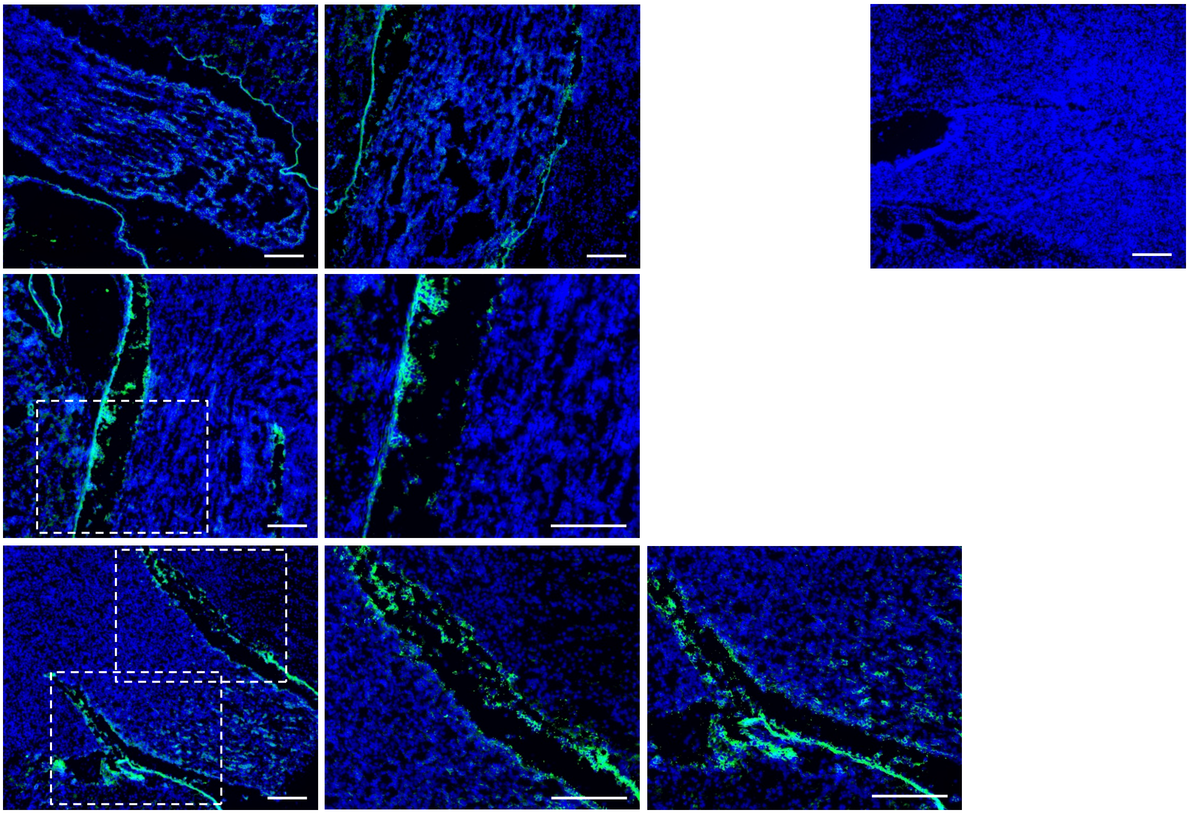

Immunohistochemistry of tissue sections from rLon-treated mice 24h (c-MYC)

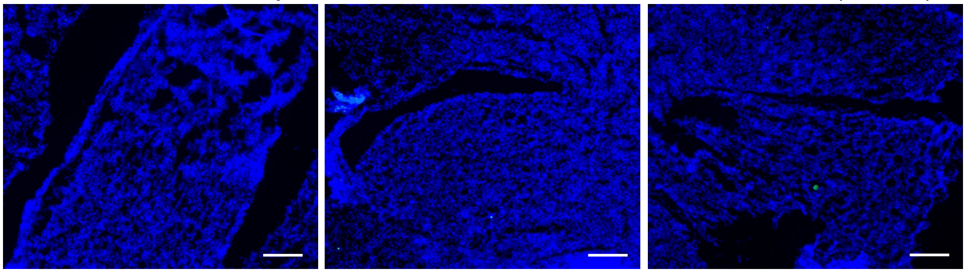

Immunohistochemistry of tissue sections from Placebo-treated mice 7d (c-MYC)

Immunohistochemistry of tissue sections from rLon-treated mice 7d (c-MYC)

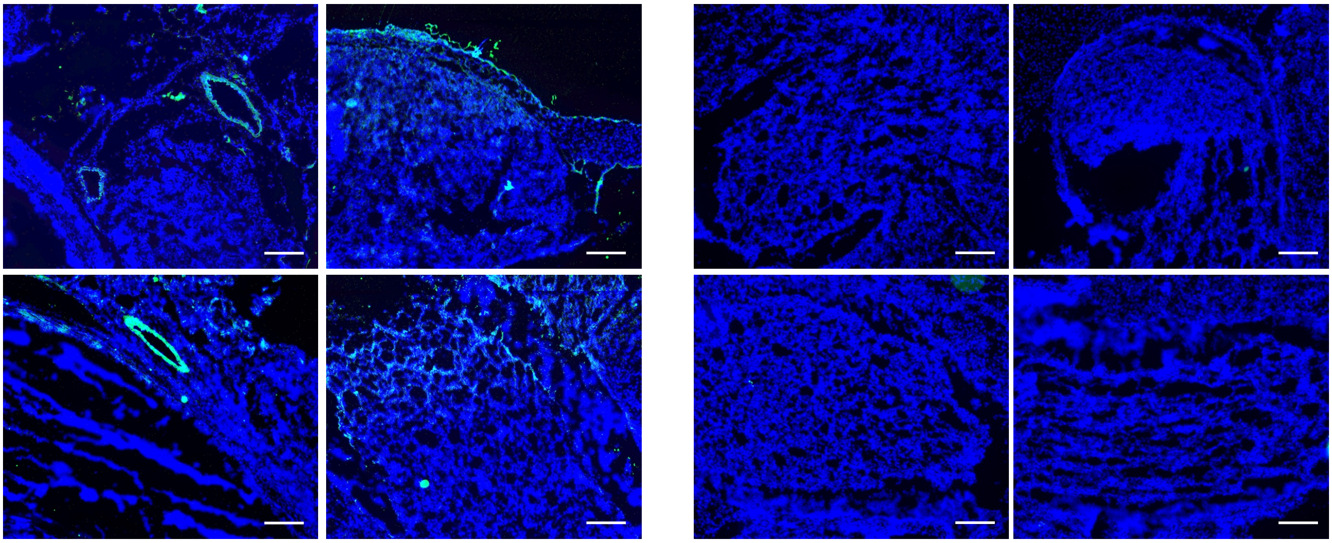

Extended Data Fig. 18. Supplementary Data for Main Fig. 4.
c-MYC protein staining in kidney tissue sections of the rLon-treated group compared to the placebo group. Confocal microscopy, representative images were shown in Main Fig. 4; *n* = 3-7 mice per group; green, c-MYC; blue, nuclei; scale bar, 20 μm.

Extended Data Fig. 19

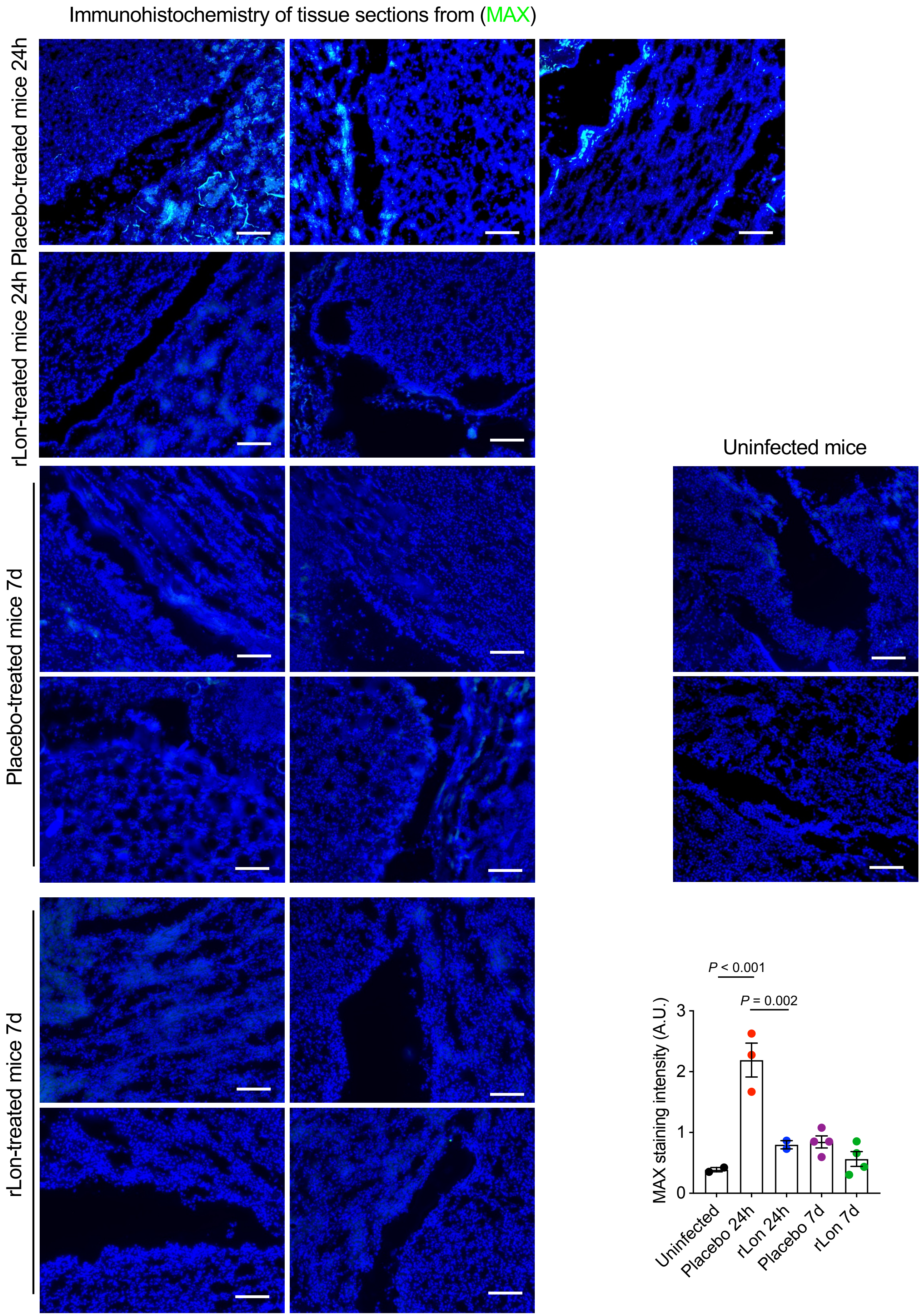

Extended Data Fig. 19. Supplementary Data for Main Fig. 4.
MAX protein staining in kidney tissue sections of the rLon-treated group compared to the placebo group. Confocal microscopy, n = 4-5 mice per group; green, MAX; blue, nuclei; scale bar, 20 µm. Data are presented as the mean ± s.e.m., one-way ANOVA followed by Sidak's multiple comparisons test.

Extended Data Fig. 20

a Total regulated genes (FC ≥ 2.0, *P* < 0.05)

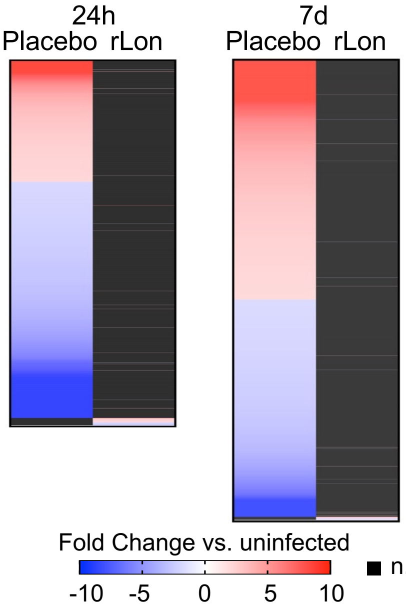

1st experiment

b Number of regulated genes

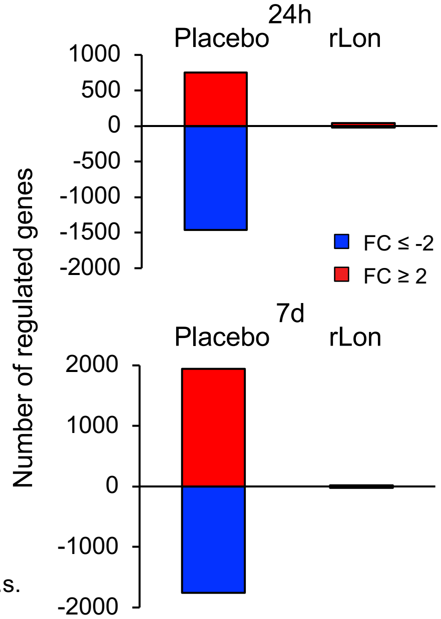

c Venn diagram, total regulated genes, (FC ≥ 2.0, *P* < 0.05)

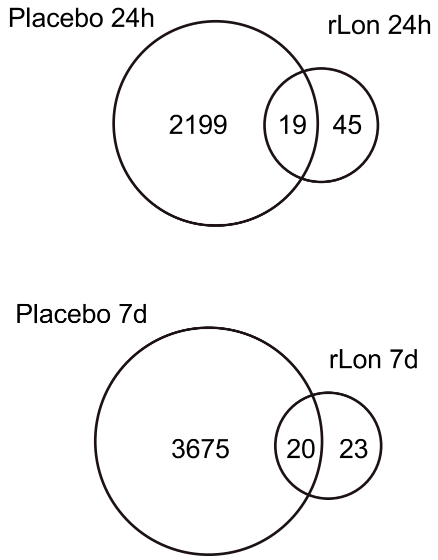

d

Canonical Pathways	Placebo 24h	rLon 24h	Placebo 7d	rLon 7d
Immunogenic Cell Death	4.7	<0.1	5.0	<0.1
Macrophage Activation	4.6	<0.1	7.5	<0.1
Granzyme A	4.5	<0.1	4.7	<0.1
Rheumatoid Arthritis	4.4	<0.1	7.6	<0.1
Ceramide	4.1	<0.1	1.7	<0.1
Neuroinflammation	3.9	<0.1	6.7	<0.1
PI3K/AKT	3.8	<0.1	3.7	<0.1
Dendritic Cell Maturation	3.8	<0.1	7.2	<0.1
Xenobiotic Metabolism	-3.0	<0.1	-3.1	<0.1
TCA Cycle	-3.3	<0.1	-4.1	<0.1
Methionine Degradation	-3.3	<0.1	-3.6	<0.1
Valine Degradation I	-3.3	<0.1	-3.5	<0.1
NAD	-3.6	<0.1	0.1	<0.1
PPAR	-3.8	<0.1	-6.2	<0.1
LXR/RXR Activation	-4.4	<0.1	-4.7	<0.1
Oxidative Phosphorylation	-6.6	<0.1	-8.1	<0.1

e

Biofunctions	Placebo 24h	rLon 24h	Placebo 7d	rLon 7d
Adhesion of blood cells	5.2	<0.1	9.1	<0.1
Immune response	4.6	<0.1	7.6	<0.1
Phagocytosis	4.4	<0.1	7.3	<0.1
Cell movement	4.1	<0.1	10.3	<0.1
Urination disorder	4.0	<0.1	2.8	<0.1
Chemotaxis	3.8	<0.1	8.2	<0.1
Aciduria	3.7	<0.1	N/A	<0.1
Synthesis of protein	3.7	<0.1	N/A	<0.1
Structural renal abnormality	3.6	<0.1	N/A	<0.1
Inflammatory response	3.6	<0.1	7.9	<0.1
Amyloidosis	-2.1	<0.1	-1.6	<0.1
Gluconeogenesis	-2.1	<0.1	N/A	<0.1
Transport of lipid	-2.5	<0.1	N/A	<0.1
Infection	-3.5	<0.1	-8.4	<0.1
Nucleic acid metabolism	-3.6	<0.1	-0.5	<0.1

f MYC-dependent innate immune response to infection, 24h Placebo vs uninfected

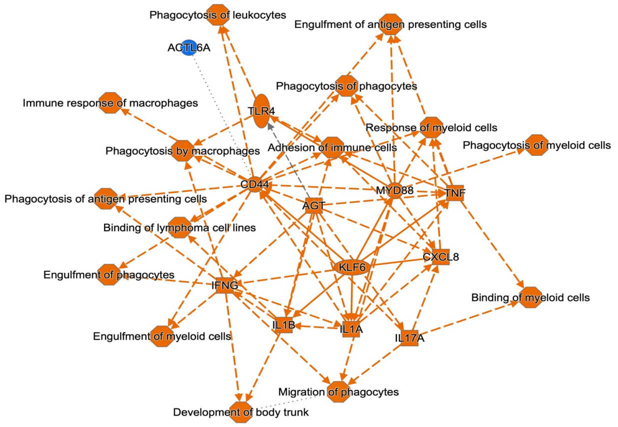

g Inhibition of the MYC-dependent response by rLon, 24h rLon vs Placebo

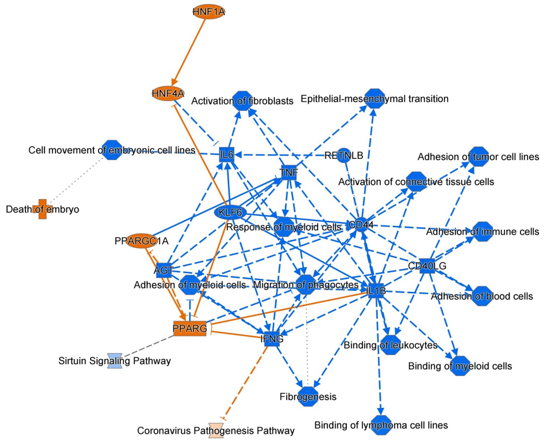

Extended Data Fig. 20. Supplementary Data for Main Fig. 4.

a-c, Heatmap (**a**), histogram (**b**) and Venn diagram (**c**) comparing the gene expression profiles of rLon-treated mice after 24 hours and seven days to those of uninfected mice ($n = 2$ per group; red, upregulated; blue, downregulated; cutoff FC ≥ 2, $P < 0.05$ compared to uninfected mice). **d, e,** Canonical pathways (**d**) and biofunction (**e**) by placebo and rLon-treated groups compared to those of uninfected mice (red, upregulated; blue, downregulated). The activation Z scores are shown. Data normalized against healthy, uninfected *Irf3*$^{-/-}$ mice. **f,** Activated MYC-dependent gene networks in infected *Irf3*$^{-/-}$ mice after 24 hours, including an innate immune response network. **g**, Inhibition of the innate immune response to infection by rLon treatment (orange, activation; blue, inhibition).

Extended Data Fig. 21

2nd experiment

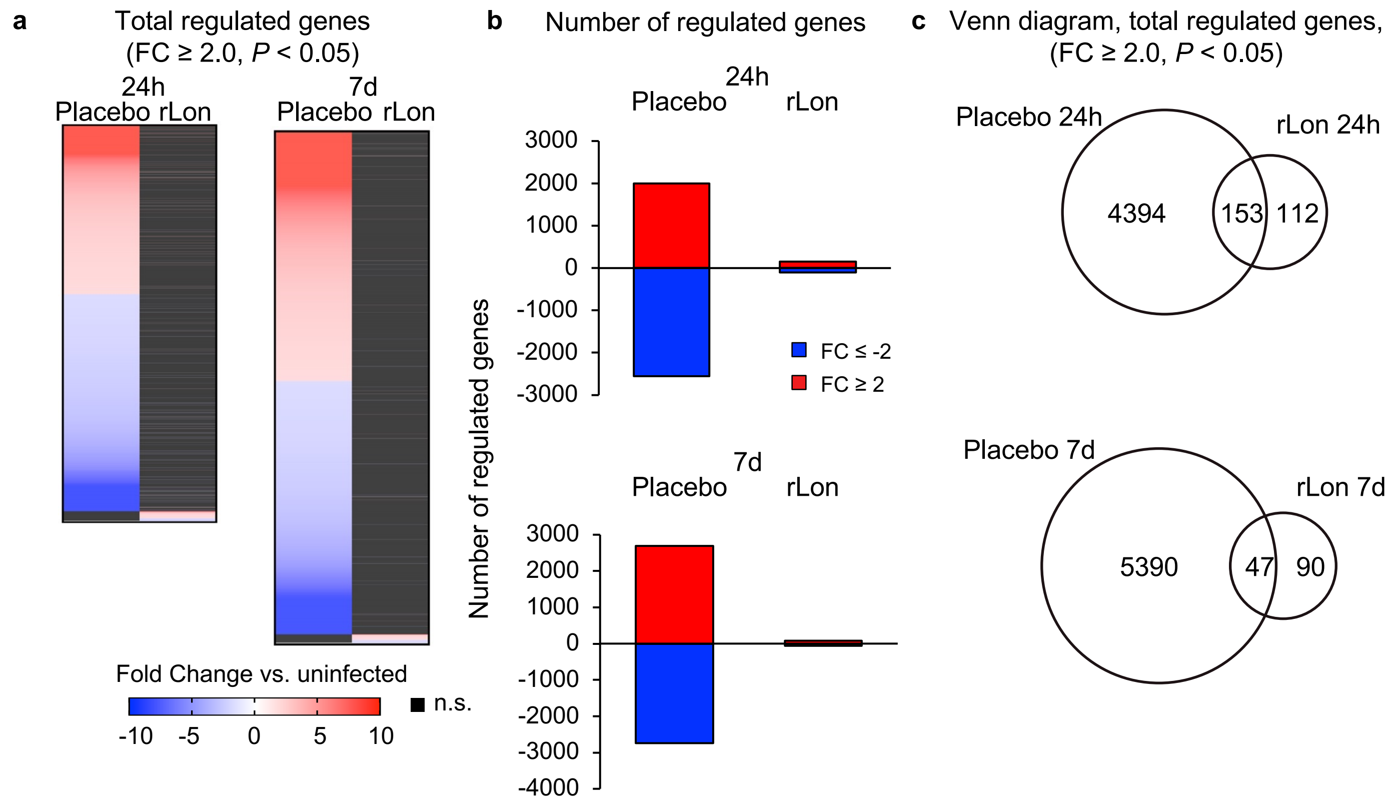

d

Canonical Pathways	Placebo 24h	rLon 24h	Placebo 7d	rLon 7d
Cytokine Storm	5.9	1.9	9.3	<0.1
Acute Phase Response	5.3	1.0	6.4	<0.1
IL-6	5.3	<0.1	6.3	<0.1
Rheumatoid Arthritis	5.2	<0.1	7.8	<0.1
HMGB1	4.6	<0.1	5.4	<0.1
Mitochondrial Dysfunction	4.6	-0.4	7.0	<0.1
Natural Killer Cell	4.6	<0.1	4.9	<0.1
Tumor Microenvironment Pathway	4.5	<0.1	4.9	<0.1
Methionine Degradation	-2.8	<0.1	-2.2	<0.1
Estrogen Biosynthesis	-2.9	<0.1	-3.2	<0.1
TCA Cycle II	-3.3	<0.1	-4.0	<0.1
LXR/RXR	-3.5	1.0	-3.7	<0.1
PPARα/RXRα	-3.5	0.0	-4.4	<0.1
Valine Degradation	-3.6	<0.1	-3.9	<0.1
PPAR	-5.3	<0.1	-5.7	<0.1
Oxidative Phosphorylation	-6.1	<0.1	-8.7	<0.1

e

Biofunctions	Placebo 24h	rLon 24h	Placebo 7d	rLon 7d
Cell movement	8.8	2.3	11.4	0.8
Chemotaxis	6.7	<0.1	9.5	0.8
Adhesion of blood cells	6.2	<0.1	9.5	0.8
Inflammatory response	5.8	1.3	6.6	<0.1
Metastasis	5.5	<0.1	7.4	<0.1
Advanced tumor	5.5	<0.1	7.3	<0.1
Phagocytosis	5.3	<0.1	7.2	<0.1
Reactive oxygen species	5.1	<0.1	6.6	<0.1
Endocytosis	5.0	<0.1	6.6	<0.1
Atherosclerosis	4.8	1.2	4.7	<0.1
Urination disorder	3.6	-1.2	3.6	<0.1
Necrosis	-1.2	-1.6	-0.2	<0.1
Apoptosis	-1.7	-1.0	-1.5	<0.1
Enteritis	-1.7	0.2	-1.5	<0.1
Bleeding	-2.5	<0.1	<0.1	<0.1
Infection	-4.6	-2.0	-7.4	0.7

Extended Data Fig. 21. Supplementary Data for Main Fig. 4.
a-c, Heatmap (**a**), histogram (**b**) and Venn diagram (**c**) comparing the gene expression profiles of rLon-treated mice after 24 hours and seven days to those of uninfected mice ($n = 2$ per group; red, upregulated; blue, downregulated; cutoff FC ≥ 2, $P < 0.05$ compared to uninfected mice). **d, e,** Canonical pathways (**d**) and biofunction (**e**) by placebo and rLon-treated groups compared to those of uninfected mice (red, upregulated; blue, downregulated). The activation Z scores are shown. Data normalized against healthy, uninfected *Irf3*$^{-/-}$ mice.

Extended Data Fig. 22

2nd experiment

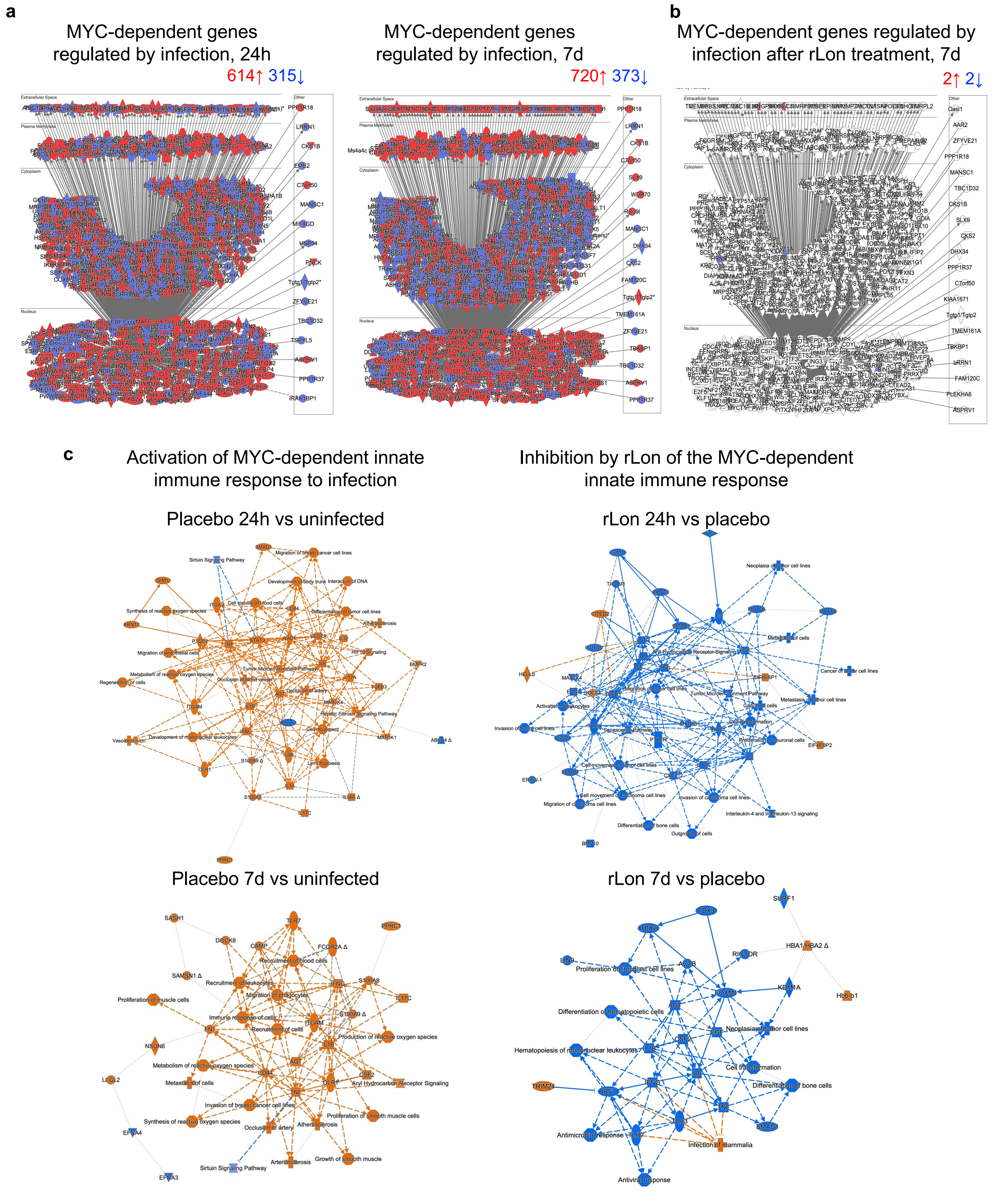

Extended Data Fig. 22. Supplementary Data for Main Fig. 4.
a, Response to kidney infection in the placebo group, showing activation of MYC-dependent genes after 24 hours and seven days (n = 2 per group; red, upregulated; blue, downregulated; cutoff FC ≥ 2, $P < 0.05$ compared to uninfected mice). **b**, Inhibition by rLon treatment of the MYC-response to infection, shown as a marked reduction in the number of MYC-dependent genes in rLon-treated mice compared to the placebo group**. c,** Activated MYC-dependent gene networks in infected *Irf3*$^{-/-}$ mice after 24 hours and 7 days, including an innate immune response network. Inhibition of the innate immune response to infection by rLon treatment (orange, activation; blue, inhibition).

Extended Data Fig. 23

a Interferon signaling pathway (vs. uninfected, FC ≥ 2.0, *P* < 0.05)

Irf3$^{-/-}$ Placebo 7d

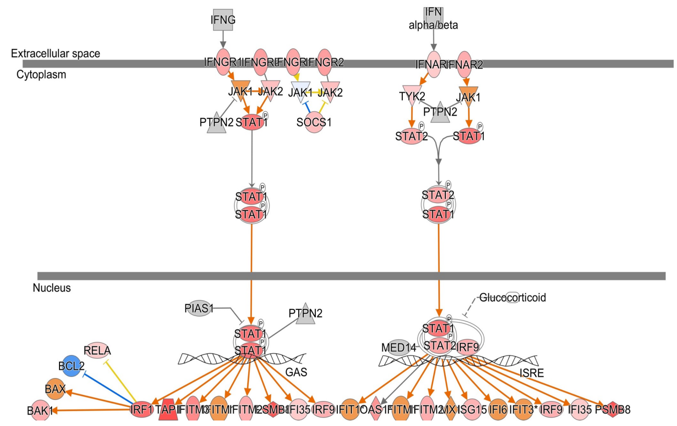

Irf3$^{-/-}$ rLon 7d

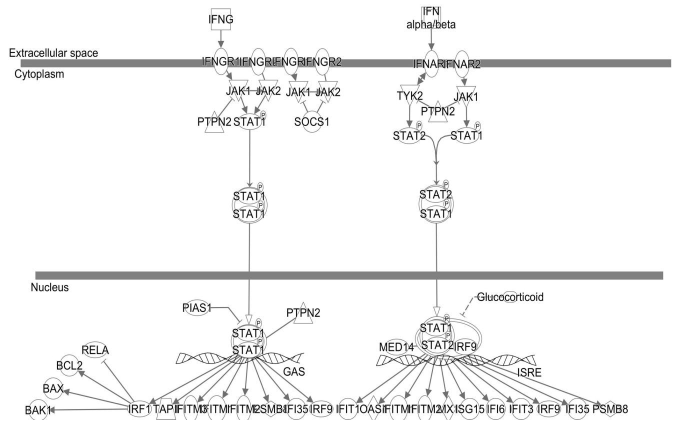

b Toll-like Receptor signaling pathway (vs. uninfected, FC ≥ 2.0, *P* < 0.05)

Irf3$^{-/-}$ Placebo 7d

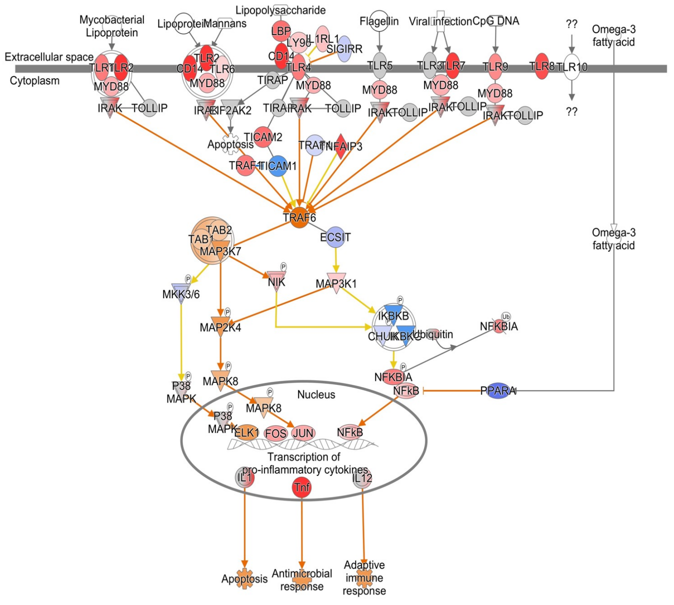

Irf3$^{-/-}$ rLon 7d

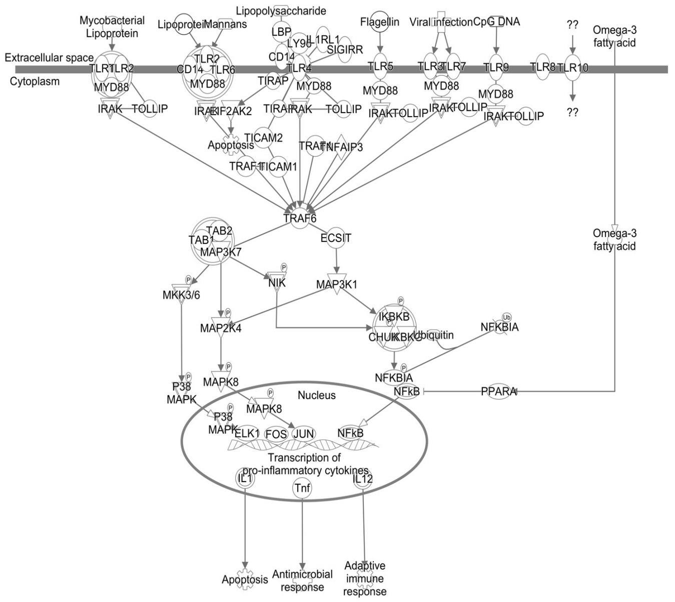

Extended Data Fig. 23

c

NF-κB signaling pathway (vs. uninfected, FC ≥ 2.0, *P* < 0.05)

Irf3$^{-/-}$ Placebo 7d

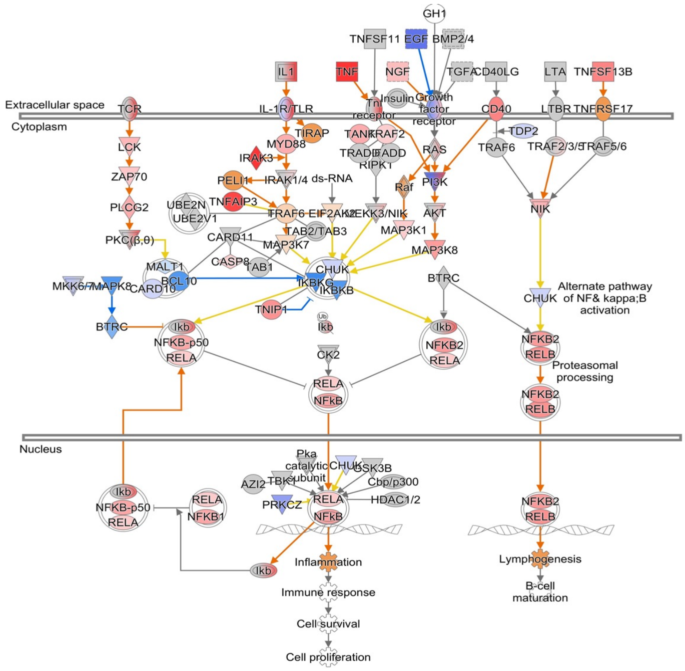

Irf3$^{-/-}$ rLon 7d

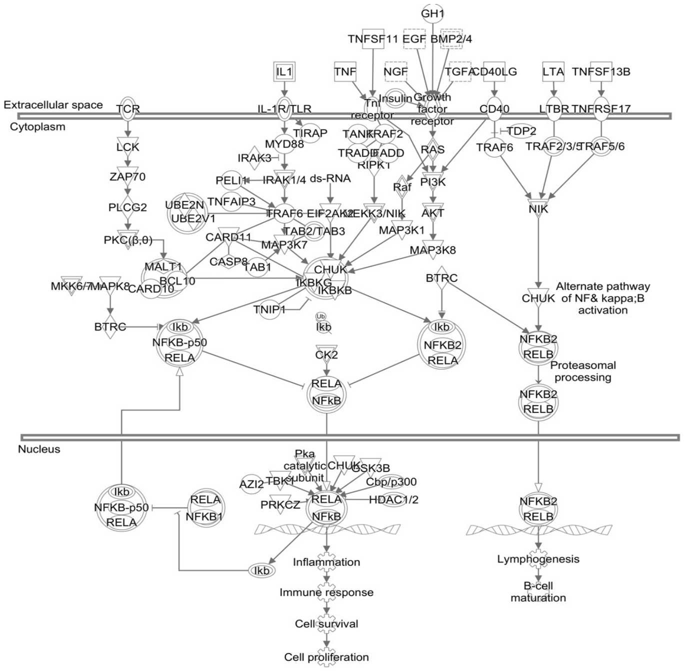

d

Inflammasome pathway (vs. uninfected, FC ≥ 2.0, *P* < 0.05)

Irf3$^{-/-}$ Placebo 7d

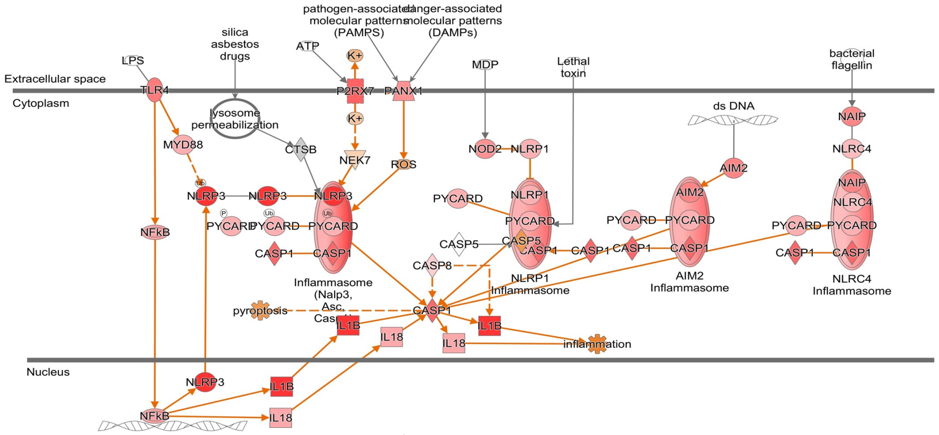

Irf3$^{-/-}$ rLon 7d

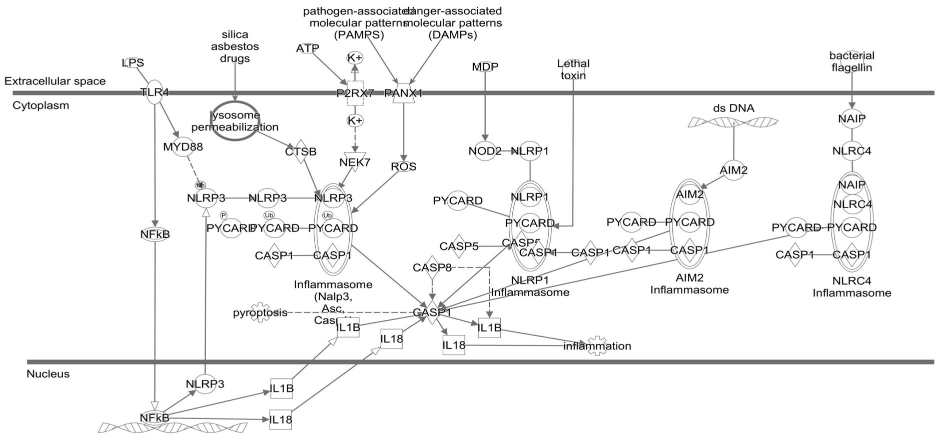

Extended Data Fig. 23. Supplementary Data for Main Fig. 4.

a-d, Pathway analysis showed that interferon signaling, toll-like receptor signaling, NF-κB signaling and inflammasome pathways were inhibited by rLon treatment in *Irf3*$^{-/-}$ mice (*n* = 2 per group; red, upregulated; blue, downregulated; cutoff FC ≥ 2, *P* < 0.05 compared to uninfected mice).

Extended Data Fig. 24

Irf3$^{-/-}$ rLon vs placebo 24h GSEA

1st experiment

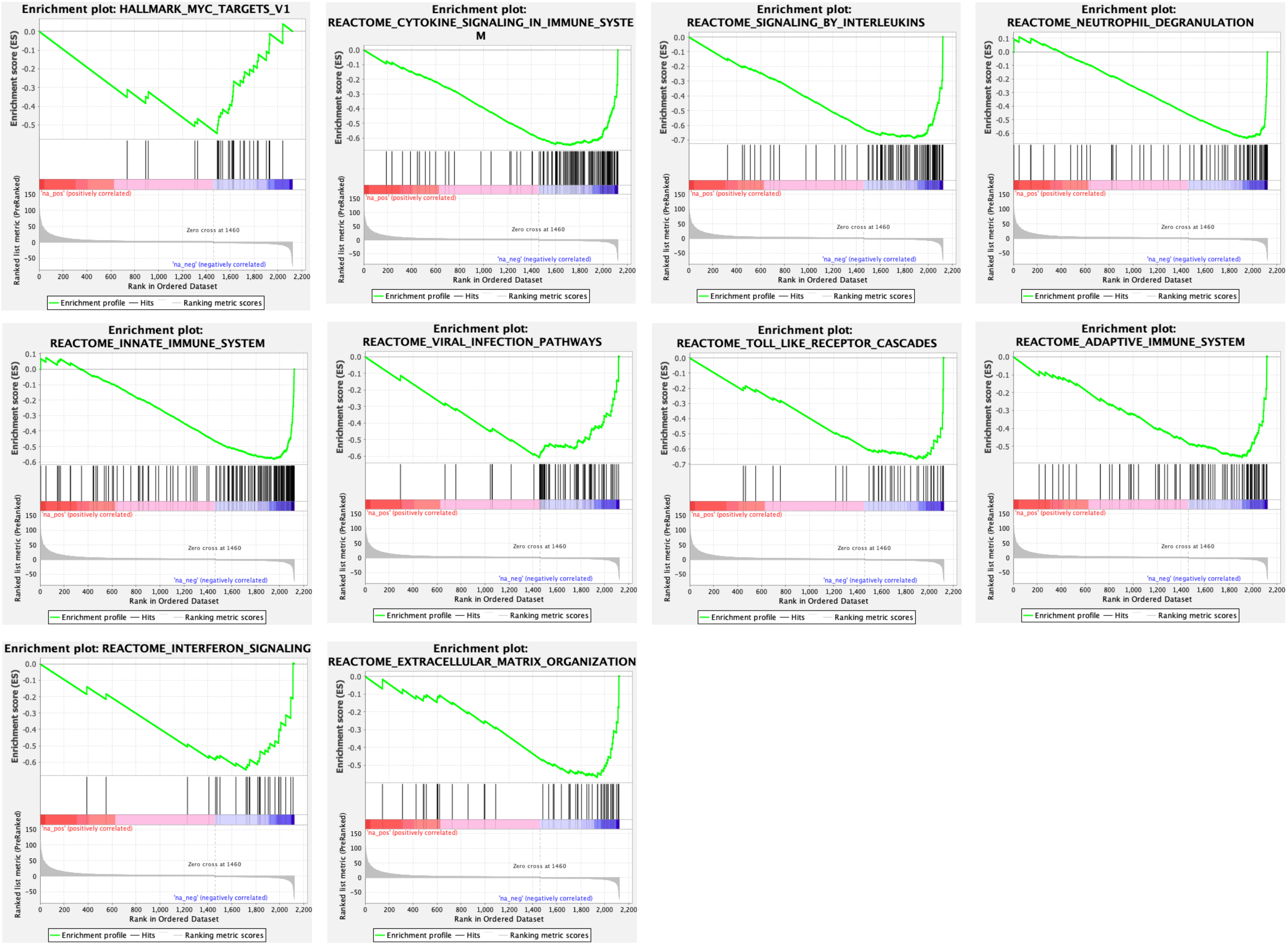

2nd experiment

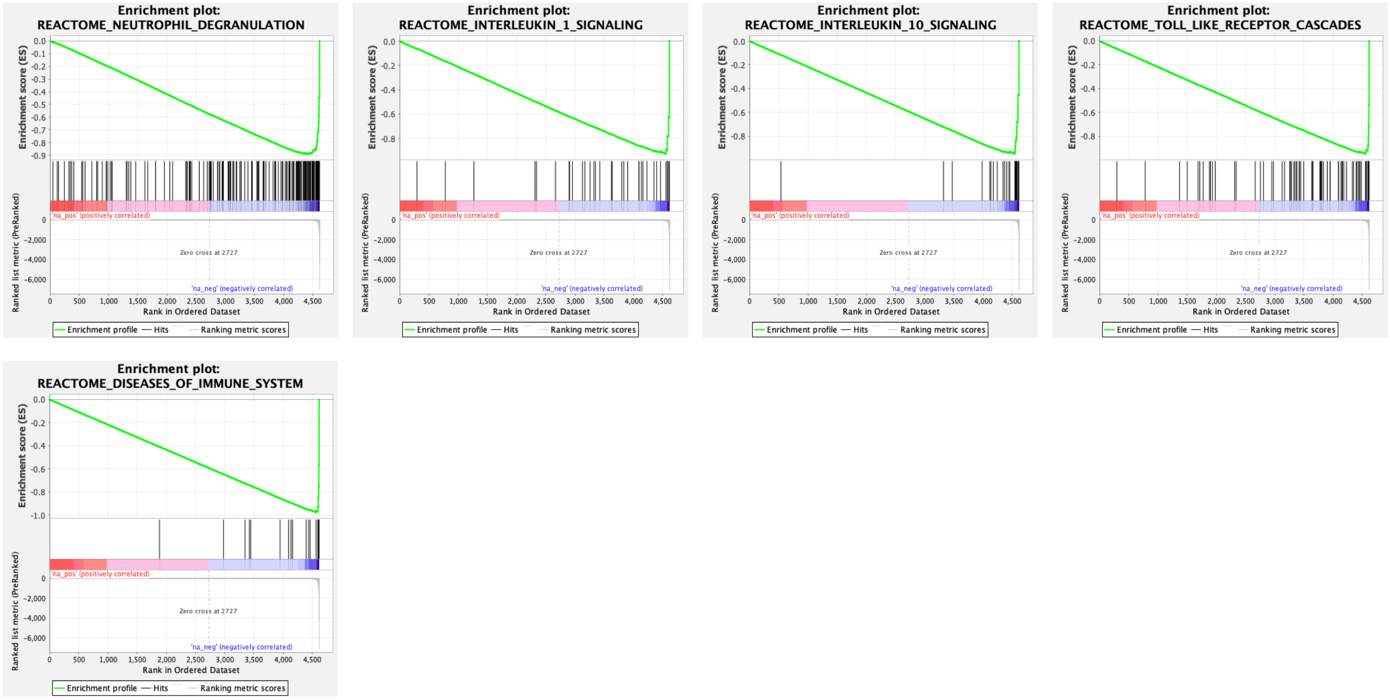

Extended Data Fig. 24

Irf3$^{-/-}$ rLon vs placebo 7d GSEA

1st experiment

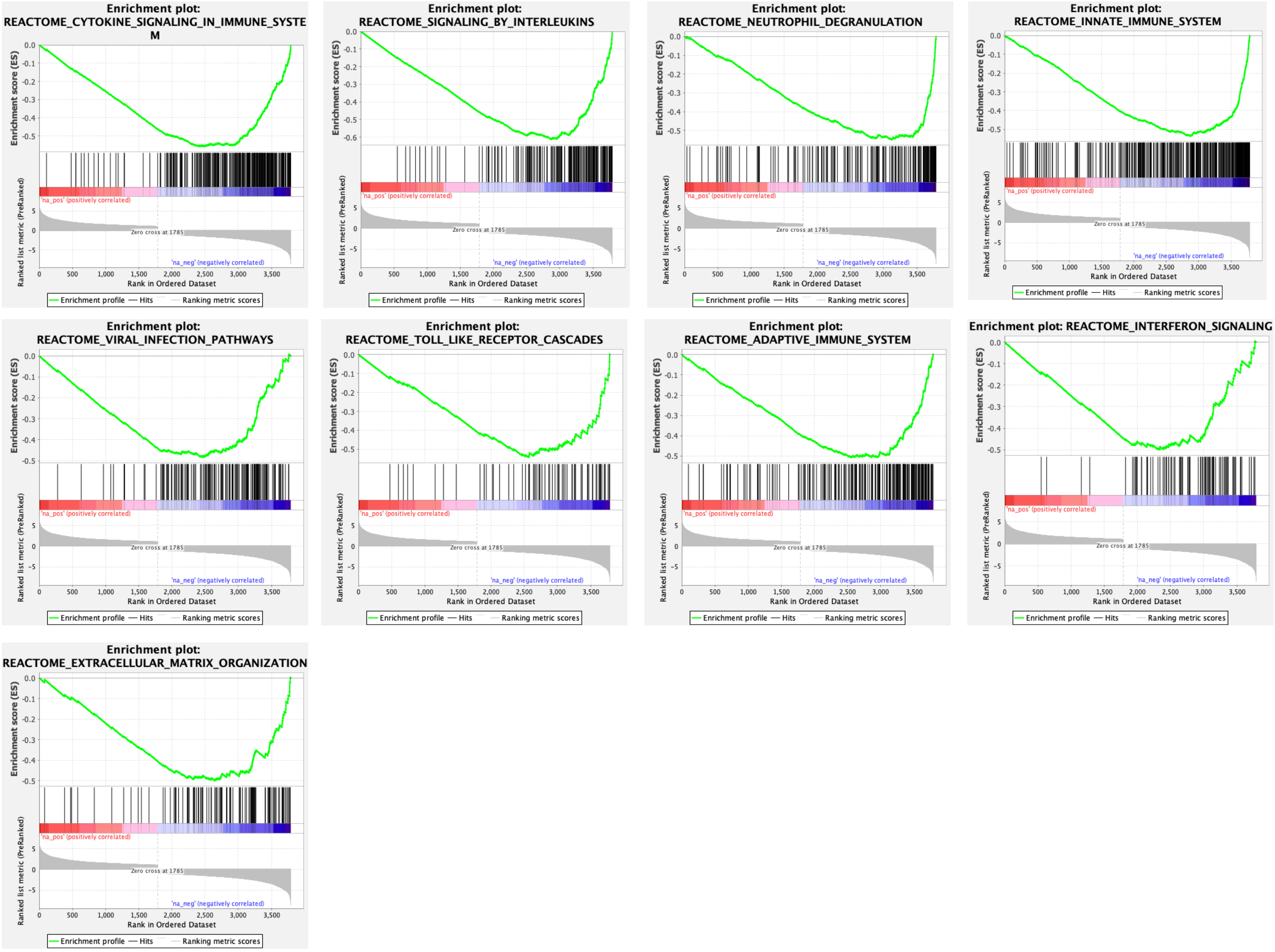

2nd experiment

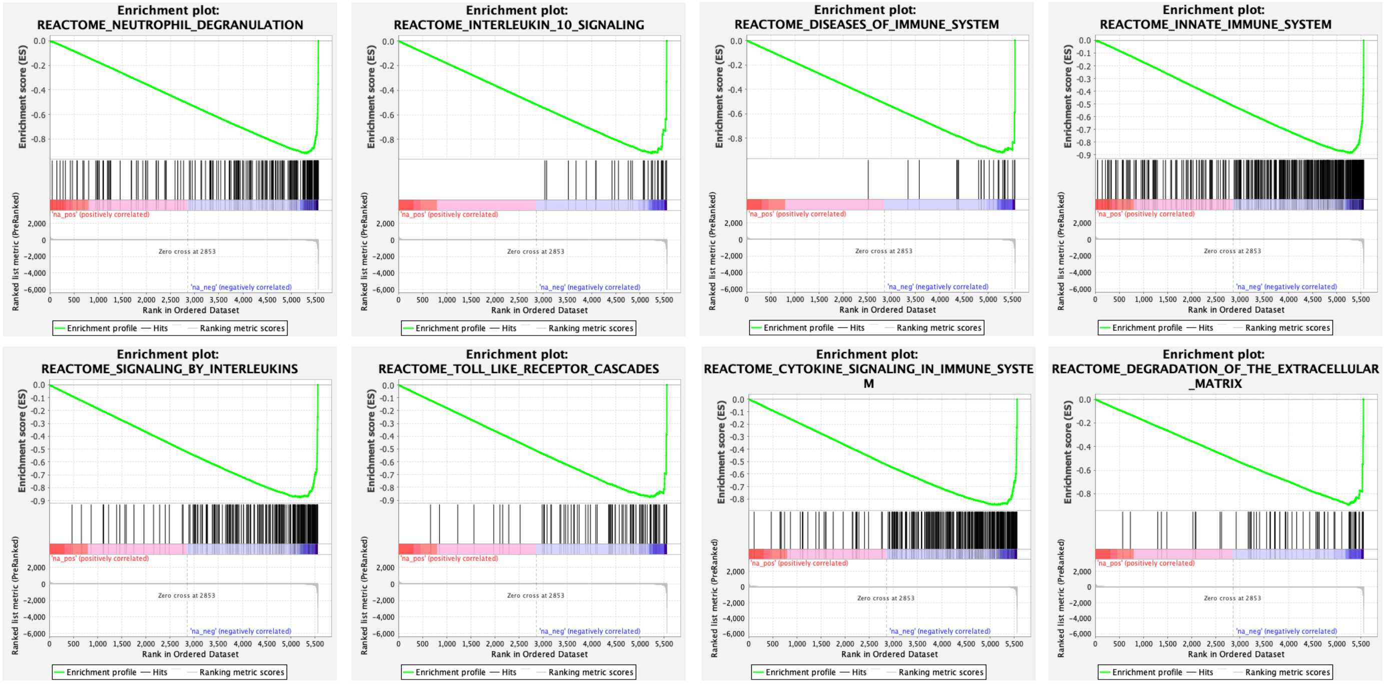

Extended Data Fig. 24. Supplementary Data for Main Fig. 4.
Suppression of the innate immune responses in the rLon-treated mice after 24 hours and seven days was further analyzed by Gene Set Enrichment Analysis (GSEA), which confirmed the downregulation of critical immune pathways and MYC target genes. Two independent experiments are shown.

Extended Data Fig. 25

2nd experiment

N-MYC-dependent genes regulated by infection, 24h

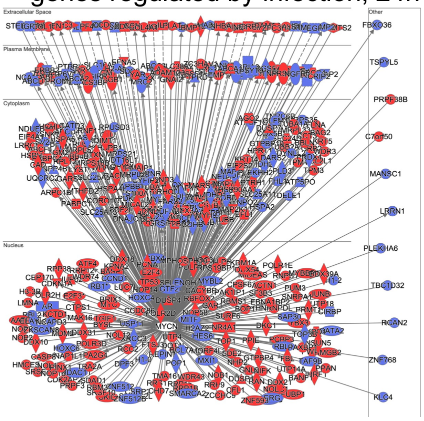

N-MYC-dependent genes regulated by rLon, 24h

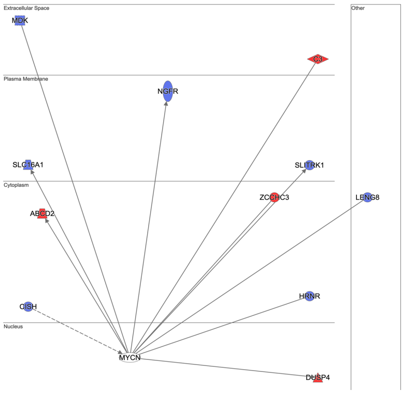

Number of regulated N-MYC dependent genes

	Placebo	
	**24h**	**7d**
Up	253	264
Down	139	182
Total	392	446

	rLon	
	**24h**	**7d**
Up	4	1
Down	7	2
Total	11	3

N-MYC-dependent genes regulated by infection, 7d

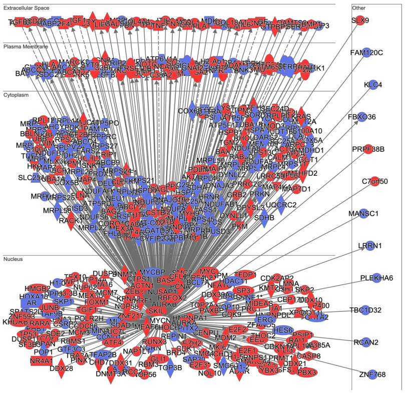

N-MYC-dependent genes regulated by rLon, 7d

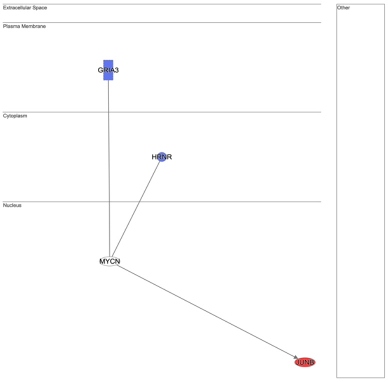

L-MYC-dependent genes regulated by infection, 24h

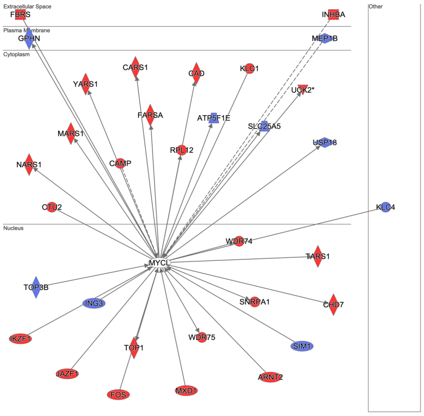

L-MYC-dependent genes regulated by rLon, 24h

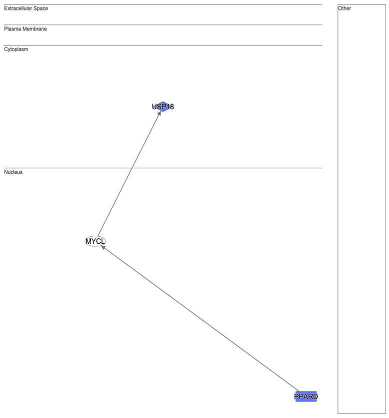

Number of regulated L-MYC dependent genes

	Placebo	
	**24h**	**7d**
Up	24	26
Down	9	18
Total	33	44

	rLon	
	**24h**	**7d**
Up	0	0
Down	2	0
Total	2	0

L-MYC-dependent genes regulated by infection, 7d

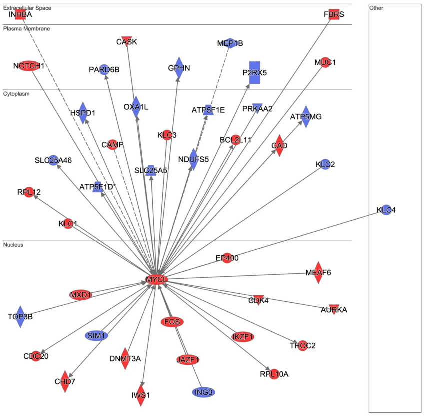

Extended Data Fig. 25. Supplementary Data for Main Fig. 5. Network analysis of N-MYC- and L-MYC-dependent genes regulated by *E. coli* CFT073 infection in *Irf3*$^{-/-}$ mice. The N-MYC and L-MYC response to kidney infection was inhibited by rLon treatment (n = 2 mice per group from 2nd experiment; cutoff FC ≥ 2; $P < 0.05$ compared to uninfected mice).

Extended Data Fig. 26

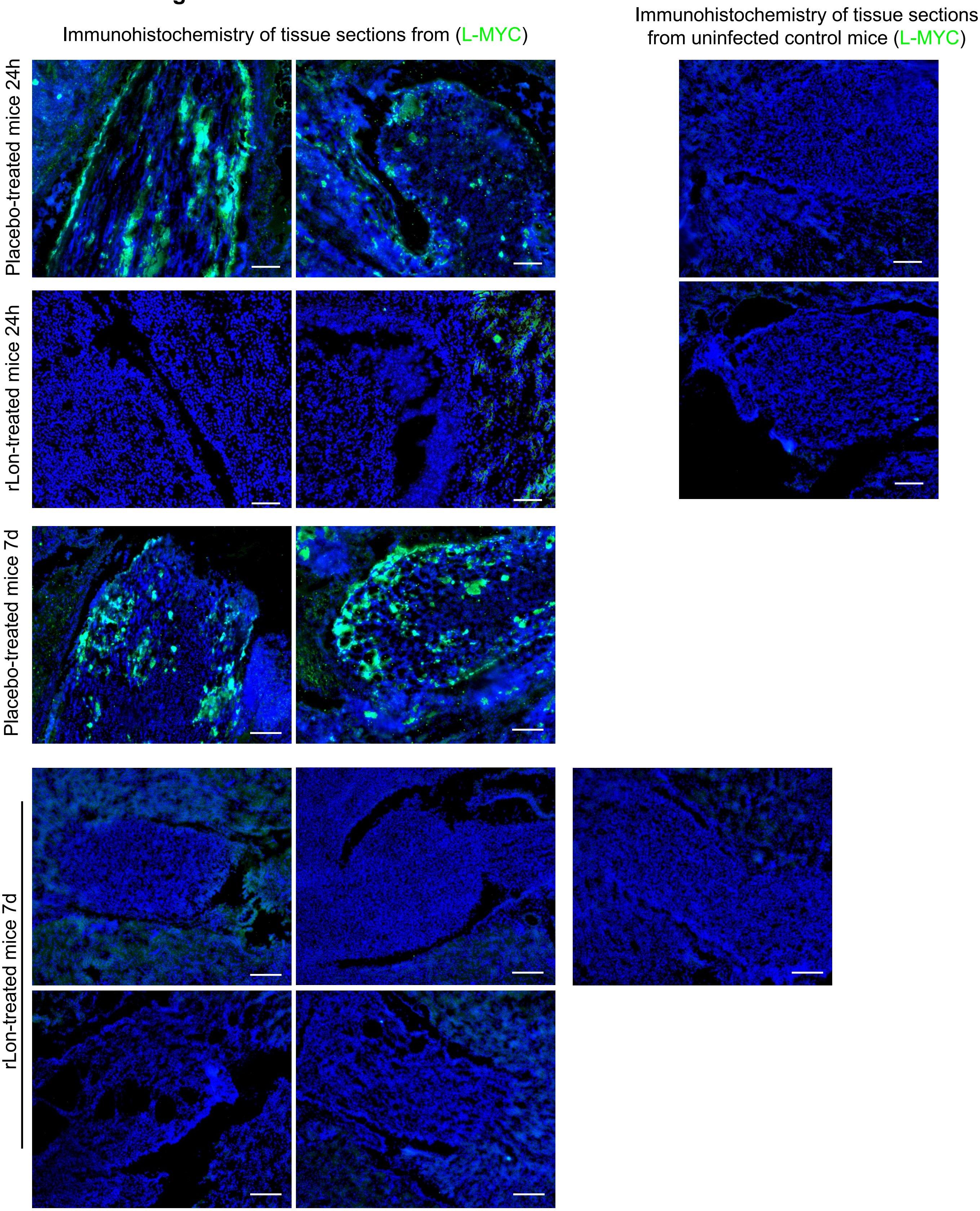

Extended Data Fig. 26. Supplementary Data for Main Fig. 5.
L-MYC protein staining in kidney tissue sections of the rLon-treated group compared to the placebo group. Confocal microscopy, representative images were shown in Main Fig. 4; *n* = 2-5 mice per group; green, N-MYC; blue, nuclei; scale bar, 20 µm.

Extended Data Fig. 27

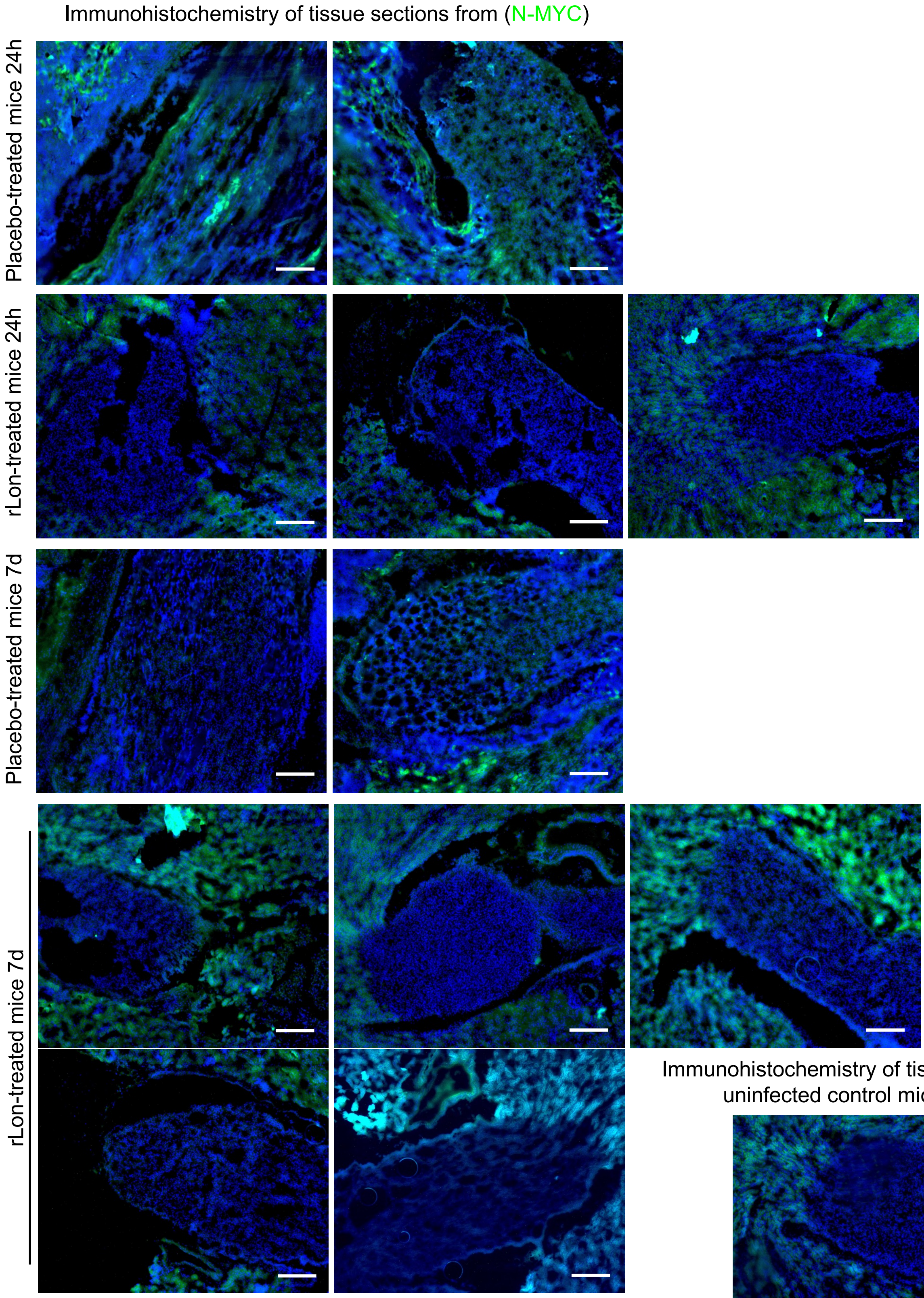

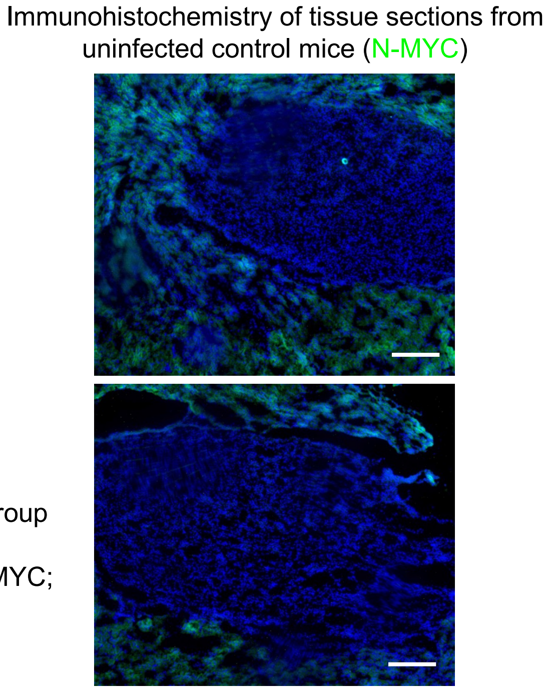

Extended Data Fig. 27. Supplementary Data for Main Fig. 5.
N-MYC protein staining in kidney tissue sections of the rLon-treated group compared to the placebo group. Confocal microscopy, representative images were shown in Main Fig. 4; *n* = 2-5 mice per group; green, N-MYC; blue, nuclei; scale bar, 20 µm.

Extended Data Fig. 28

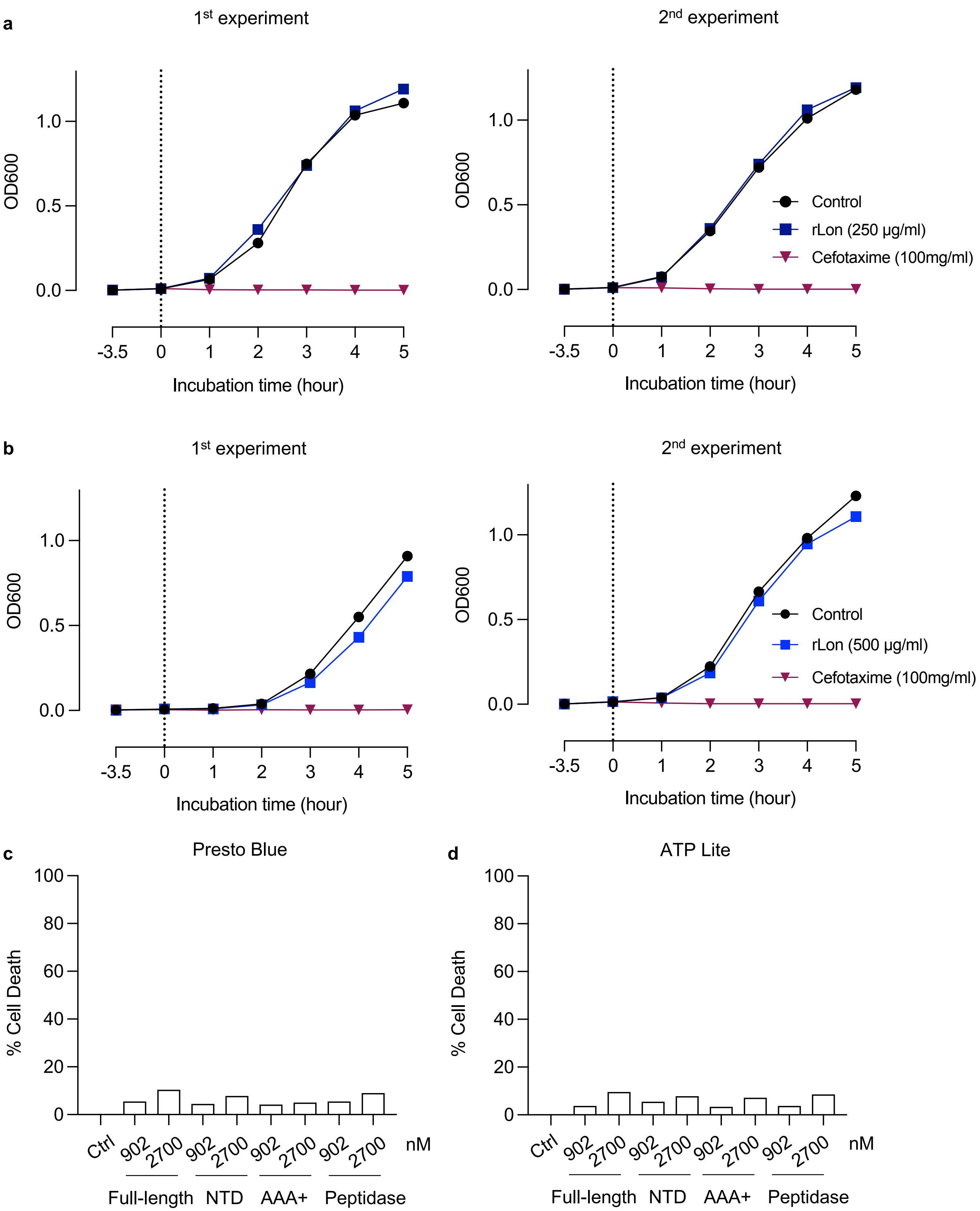

Extended Data Fig. 28. Lon effect on growth of *E. coli* CFT073 *in vitro*.

a, b, The Lon effect on growth of *E. coli* CFT073 *in vitro*. *E. coli* CFT073 (CFU =10^4) were cultured in Luria Broth media (black) for 3.5 hours followed by addition of 100 mg/mL of cefotaxime (red) or 250 µg/mL (dark blue) or 500 µg/mL (blue) of rLon. OD600 was measured at 0, 1, 2, 3, 4 and 5 hours. rLon did not affect the growth of *E. coli* CFT073. Two repeat experiments for each rLon concentration used together with cefotaxime as the positive control. **c, d,** Cell death assays for A498 kidney carcinoma cells based on Presto Blue (**c**) and ATP Lite (**d**) assays for full-length rLon and NTD, AAA+ and peptidase domain peptides. No toxicity was detected for full-length rLon, NTD, AAA+ nor peptidase domain peptide.

Extended Data Fig. 29

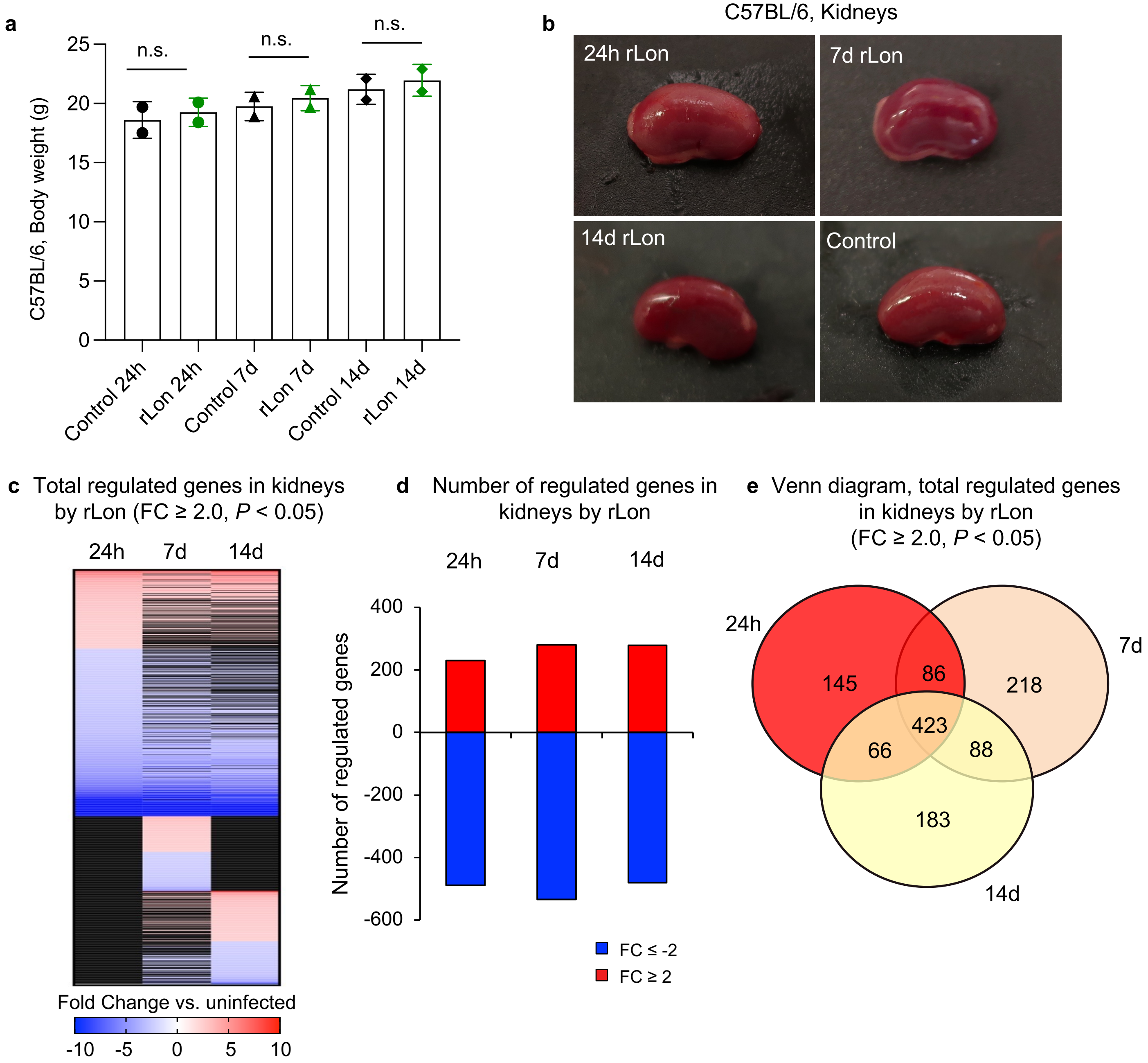

Extended Data Fig. 29. rLon treatment in healthy C57BL/6 mice.
a-d, Analysis of recombinant Lon protease (rLon) toxicity in healthy C57BL/6 mice. **a**, Mice were administered rLon intraperitoneally (100 µL, 250 µg/mL) once a day and sacrificed after 24 hours, 7 or 14 days. **a, b**, No change in body weight or macroscopic appearance of the kidney was detected (*n* = 2 mice per group). Scale bar = Scale bar = 5 mm, left kidney. Data are presented as mean ± s.d.. n.s. = Not significant. **c-e**, Heatmap (**c**), histogram (**d**) and Venn diagram (**e**) comparing the gene expression profiles of rLon-treated mice after 24 hours, 7 or 14 days to those of untreated mice (*n* = 2 per group; red, upregulated; blue, downregulated; cutoff FC ≥ 2, $P < 0.05$ compared to untreated mice).

Extended Data Fig. 30

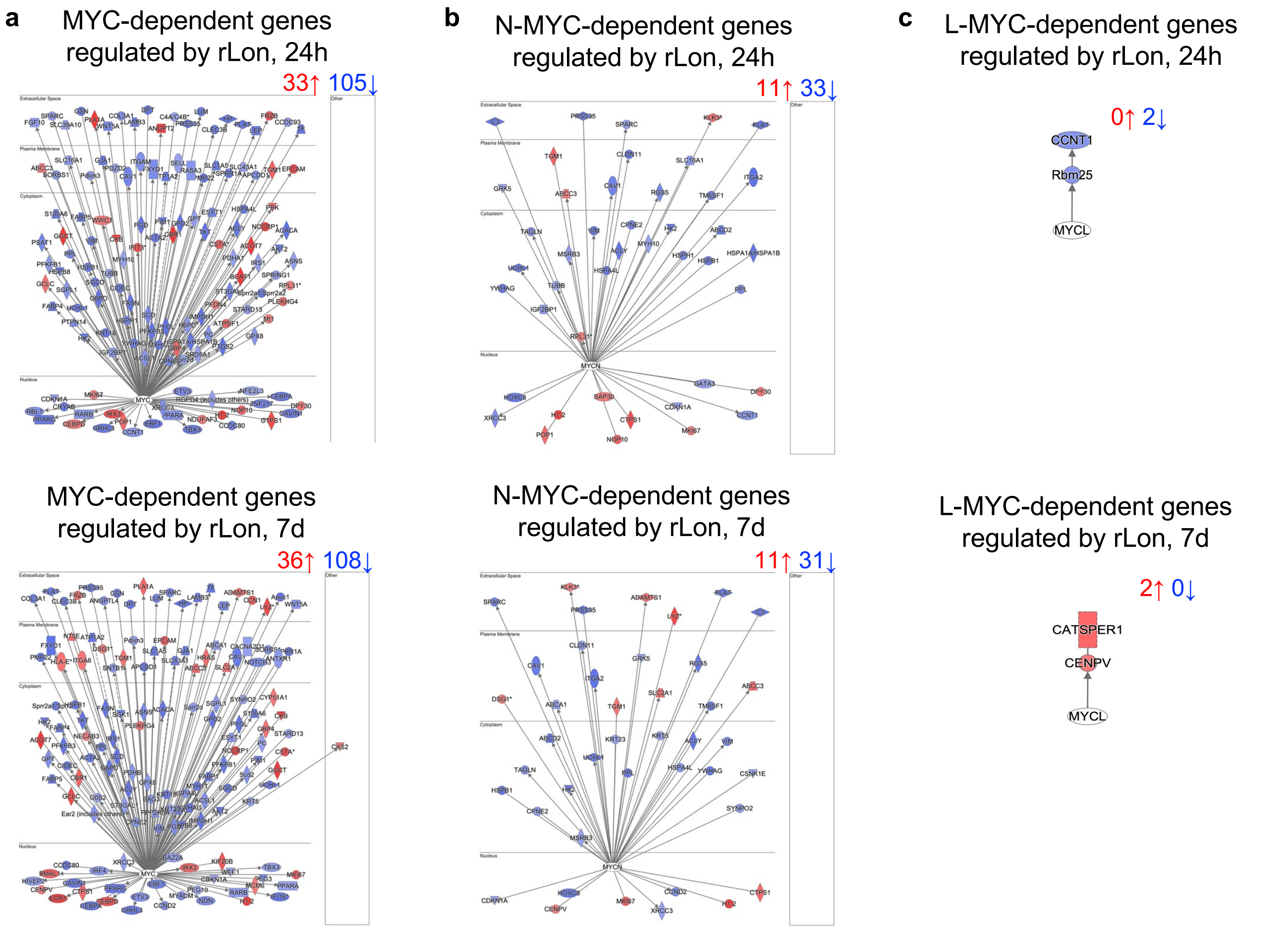

Extended Data Fig. 30. Control experiment for Main Fig. 5

a-c, Network analysis of c-MYC- (**a**), N-MYC- (**b**) and L-MYC (**c**)-dependent genes regulated by rLon in healthy C57BL/6 mice after 24 hours and seven days (*n* = 2 mice per group; cutoff FC ≥ 2; $P < 0.05$ compared to untreated mice). Modest regulation of c-MYC-dependent genes was observed after 24 hours and seven days with a predominance of inhibition. Few N-MYC- and L-MYC-dependent genes were regulated.

Extended Data Fig. 31

a Renal toxicity genes regulated in healthy kidneys

C57BL/6 rLon 24h

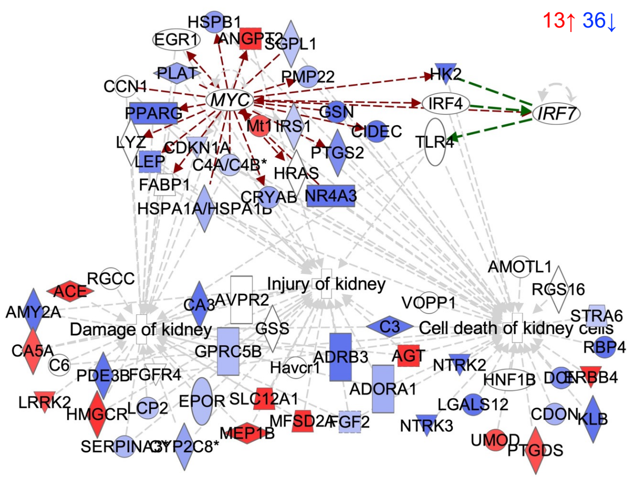

C57BL/6 rLon 7d

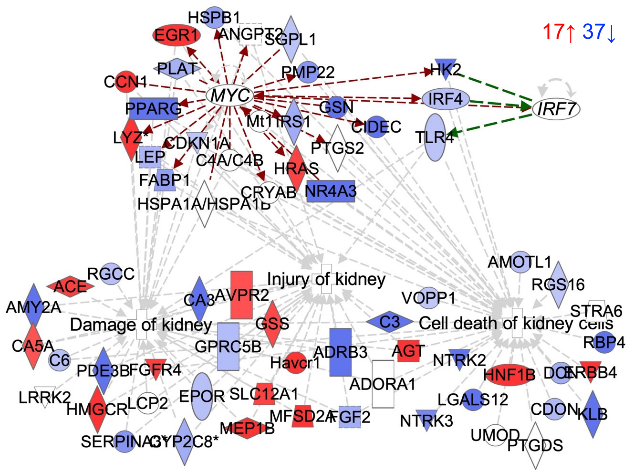

b Number of regulated renal toxicity genes in healthy kidneys

	rLon 24h		rLon 7d	
	**Total**	**MYC-related**	**Total**	**MYC-related**
Up	13	2	17	4
Down	36	16	37	15
Total	49	18	54	19

c Renal toxicity functions

	rLon 24h			rLon 7d		
**Diseases**	***P* value**	**Z score**	**Genes**	***P* value**	**Z score**	**Genes**
Kidney damage	7.8E-06	-0.4	23	1.2E-06	-0.7	26
Kidney injury	6.3E-06	0.3	20	5.8E-07	-0.2	23
Kidney cell death	4.6E-04	-0.4	30	2.1E-03	-1.3	30

d Top regulated shared renal toxicity genes in healthy kidneys

		Fold Change	
**Symbol**	**Gene Name**	**rLon 24h**	**rLon 7d**
Ca3	carbonic anhydrase 3	-73.9	-140.6
Cidec	cell death-inducing DFFA-like effector c	-31.1	-59.8
Lgals12	galectin 12	-21.2	-27.9
Rbp4	retinol binding protein 4	-10.3	-27.3
Pparg	peroxisome proliferator-activated receptor gamma	-19.3	-26.4
Ntrk2	neurotrophic receptor tyrosine kinase 2	-19.0	-24.3
Adrb3	adrenergic receptor, beta 3	-17.0	-22.4
Amy2a	amylase alpha 2a	-13.4	-21.1
Nr4a3	nuclear receptor subfamily 4 group A member 3	-12.3	-15.7
Hk2	hexokinase ii	-8.2	-8.2
Ca5a	carbonic anhydrase 5A	2.0	2.0
Slc12a1	solute carrier family 12 member 1	2.4	2.2
Ace	angiotensin converting enzyme	2.5	2.2
Erbb4	erb-b2 receptor tyrosine kinase 4	2.9	2.4
Agt	angiotensinogen	3.0	3.7
Mfsd2a	major facilitator superfamily domain containing 2	3.1	3.3
Hmgcr	3-hydroxy-3-methylglutaryl-CoA reductase	3.5	4.3
Mep1b	meprin 1 beta	5.0	5.9

Extended Data Fig. 31. Control experiment for Main Fig. 6.
a, b, Gene expression analysis identifying genes associated with renal toxicity, renal injury or kidney-specific cell death in rLon-treated healthy C57BL/6 mice. Lack of activation of MYC- and IRF7-dependent genes regulating renal injury or kidney-specific cell death in the rLon-treated group compared to the untreated group. Red, upregulated, blue, downregulated; cutoff FC ≥ 2; $P < 0.05$ compared to the uninfected group. **c,** Lack of renal toxicity functions by rLon in healthy C57BL/6 mice. **d,** Top regulated renal toxicity genes in kidneys of rLon-treated healthy C57BL/6 mice.

Extended Data Fig. 32

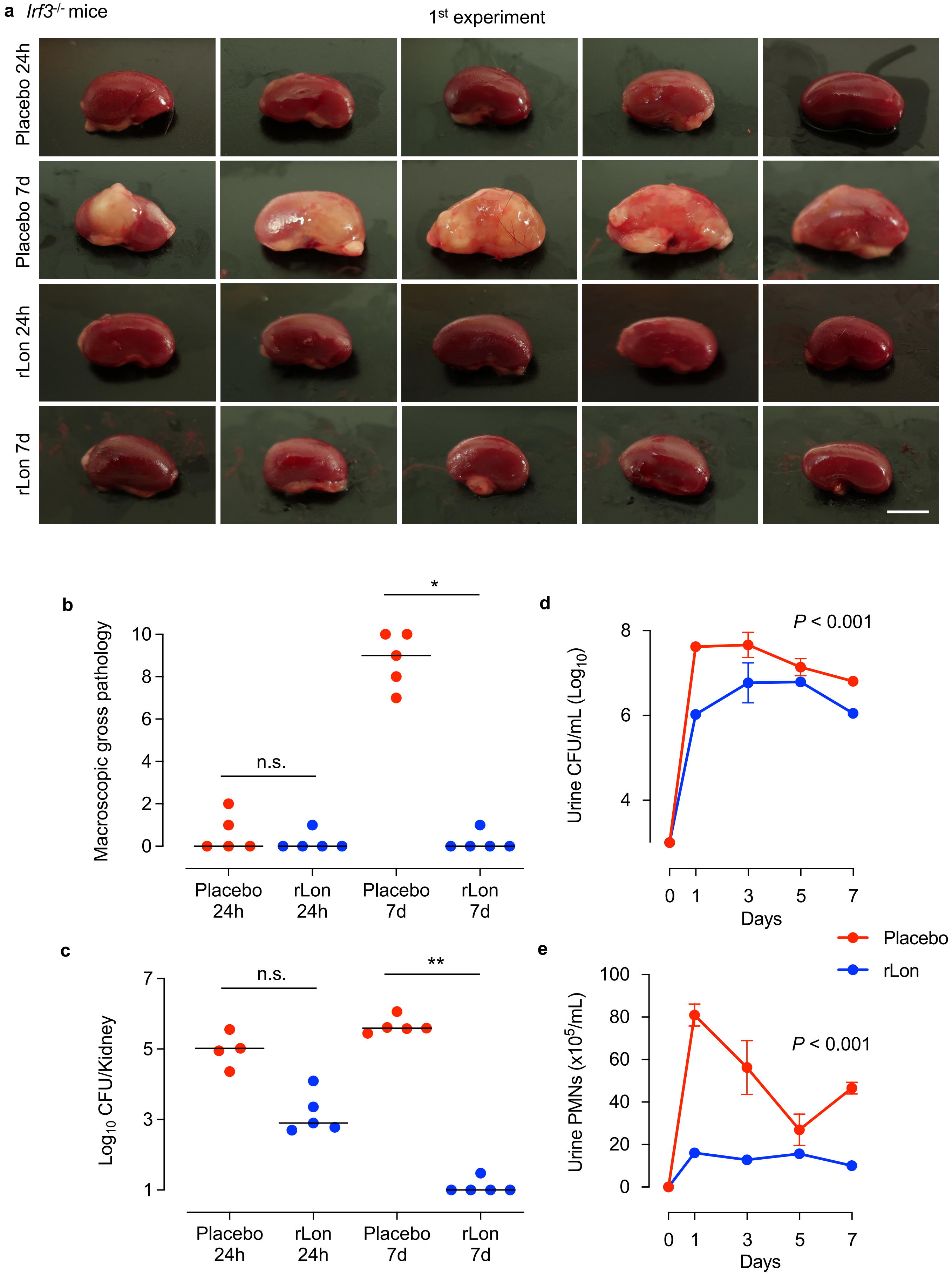

Extended Data Fig. 32

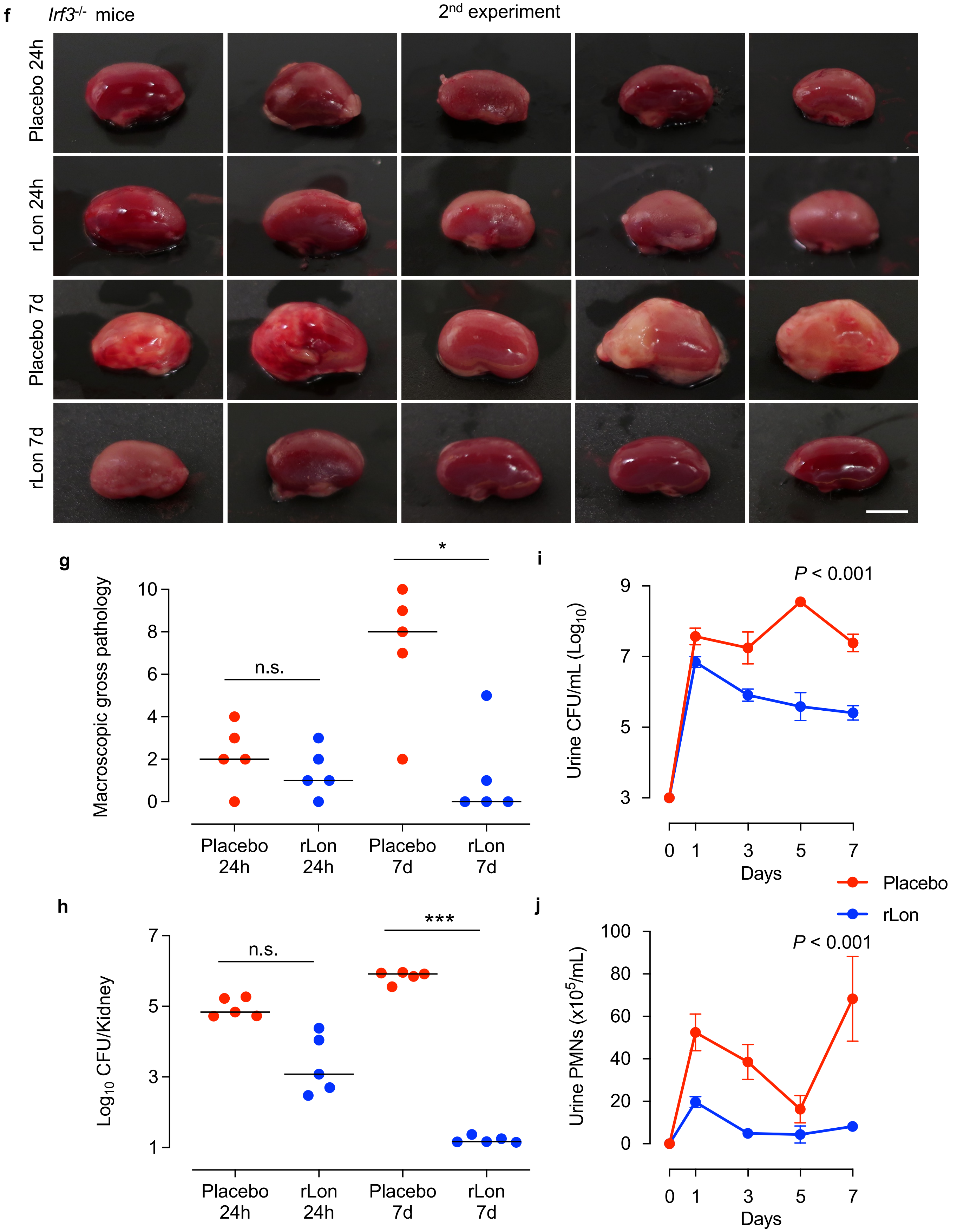

Extended Data Fig. 32. Supplementary Data for Main Fig. 6.
a, f, Macroscopic appearance of kidneys from *Irf3*$^{-/-}$ mice in the placebo or rLon treatment group. Scale bar = 5 mm, left kidney. **b-e, g-j,** Lower gross pathology, urine neutrophil count, and bacterial count in urine and renal tissues were compared in mice treated with rLon or placebo. The results of three separate experiments are shown. *n* = 5 mice per treatment for the 1st experiments and 2nd experiment. Lines represent medians (**b, c, g, h**), and data were analyzed by the Kruskal-Wallis test with Dunn's correction. Data are presented as the mean ± s.e.m. (**d, e, i, j**) after analysis by two-way ANOVA with Bonferroni's multiple comparison test. *$P < 0.05$, **$P < 0.01$, ***$P < 0.001$. n.s. = not significant.

Extended Data Fig. 33

a Renal toxicity genes regulated in infected kidneys

Irf3⁻/⁻ CFT073, 7d, Placebo

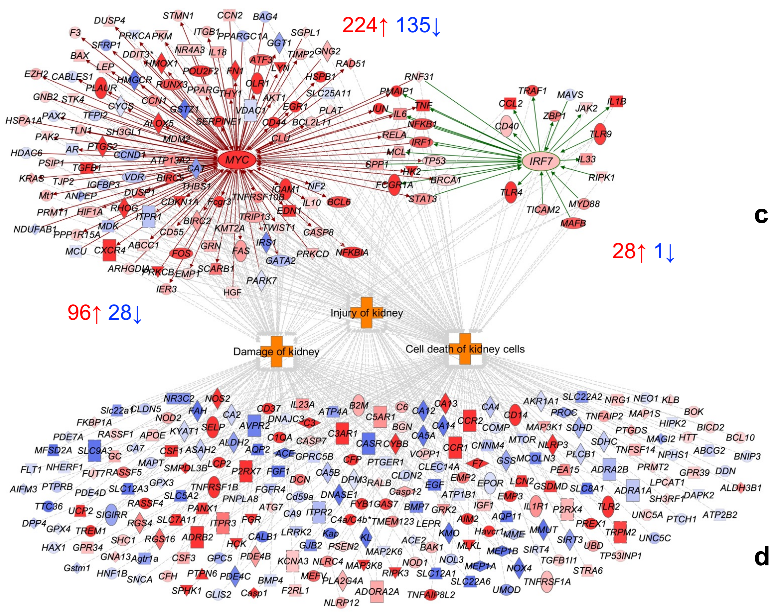

Irf3⁻/⁻ CFT073, 7d, rLon

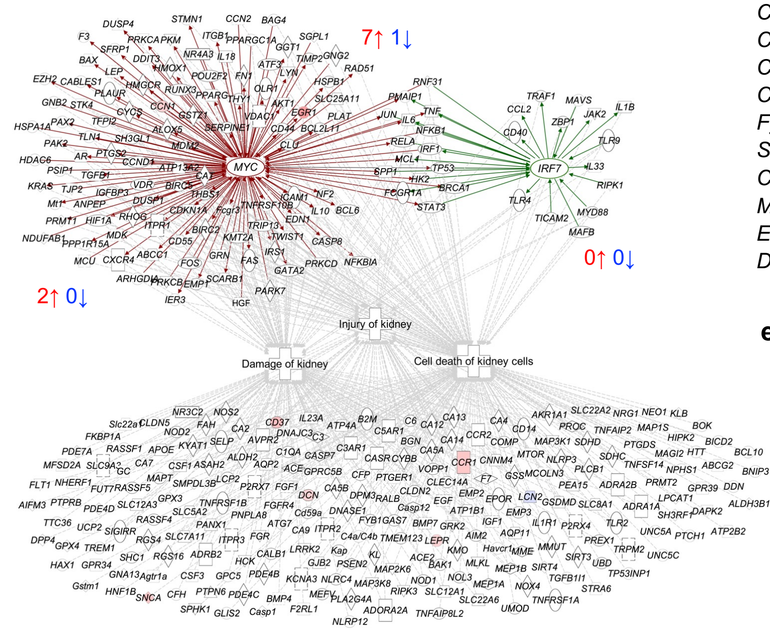

b Number of regulated renal toxicity genes in infected kidneys

	Placebo		rLon	
	**Total**	**MYC-related**	**Total**	**MYC-related**
Up	224	96	7	2
Down	135	28	1	0
Total	359	124	8	0

c Renal toxicity functions

	Placebo			rLon		
**Diseases**	***P* value**	**Z score**	**Genes**	***P* value**	**Z score**	**Genes**
Kidney damage	7.8E-33	2.94	152	1.2E-02	n.d.	5
Kidney injury	7.4E-23	2.61	113	2.2E-02	n.d.	4
Kidney cell death	7.0E-29	3.81	231	4.9E-01	n.d.	3

d Top regulated renal toxicity genes in infected kidneys

		Fold Change	
**Symbol**	**Gene Name**	**Placebo**	**rLon**
Lcn2	lipocalin 2	1187.6	-2.4
Havcr1	hepatitis A virus cellular receptor 1	880.2	
Cd44	CD44 molecule	470.7	
Cd37	CD37 molecule	393.0	3.0
Cfp	complement factor properdin	378.3	
C3	complement C3	304.7	
Cd14	CD14 molecule	276.8	
Fyb1	FYN binding protein 1	262.2	
Selp	selectin P	246.3	
C3ar1	complement C3a receptor 1	221.6	
Mep1b	meprin A subunit beta	-489.9	
Egf	epidermal growth factor	-738.1	
Dnase1	deoxyribonuclease 1	-2592.3	

e Principal Component Analysis with total detected genes

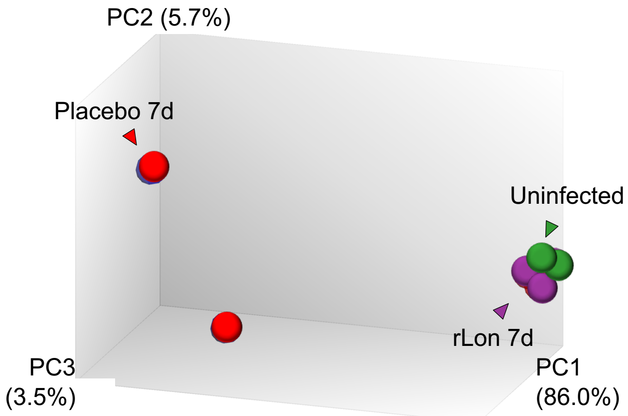

Extended Data Fig. 33. Supplementary Data for Main Fig. 6

a, Gene expression analysis identifying genes associated with renal toxicity, renal injury or kidney-specific cell death in *E. coli* CFT073-infected *Irf3*⁻/⁻ mice. Strong activation of MYC- and IRF7-dependent genes regulating renal injury or kidney-specific cell death in the placebo group compared to the uninfected group. Red, upregulated, blue, downregulated; cutoff FC ≥ 2; $P < 0.05$ compared to the uninfected group. **b,** Complete inhibition of genes regulating renal injury or kidney-specific cell death in the rLon-treated group compared to the placebo group. **c,** Inhibition of kidney damage, injury and cell death pathways by rLon treatment compared to placebo. **d,** Top regulated renal toxicity genes in the placebo group were not regulated in the rLon-treated group, with the exception of *Lcn2* and *Cd37*. **e,** Principal component analysis of the whole kidney RNA data sets. The right cluster shows uninfected mice (green) and the adjacent cluster shows the rLon-treated mice (purple). The left cluster shows the placebo group (red).
